# Supplementary material for: Control of fibrosis with enhanced safety via asymmetric inhibition of prolyl‐tRNA synthetase 1
Source: EMBO Mol Med. 2023 May 22;15(7):e16940. doi: 10.15252/emmm.202216940 (PMC10331583; doi:10.15252/emmm.202216940)
Supplement: Supplementary file 1 — Appendix [file EMMM-15-e16940-s003.pdf]

Appendix for

Control of fibrosis with enhanced safety via asymmetric inhibition of prolyl-tRNA synthetase 1

Ina Yoon, Sulhee Kim, Minjae Cho, Kyung Ah You, Jonghyeon Son, Caroline Lee, Ji Hun Suh, Da-Jeong Bae, Jong Min Kim, Sinae Oh, Songhwa Park, Sanga Kim, Seong Hyeok Cho, Seonha Park, Kyuhyeon Bang, Minjeong Seo, Jong Hyun Kim, Bongyong Lee, Joon Seok Park, Kwang Yeon Hwang*, Sunghoon Kim*

*Correspondence to: chahong@korea.ac.kr (K.Y.H.), sunghoonkim@yonsei.ac.kr (S.K.)

Table of contents

Appendix Figure S1……………………………………………………………………….2

Appendix Figure S2……………………………………………………………………….3

Appendix Figure S3……………………………………………………………………….4

Appendix Figure S1

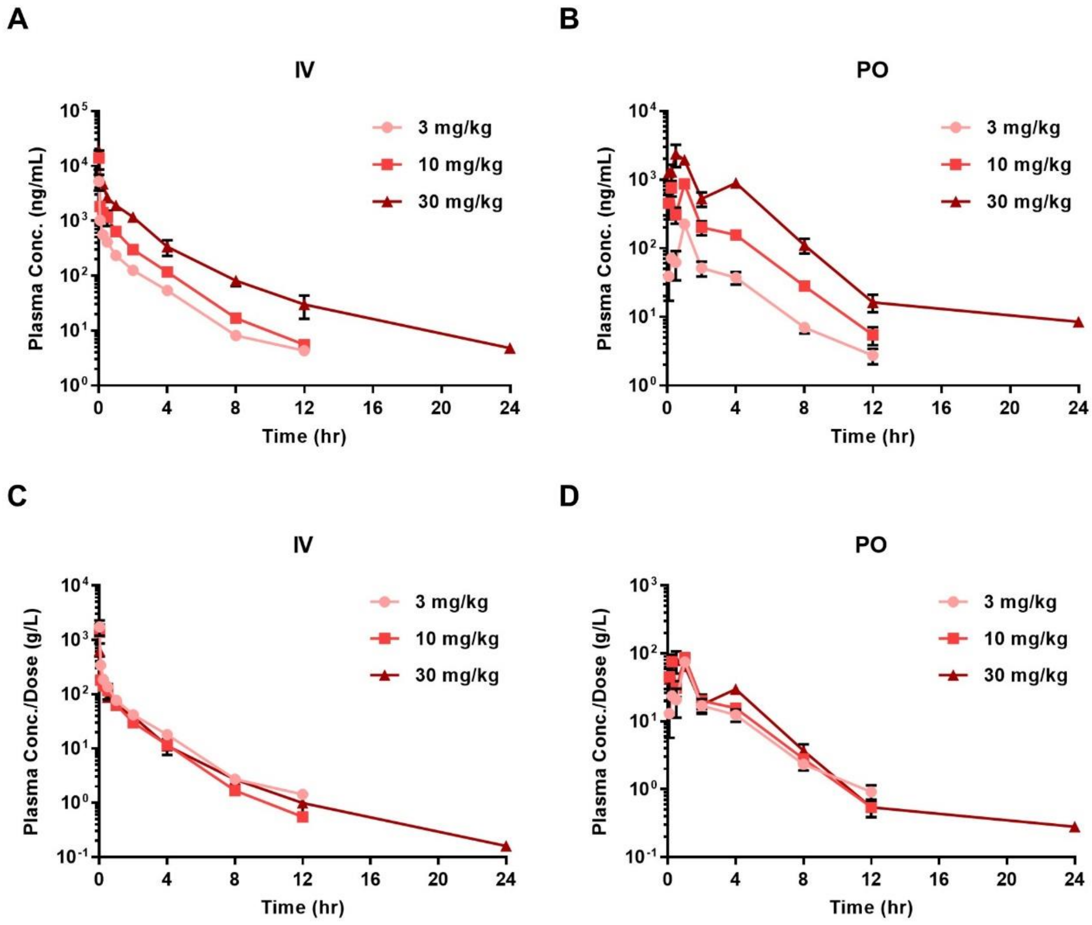

Appendix Fig S1. Pharmacokinetic study of DWN12088.

A, B. Plasma concentrations of DWN12088 at each time point were determined in mice intravenously (A) or orally (B) administered with the indicated dose of DWN12088 using LC-MS/MS (*n* = 3; mean ± SEM). IV, intravenous injection; PO, oral administration.

C, D. Dose-normalized plasma concentrations of DWN12088 at each time point were calculated based on (A) and (B).

Appendix Figure S2

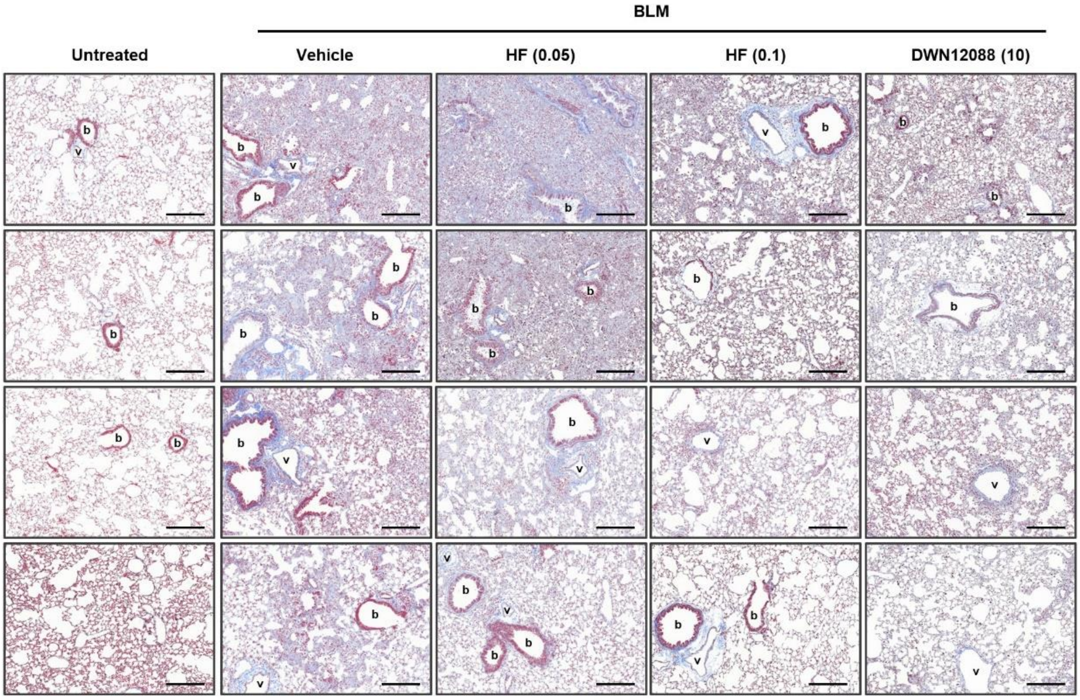

Appendix Fig S2. Comparison of *in vivo* efficacy of HF and DWN12088 in the collagen deposition of lung tissues obtained from the bleomycin-induced IPF model *in vivo*.

The *In vivo* efficacy of HF and DWN12088 was compared in a bleomycin-induced lung fibrosis model. The indicated concentrations of HF or DWN12088 were orally administered to mice once a day from a week after intratracheal injection of bleomycin. After administering the compounds for two weeks, collagen levels were determined by Masson's trichrome staining. Out of total five images, one is shown in Fig 1I (n = 1) and the rest are shown here (n = 4) (total, n = 5; scale bar = 200 μm). HF (0.05), HF 0.05 mg/kg; HF (0.1), HF 0.1 mg/kg; DWN12088 (10), DWN12088 10 mg/kg; BLM, bleomycin; b, bronchiole; v, blood vessel.

Appendix Figure S3

A

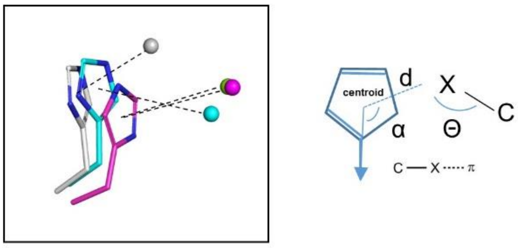

	Compound	d (Å)	α (°)	Θ (°)
Cl_green	HF Protomer A	3.36	96.0	110.9
Cl_magenta	HF Protomer B	3.36	92.1	115.7
Cl_cyan	DWN12088	4.26	81.2	98.9
F_gray	DWN11251	3.22	114.7	84.6

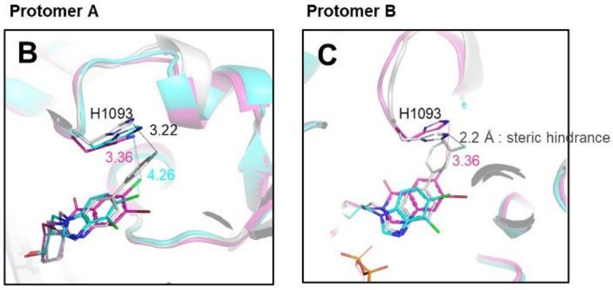

Appendix Fig S3. Comparison between H1093 and halogen atoms in each protomer.

A. Distance and angle between H1093 and halogen atoms of each compound. Various parameters related with halogen bond are summarized in the table.

B, C. Cartoon models near H1093 and halogen atoms of each compound. The individual protomers are superimposed. The color codes are same in (A). In protomer B, DWN compounds are predictive model when superimposed the ligands and model between HF and DWN compounds.
