# Supplementary material for: Control of fibrosis with enhanced safety via asymmetric inhibition of prolyl‐tRNA synthetase 1
Source: EMBO Mol Med. 2023 May 22;15(7):e16940. doi: 10.15252/emmm.202216940 (PMC10331583; doi:10.15252/emmm.202216940)
Supplement: Supplementary file 2 — Expanded View Figures PDF [file EMMM-15-e16940-s006.pdf]

Expanded View Figures

A

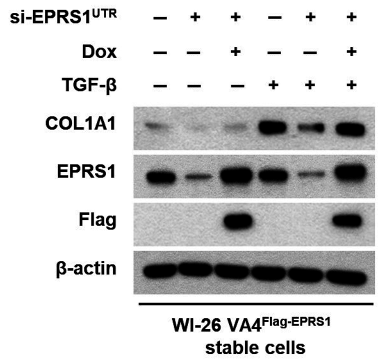

B

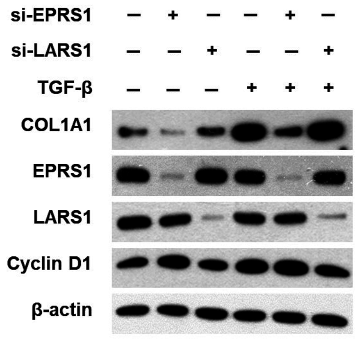

C

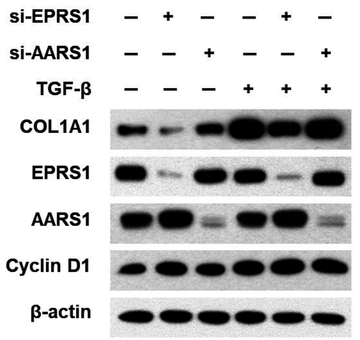

D

Protein	Proline %	Half-life* (h)
COL1A1	19.0	2.9
Cyclin D1	5.1	1.3
EPRS1	5.4	193
LARS1	5.1	262
AARS1	3.6	135
β-actin	5.1	61.9

Figure EV1. Significance of EPRS1 for cellular collagen synthesis.

A WI-26 VA4 cells were made to stably express Flag-EPRS1 in the presence of doxycycline (WI-26 VA4$^{Flag\text{-}EPRS1}$ stable cells). The cells were transfected with negative control or untranslated region (UTR) of EPRS1-targeting siRNAs for 72 h. The cells were also incubated in the presence of doxycycline for 68 h. After starved of serum for 6 h, the cells were treated with TGF-β for 15 h. The level of COL1A1 was determined by immunoblot assay. The level of β-actin was used as loading control.

B, C WI-26 VA4 cells were transfected with the indicated siRNAs for 72 h and incubated with TGF-β for 15 h. The level of COL1A1 was determined by immunoblot assay.

D The proportion of proline and the half-lives of proteins detected in (B) and (C) are listed in the table. The proportion of proline in the polypeptide sequence was calculated based on UniProtKB. The half-lives of proteins were cited from a study that measured half-lives of whole proteome in NIH3T3 cells via mass spectrometry (Schwanhausser *et al*, 2011).

Source data are available online for this figure.

 © 2023 The Authors

Figure EV2. Screening workflow and selection criteria of HF derivatives.

A Strategy used in screening for HF derivatives. In total, 523 synthesized compounds were tested for their efficacy in inhibiting the catalytic activity of PARS1 *in vitro*. Among them, 92 compounds which could inhibit PARS1 activity by more than 80% at 1 μM were further examined to obtain their IC_{50} values. Twenty-eight compounds that showed IC_{50} values lower than 100 nM were tested for their efficacy to reduce collagen in cell. Among them, 13 compounds that suppressed collagen levels by more than 50% at 1 μM were tested at multiple doses and 8 compounds showing IC_{50} values lower than 400 nM were tested for cytotoxicity. By comparing the IC_{50} values of collagen levels to the CC_{50} values, two compounds were selected and further tested in *in vivo* efficacy and *in vivo* toxicity models. DWN12088 was finally selected by considering both its efficacy and toxicity doses *in vivo*.

B Heat-map of the inhibitory activities of 523 DWN compounds in *in vitro* prolylation assay at 1 μM.

C Heat-map of the inhibitory activities of 28 DWN compounds to collagen levels in 3D cell culture at 1 μM.

D IC_{50} values for collagen level in 3D cell culture and CC_{50} values of eight compounds were listed in the table. The ratio of CC_{50} to collagen IC_{50} was calculated to estimate TI.

E–H *In vivo* Efficacy of DWN compounds was determined in a transverse aortic constriction model. Pirfenidone was used as a positive control for anti-fibrotic effect. The thickness of left ventricle (E), percentage of perivascular (F) and interstitial (G) collagen fiber, and number of infiltrated inflammatory cells (H) were determined as a measurement of fibrosis. DWN compounds and pirfenidone were administered at 10 mg/kg/day and 200 mg/kg/day, respectively ($n = 10$; One-way ANOVA; $^{***}P < 0.001$; mean ± SEM).

I *In vivo* toxicity of DWN10290 was determined in 2-week repeated dose toxicity study. HDW, hemoglobin concentration distribution width; RDW, red cell distribution width; MPV, mean platelet volume; MONO, monocyte; HGB, hemoglobin concentration; HCT, hematocrit; MCH, mean cell hemoglobin; MCHC, mean cell hemoglobin concentration; RBC, red blood cell; WBC, white blood cell; LYMP, lymphocyte; TBIL, total bilirubin; ALP, alkaline phosphatase; TG, triglyceride; HDL, high density lipoprotein; LDL, low density lipoprotein; CHO, cholesterol; BUN, blood urea nitrogen.

Source data are available online for this figure.

© 2023 The Authors

A

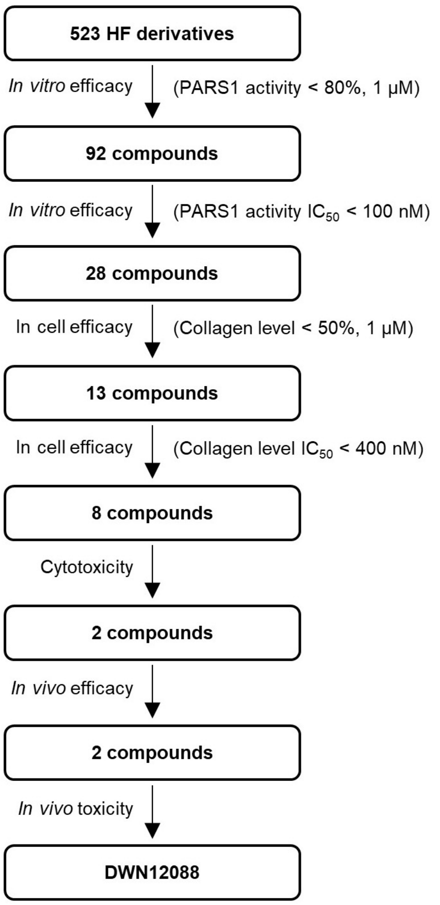

B C

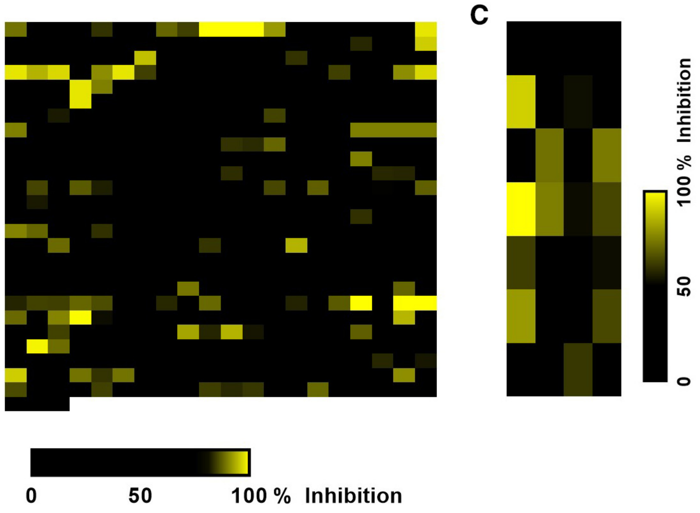

D

Compound	IC_{50} for collagen level (μM)	CC_{50} (μM)	CC_{50}/collagen IC_{50}
DWN10290	0.07398	77.05	1041
DWN10620	0.2150	29.28	136
DWN10993	0.04804	45.64	950
DWN12088	0.04307	> 100	> 2321
DWN12787	0.2745	> 100	> 364
DWN12923	0.2919	69.70	239
DWN12949	0.14750	> 100	> 678
DWN13012	0.10580	74.47	704

E F G H

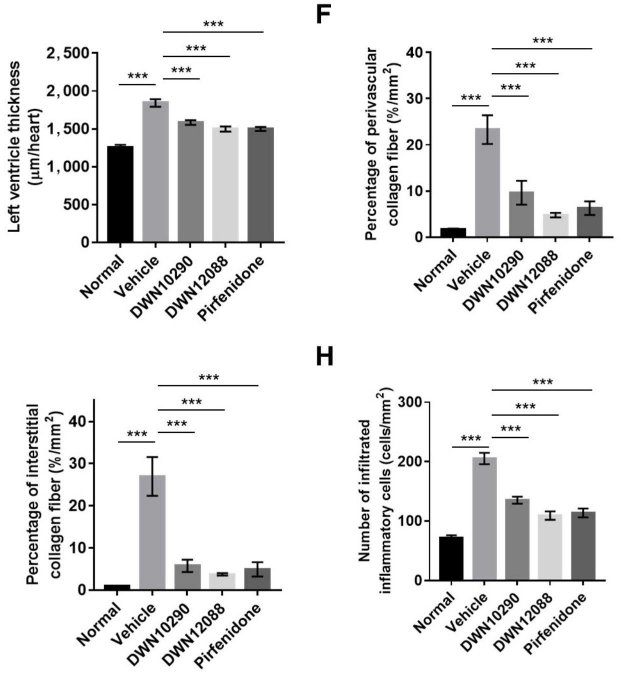

I

		DWN10290		
Dose level (mg/kg)		10	20	50
Mortality		-	-	Found dead: 1/5 (on day 9)
Clinical signs		-	Diarrhea	Diarrhea, Inanimation
Body weight		-	-	-
Hematology	↑	HDW	RDW, HDW	RDW, HDW, MPV, MONO
	↓	-	HGB, HCT, MCH, MCHC	RBC, HGB, HCT, WBC, LYMP
Clinical chemistry	↑	-	TBIL	ALP
	↓	HDL, LDL, CHO, BUN	TG, HDL, LDL, CHO, BUN	TG, HDL, LDL, CHO, BUN
Organ weight	↑	-	-	Liver, Kidney
	↓	-	-	Thymus
Necropsy finding		• Pale liver (4/5)	• Pale liver (1/5)	• Pale liver (4/5) • Spleen atrophy (1/5)

Figure EV2.

 © 2023 The Authors

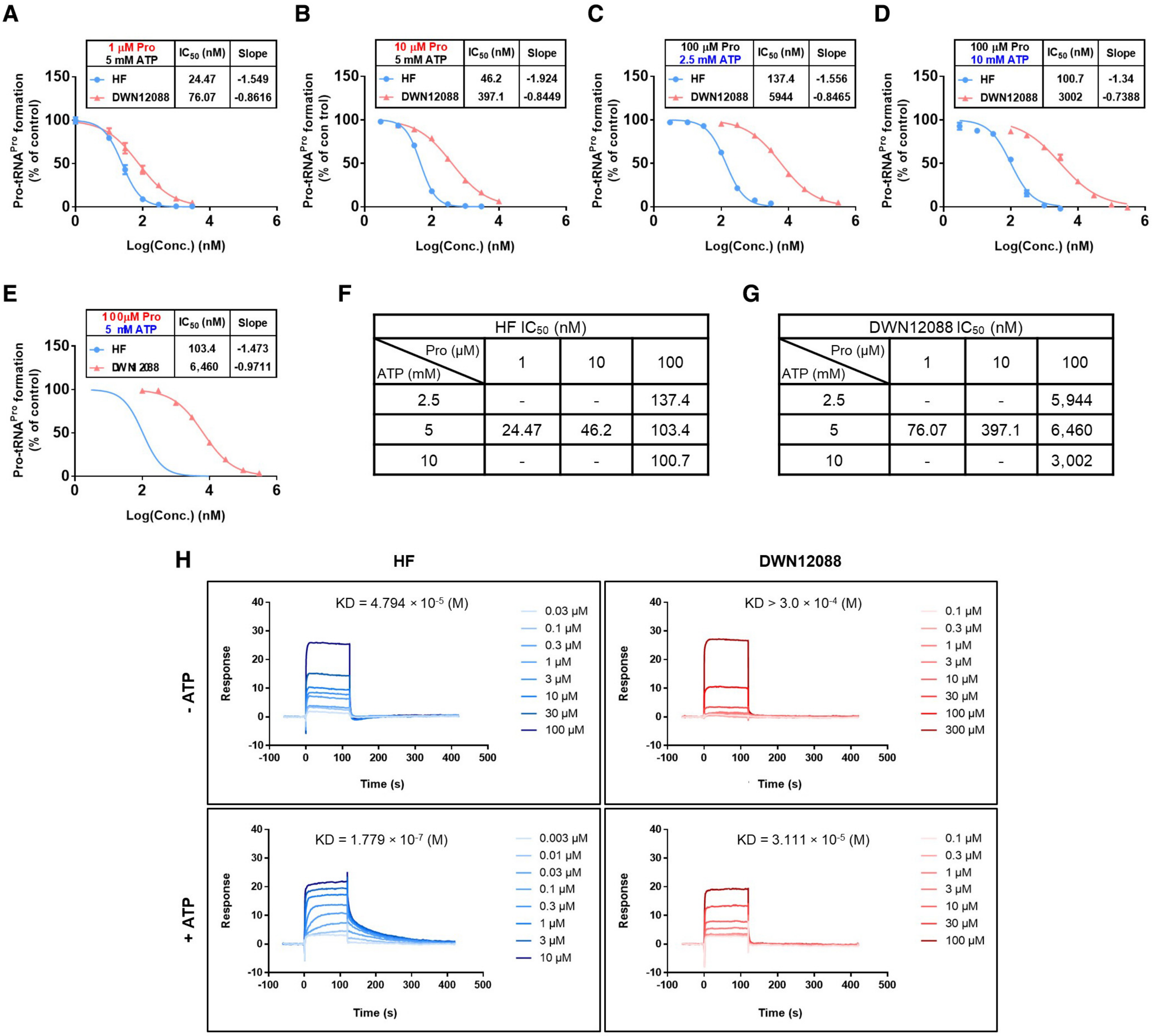

F

HF IC50 (nM)			
ATP (mM) \ Pro (µM)	1	10	100
2.5	-	-	137.4
5	24.47	46.2	103.4
10	-	-	100.7

G

DWN12088 IC50 (nM)			
ATP (mM) \ Pro (µM)	1	10	100
2.5	-	-	5,944
5	76.07	397.1	6,460
10	-	-	3,002

Figure EV3. Kinetic and binding properties of HF and DWN12088.

A–E The inhibitory activities of HF and DWN12088 were monitored by determining the catalytic activities of PARS1 under the indicated concentrations of proline and ATP. The IC_{50} values and slopes are listed in the table (A (HF), n = 5 from two independent experiments (triplicate for one experiment and duplicate for one experiment); A (DWN12088), n = 7 from three independent experiments (triplicate for one experiment and duplicate for two experiments); (B–D) technical replicate n = 3; (E) n = 9 from three independent experiments (triplicate for each experiment); mean ± SEM).

F, G IC_{50} values of HF and DWN12088 for *in vitro* prolylation assay were listed in the tables.

H The interaction of PARS1 and the indicated compounds in the presence or absence of ATP was determined via SPR. Affinity (KD) of the compound to PARS1 is shown on the top of the sensorgram.

Source data are available online for this figure.

Figure EV4. *In vivo* efficacy of DWN12088.

A–C The *in vivo* efficacy of DWN12088 was determined in a BLM-induced lung fibrosis model. The indicated compounds were administered a day before oropharyngeal administration of BLM. DWN12088 was administered at 3, 10, 30 mg/kg once a day and pirfenidone was administered at 100 mg/kg twice a day (200 mg/kg per day). After administering the compounds for 3 weeks, modified Ashcroft score (A) (Hubner *et al*, 2008), lung weight (B), and collagen content in BALF (C) were determined (n = 10; Mann–Whitney test after Kruskal–Wallis test; $*P < 0.05$, $**P < 0.01$; mean ± SEM). BLM, bleomycin; Pir, pirfenidone; NS, not significant.

D–F The *in vivo* efficacy of DWN12088, nintedanib and pirfenidone was compared in a BLM-induced lung fibrosis model. DWN12088 10 mg/kg, nintedanib 60 mg/kg and pirfenidone 200 mg/kg were orally administered to mice once a day from a week after intratracheal injection of BLM. After administering the compounds for 2 weeks, SpO_2 (D), BALF total cell count (E) and collagen contents (F) were determined (n = 9; Mann–Whitney test after Kruskal–Wallis test; $*P < 0.05$, $**P < 0.01$, $***P < 0.001$; mean ± SEM). BLM, bleomycin; NS, not significant.

G–I The *in vivo* efficacy of HF and DWN12088 was compared in a BLM-induced lung fibrosis model. HF (0.05 and 0.1 mg/kg), and DWN12088 (10 mg/kg) were orally administered to mice once a day from 2 weeks after intratracheal injection of BLM. After administering for 2 weeks, SpO_2 (G), BALF total cell count (H), collagen contents (I) were determined (n = 9; Mann–Whitney test after Kruskal–Wallis test; mean ± SEM). BLM, bleomycin; 12088, DWN12088.

Source data are available online for this figure.

 © 2023 The Authors

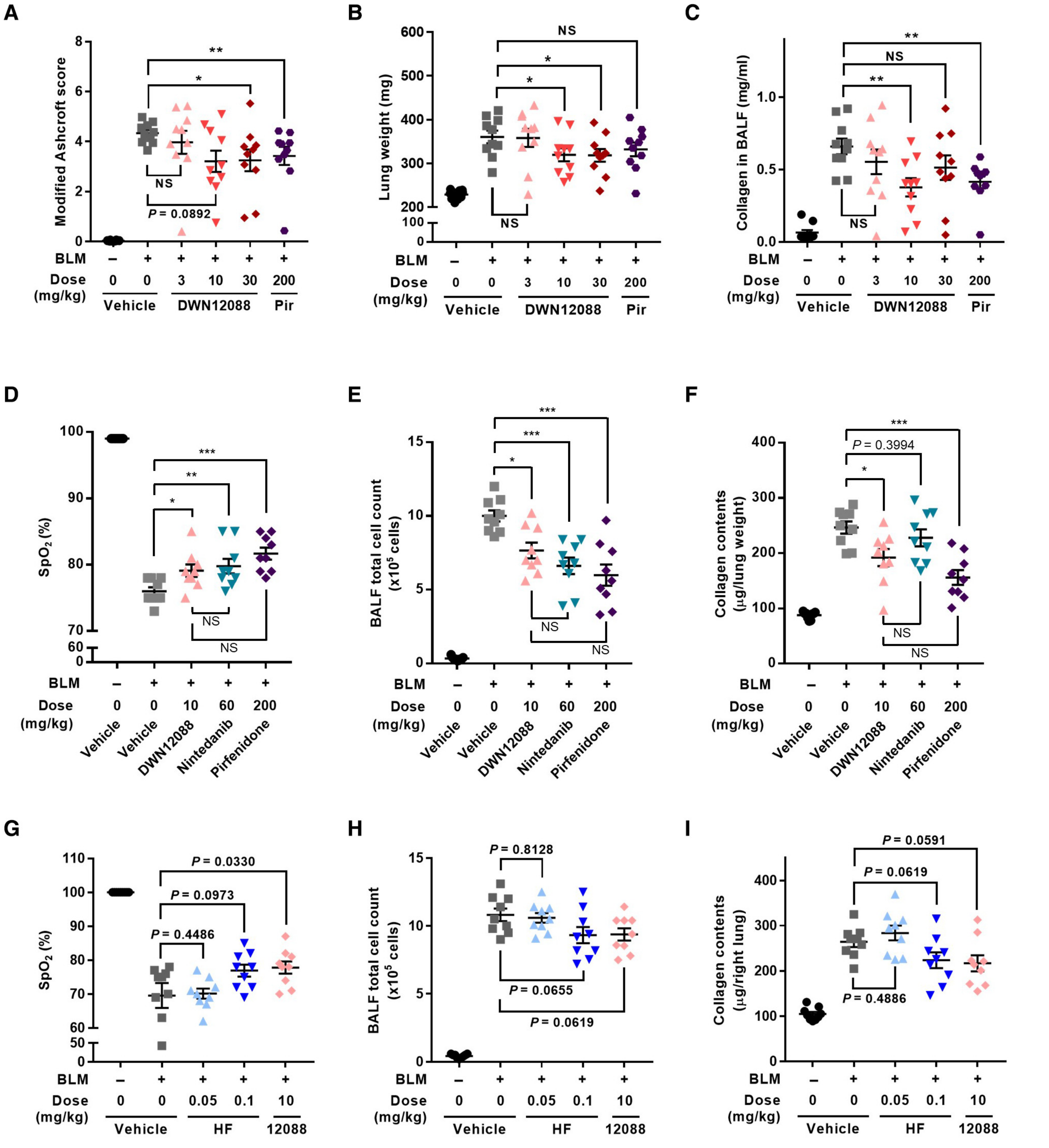
A
Modified Ashcroft score
NS
P = 0.0892
*
**
BLM
Dose (mg/kg)
Vehicle
DWN12088
Pir
B
Lung weight (mg)
NS
*
*
NS
C
Collagen in BALF (mg/ml)
**
NS
**
NS
D
SpO2 (%)
*
**
***
NS
NS
Vehicle
Vehicle
DWN12088
Nintedanib
Pirfenidone
E
BALF total cell count (x10^5 cells)
*
***
***
NS
NS
F
Collagen contents (µg/lung weight)
***
P = 0.3994
*
NS
NS
G
SpO2 (%)
P = 0.0330
P = 0.0973
P = 0.4486
HF
12088
H
BALF total cell count (x10^5 cells)
P = 0.8128
P = 0.0655
P = 0.0619
I
Collagen contents (µg/right lung)
P = 0.0591
P = 0.0619
P = 0.4886

Figure EV4.

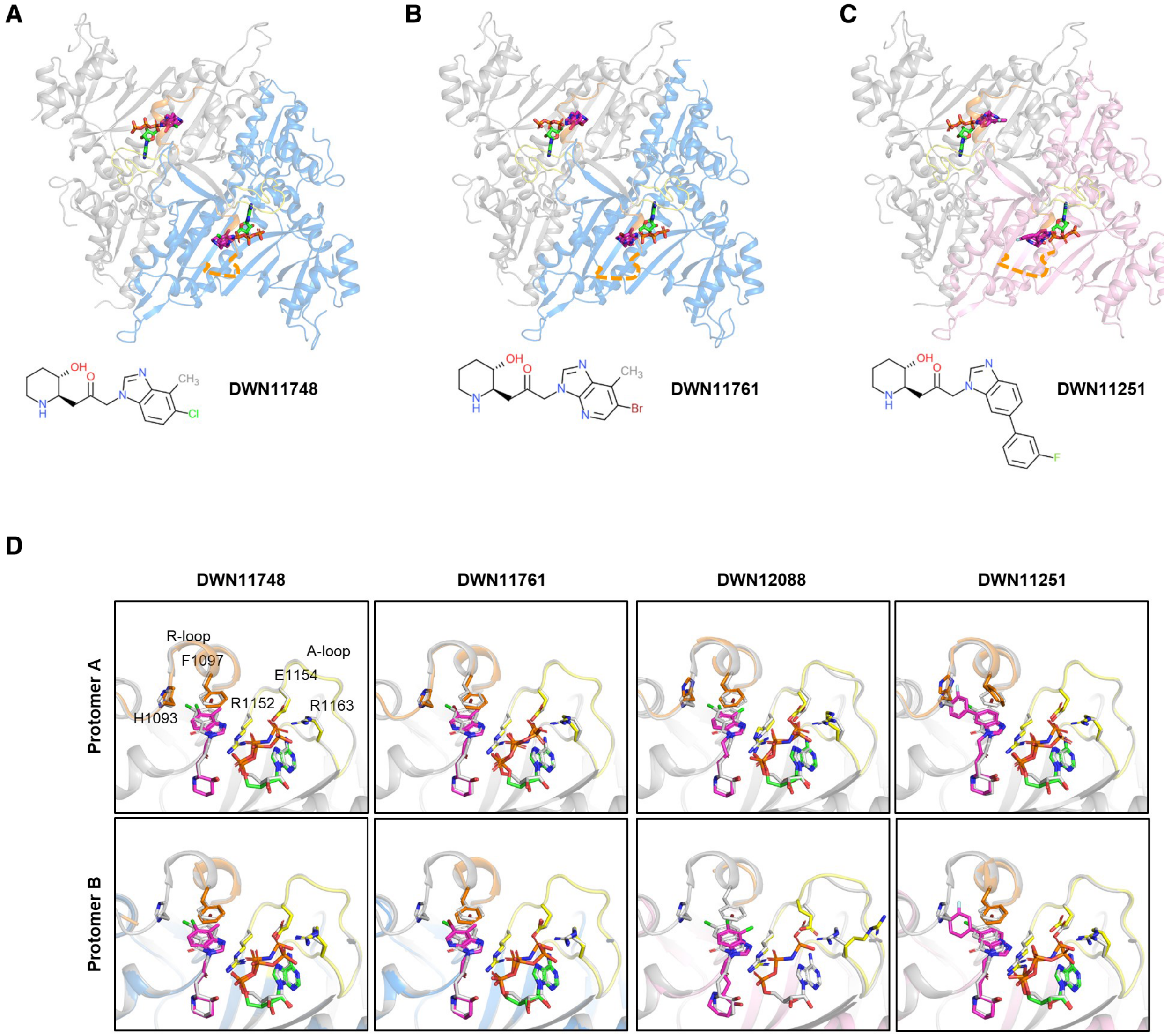

Figure EV5. The crystal structures of PARS1 complexed with DWN compounds.

A–C Overall structures of PARS1 complexed with the indicated compounds. PARS1 shows two types of the compound-binding states (rigid and loose binding). PARS1 chains with rigid binding are displayed in gray and blue whereas those with loose binding are displayed in pink. ATP is designated as green, and the compounds are designated as magenta stick models. R-loop (K1091 – F1097) and A-loop (R1152 – R1165) are presented as orange and yellow, respectively. The disordered region of R-loop is presented as orange dashed lines.

D Superposition of structures bound to DWN compounds and HF at the residues important for the interaction with compounds and ATP. DWN compound-bound structures are superposed with the F1097, R1152, E1154 and R1163 of 4HVC, which are represented as transparent gray stick models. The color codes of the structures are the same as those used in Fig 3A and B.

 © 2023 The Authors
