# Supplementary material for: Control of fibrosis with enhanced safety via asymmetric inhibition of prolyl‐tRNA synthetase 1
Source: EMBO Mol Med. 2023 May 22;15(7):e16940. doi: 10.15252/emmm.202216940 (PMC10331583; doi:10.15252/emmm.202216940)
Supplement: Supplementary file 3 — Source Data for Expanded View [file EMMM-15-e16940-s002.zip › EMM-2022-16940-Figure_6E_Source_Data.pdf]

Figure 6E

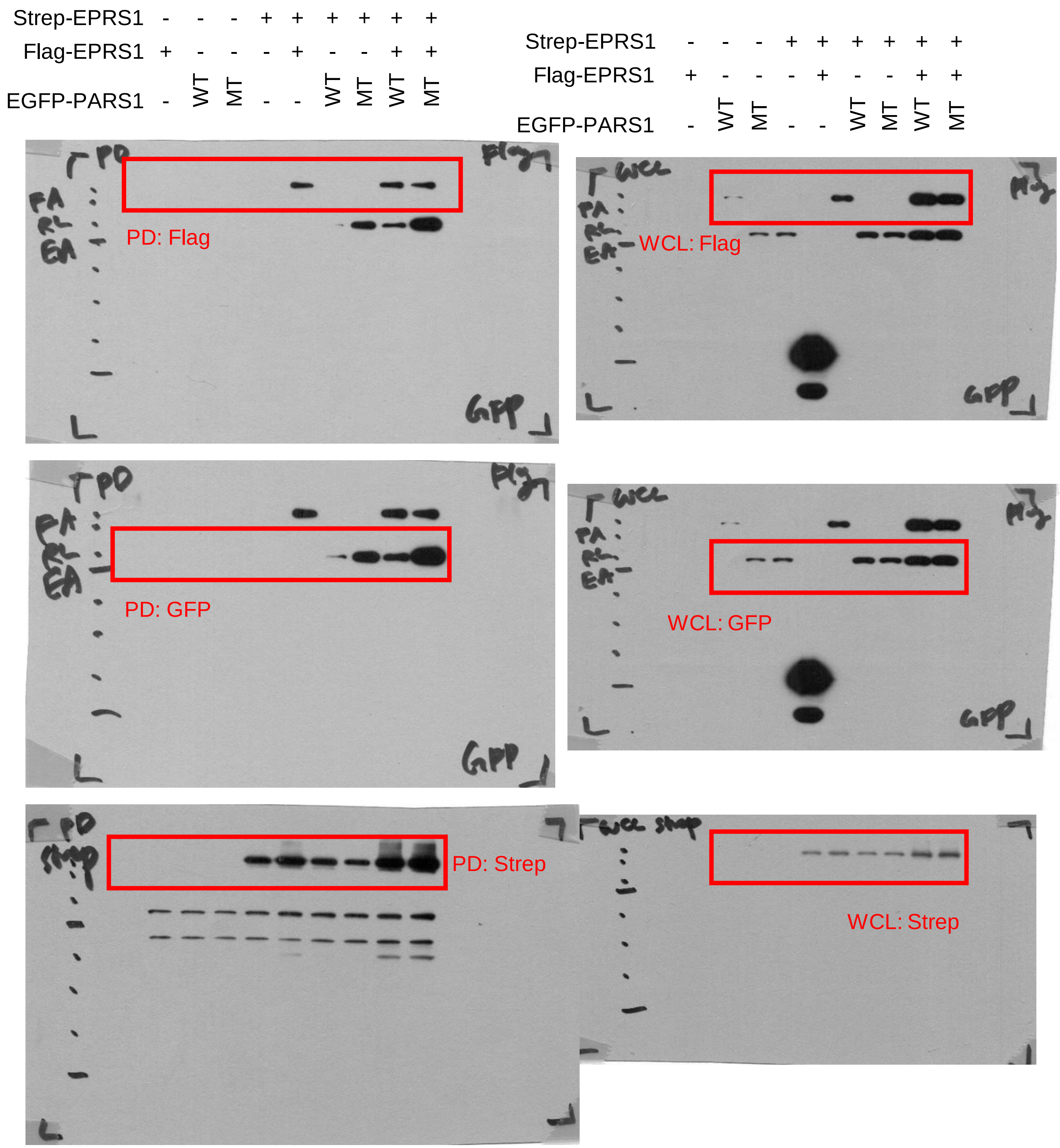

Strep-EPRS1 - - - + + + + + +
Flag-EPRS1 + - - - + - - + +
EGFP-PARS1 - WT MT - - WT MT WT MT
Strep-EPRS1 - - - + + + + + +
Flag-EPRS1 + - - - + - - + +
EGFP-PARS1 - WT MT - - WT MT WT MT
PD: Flag
WCL: Flag
PD: GFP
WCL: GFP
PD: Strep
WCL: Strep
