# Supplementary figures and images for: Control of fibrosis with enhanced safety via asymmetric inhibition of prolyl‐tRNA synthetase 1
Source: EMBO Mol Med. 2023 May 22;15(7):e16940. doi: 10.15252/emmm.202216940 (PMC10331583; doi:10.15252/emmm.202216940)

Figure 6K

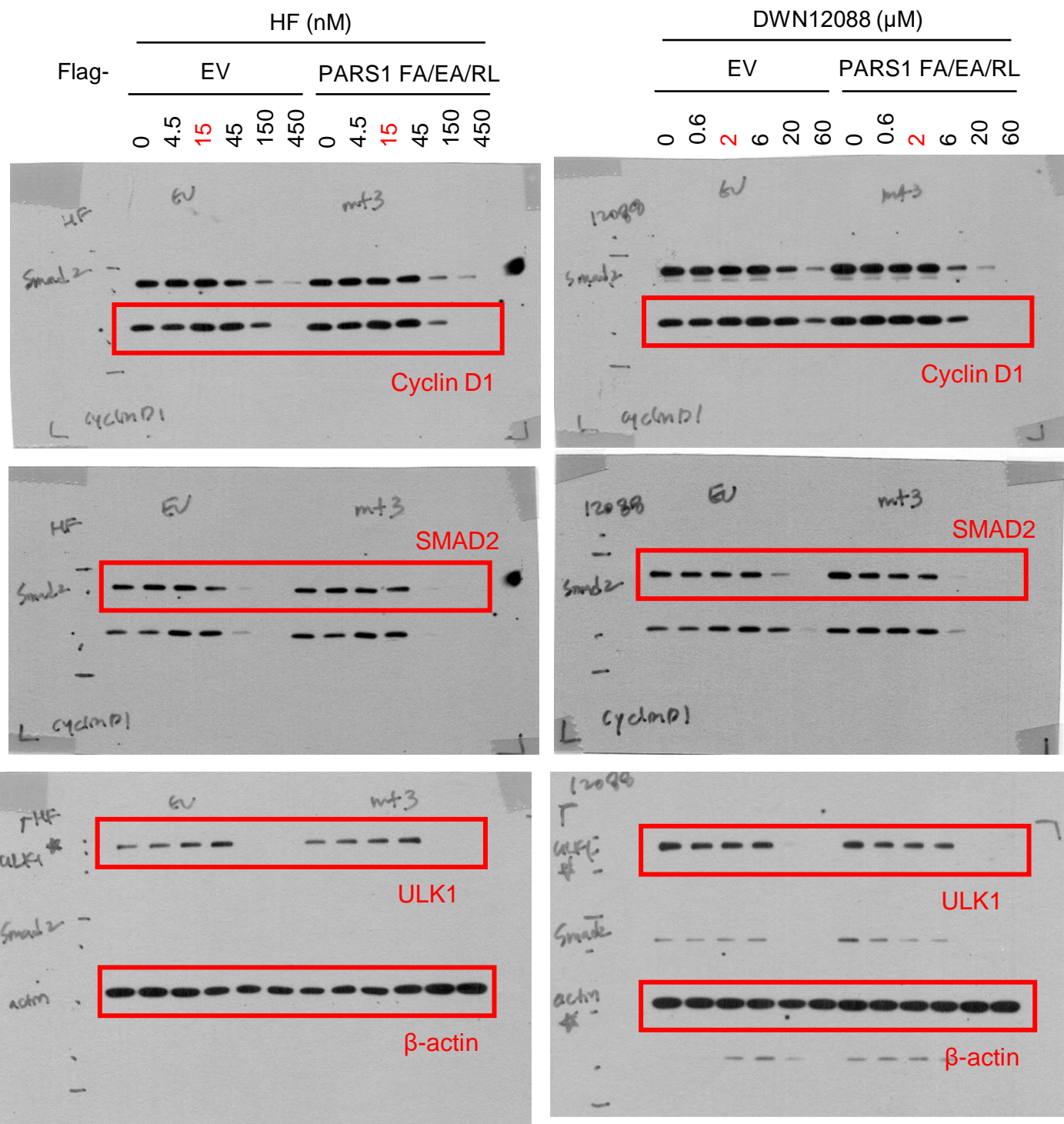

Figure 6K

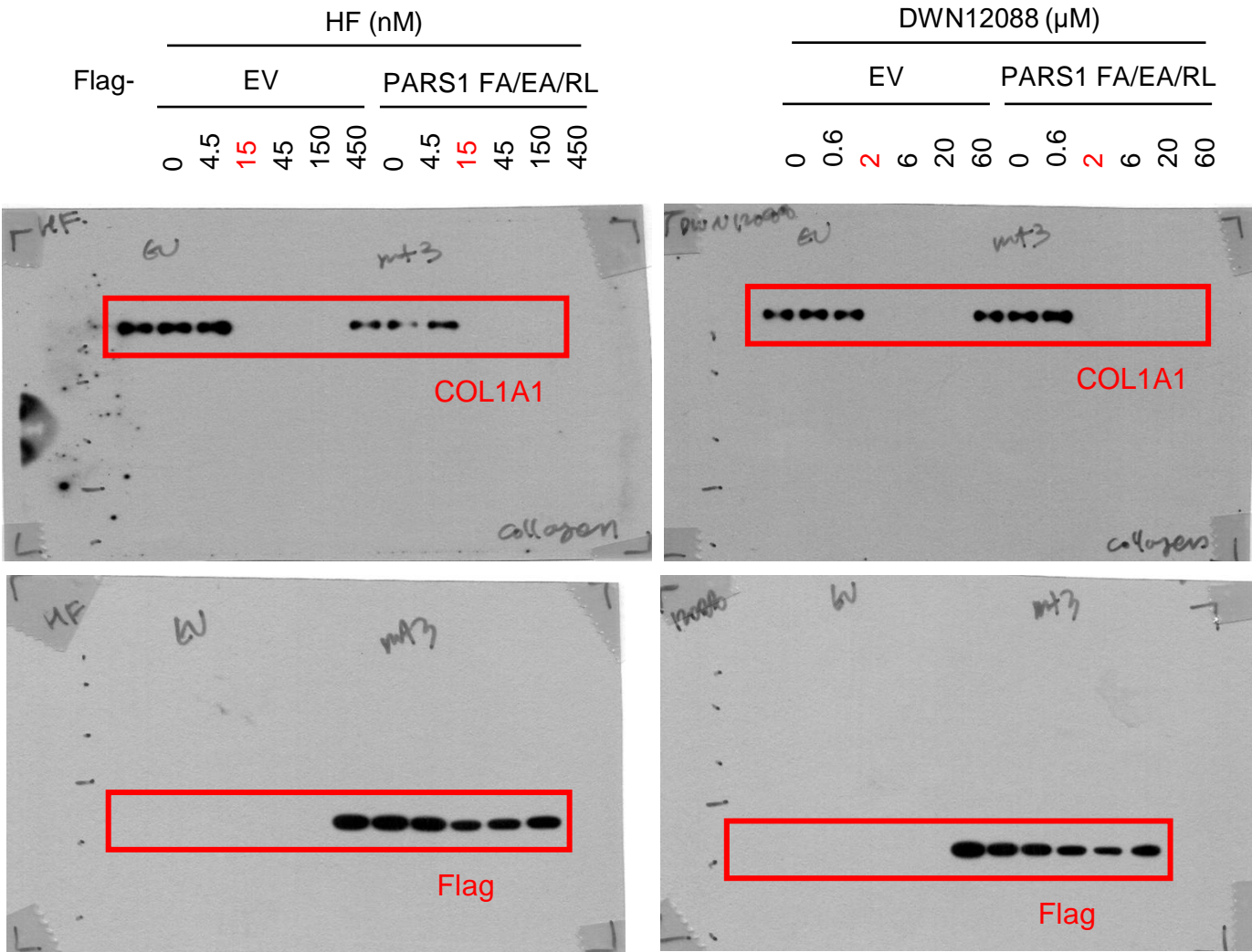

Supplement: Supplementary file 3 — Source Data for Expanded View [file EMMM-15-e16940-s002.zip › EMM-2022-16940-Figure_6K_Source_Data.pdf]

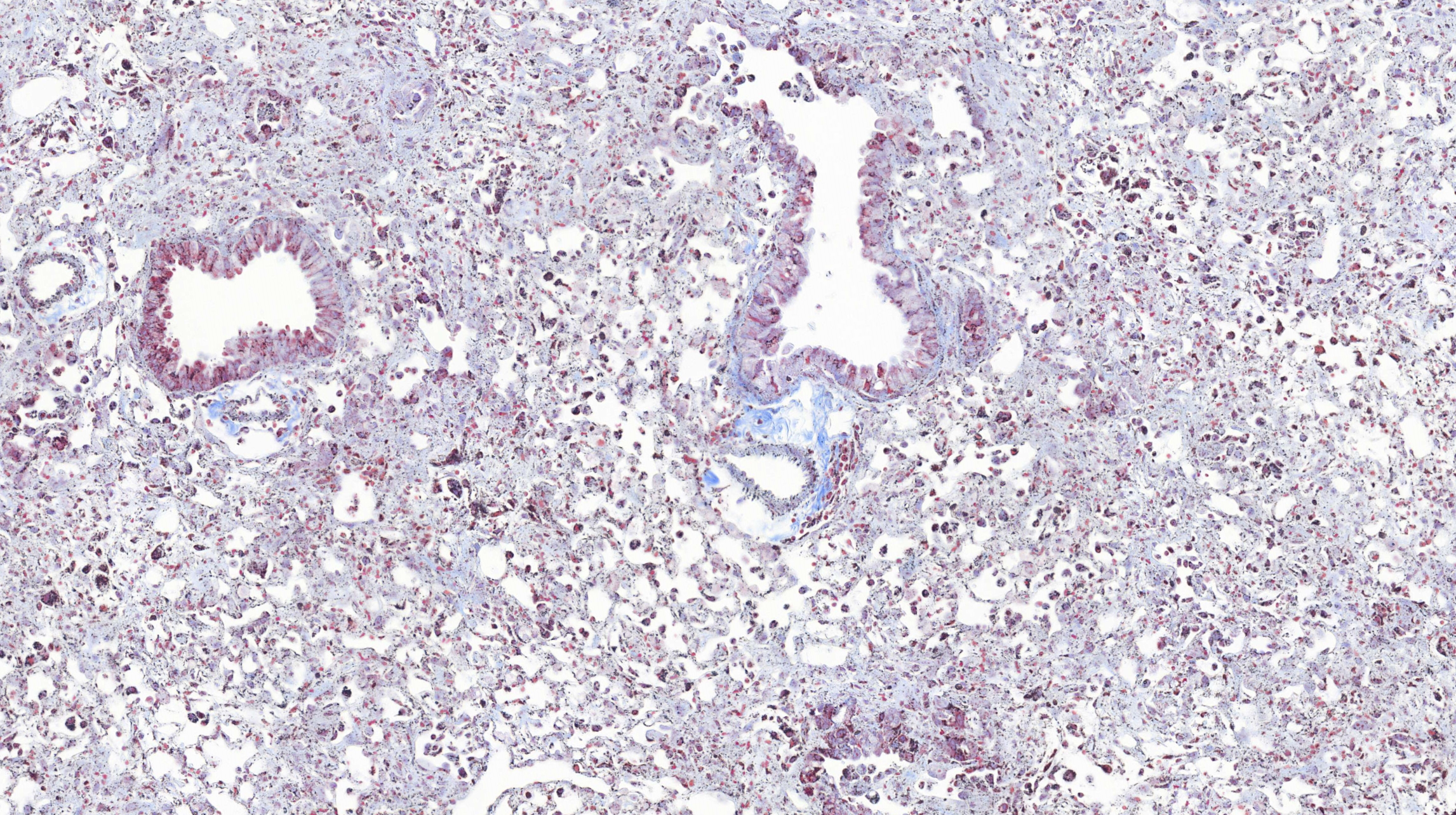

Supplement: Supplementary file 6 — Source Data for Figure 2 [file EMMM-15-e16940-s010.zip › EMM-2022-16940-Figure_1I_Source_Data/10 (BLM), trichrome staining (Aniline blue 30min) 20211227-Vehicle.jpg]

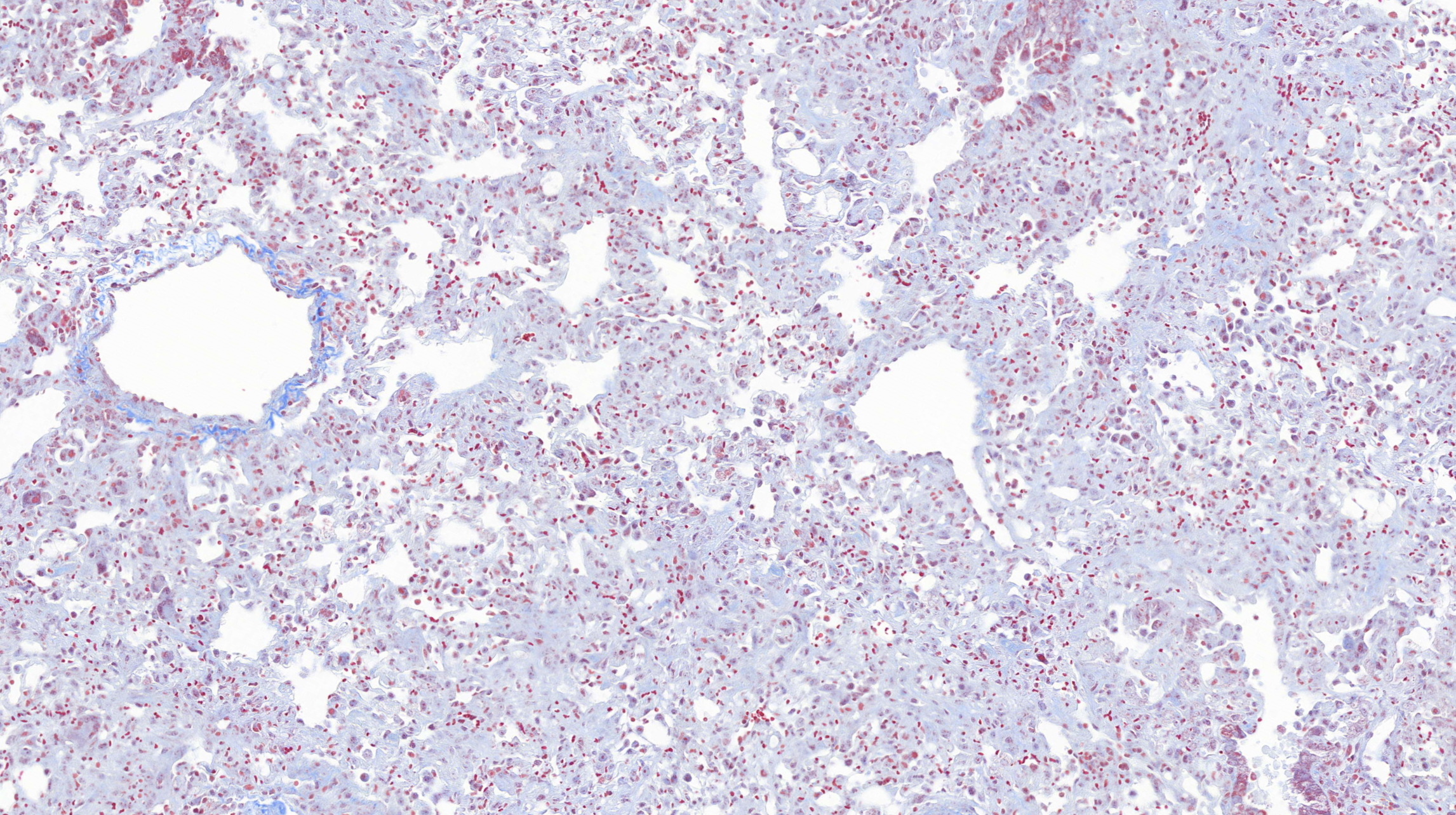

Supplement: Supplementary file 6 — Source Data for Figure 2 [file EMMM-15-e16940-s010.zip › EMM-2022-16940-Figure_1I_Source_Data/11 (HF 0.05mg'kg), trichrome staining (Aniline blue 30min) 20211227-HF 0.05.jpg]

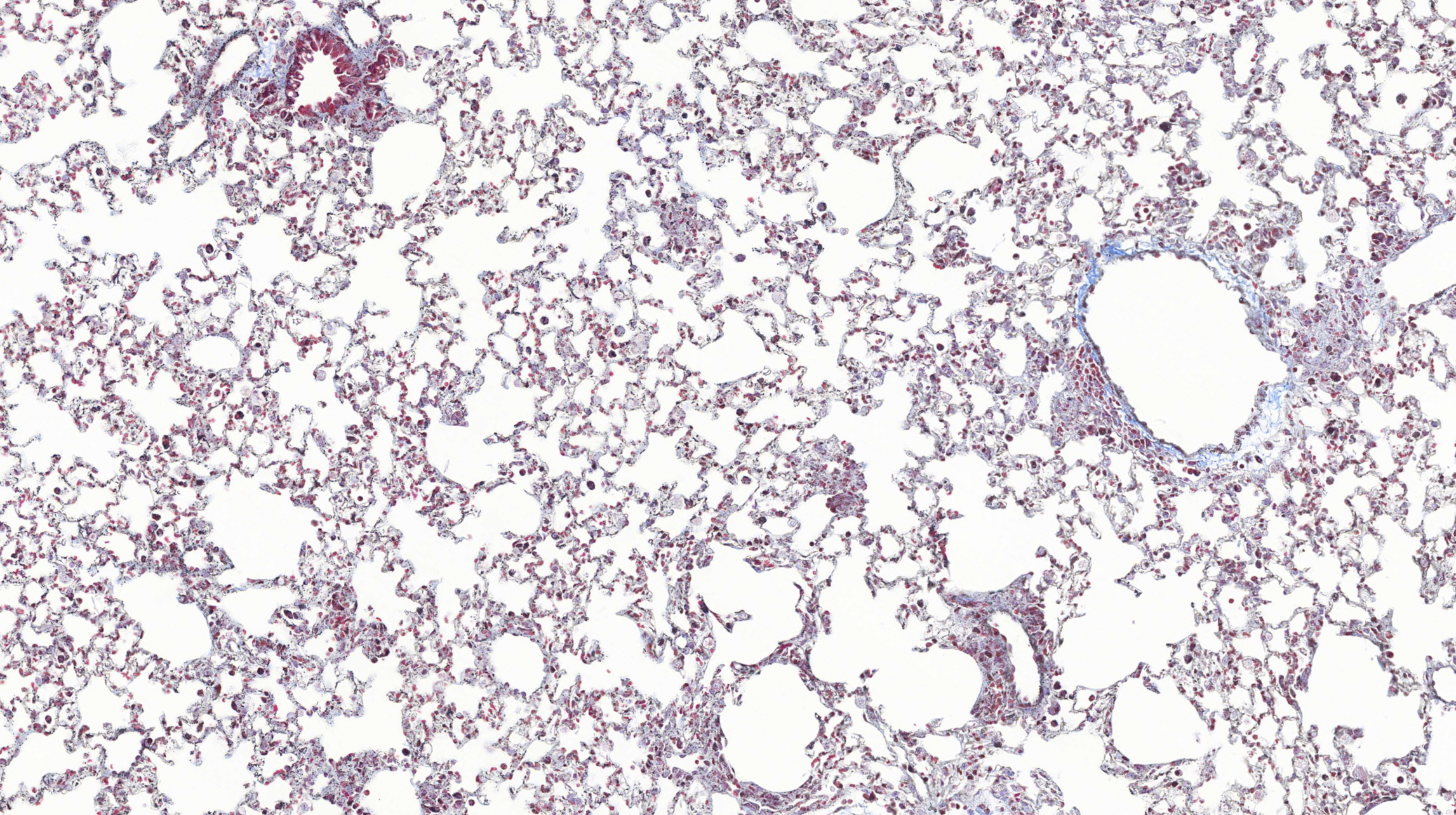

Supplement: Supplementary file 6 — Source Data for Figure 2 [file EMMM-15-e16940-s010.zip › EMM-2022-16940-Figure_1I_Source_Data/20 (HF 0.1mg'kg), trichrome staining (Aniline blue 30min) 20211227-HF 0.1.jpg]

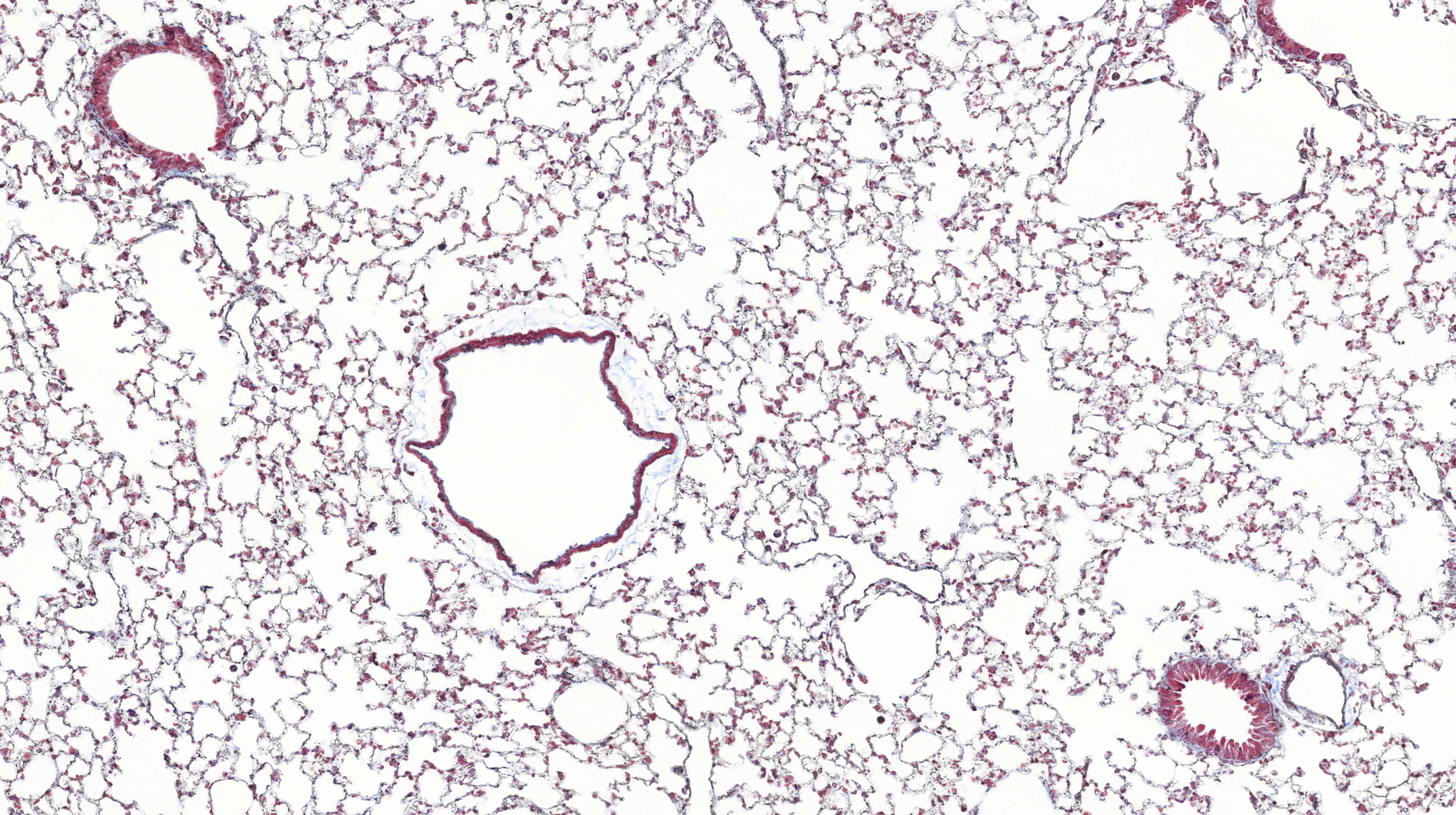

Supplement: Supplementary file 6 — Source Data for Figure 2 [file EMMM-15-e16940-s010.zip › EMM-2022-16940-Figure_1I_Source_Data/23 (DWN12088 10mg'kg), trichrome staining (Aniline blue 30min) 20211227-DWN12088 10.jpg]

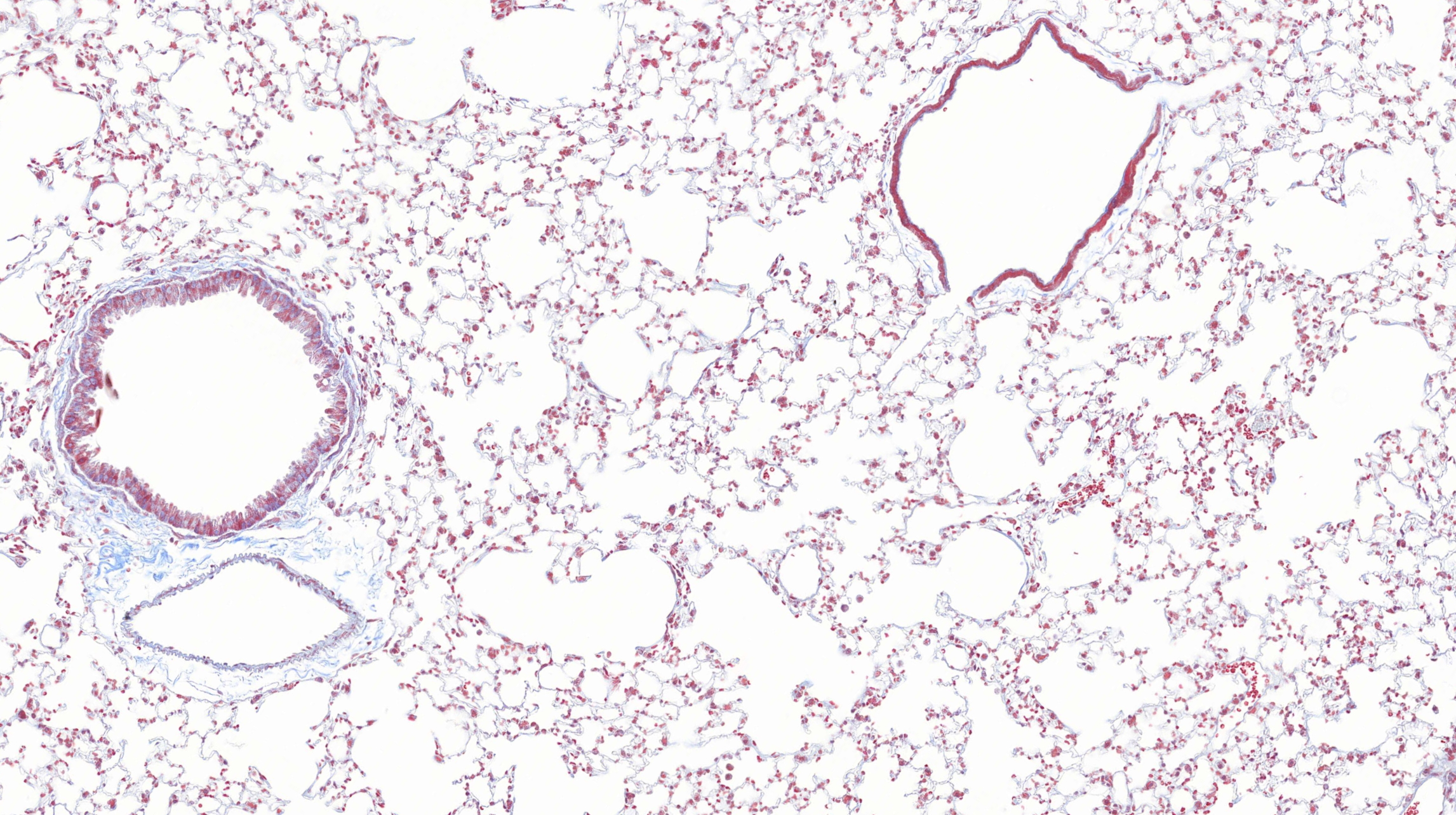

Supplement: Supplementary file 6 — Source Data for Figure 2 [file EMMM-15-e16940-s010.zip › EMM-2022-16940-Figure_1I_Source_Data/3 (NC), trichrome staining (Aniline blue 30min) 20211227-Untreated.jpg]

Figure 2A

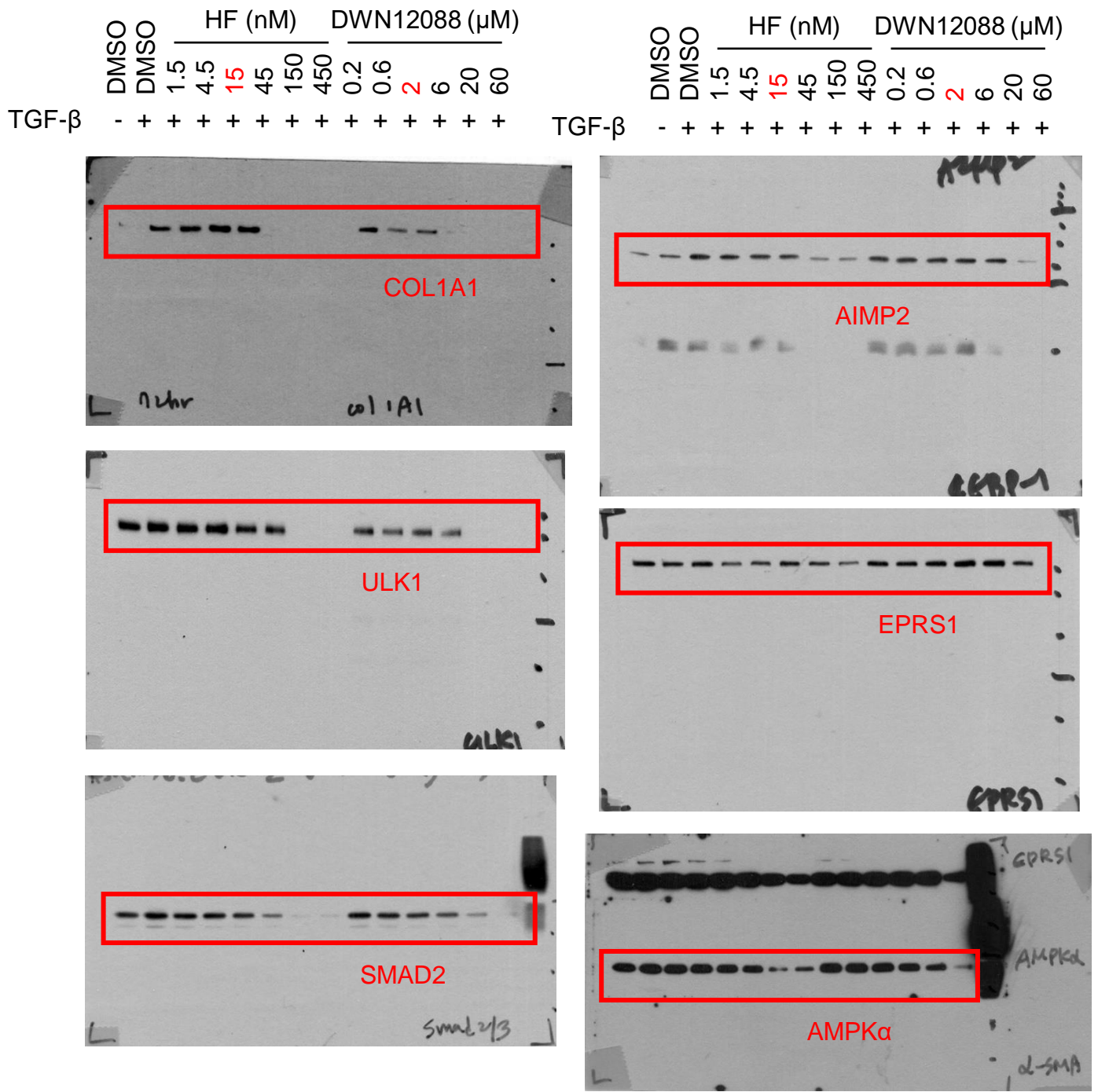

Figure 2A

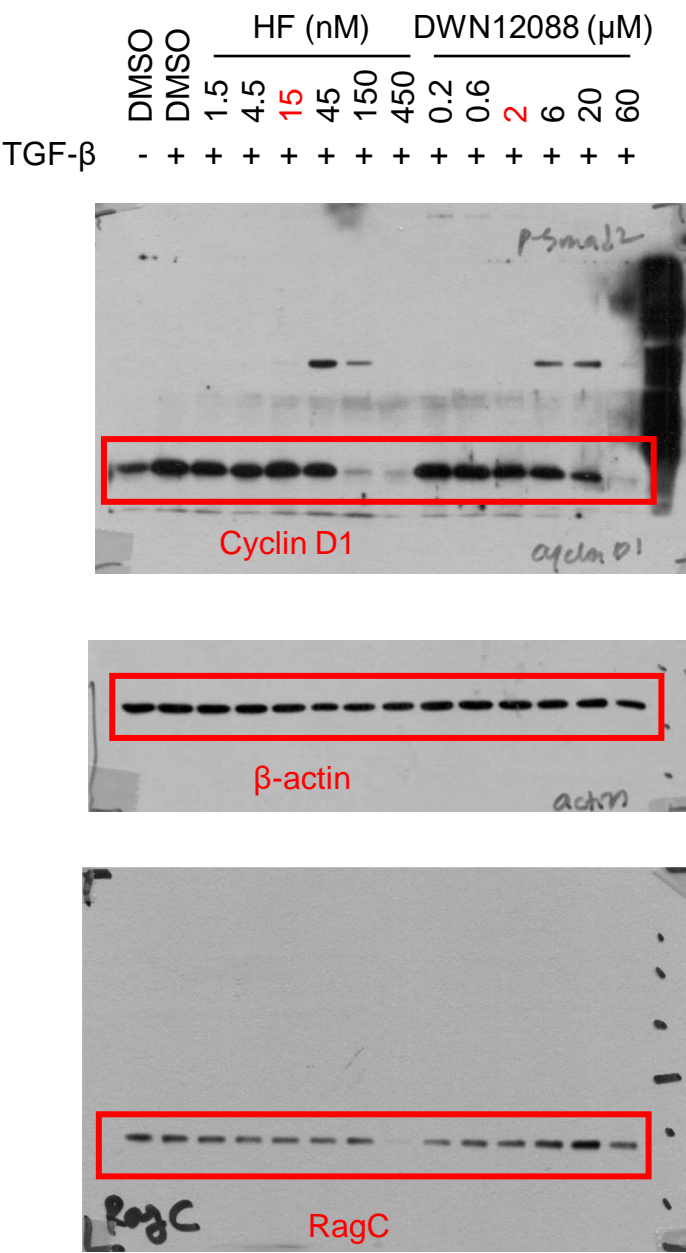

Supplement: Supplementary file 7 — Source Data for Figure 3 [file EMMM-15-e16940-s008.zip › EMM-2022-16940-Figure_2A_Source_Data.pdf]

Figure 2C

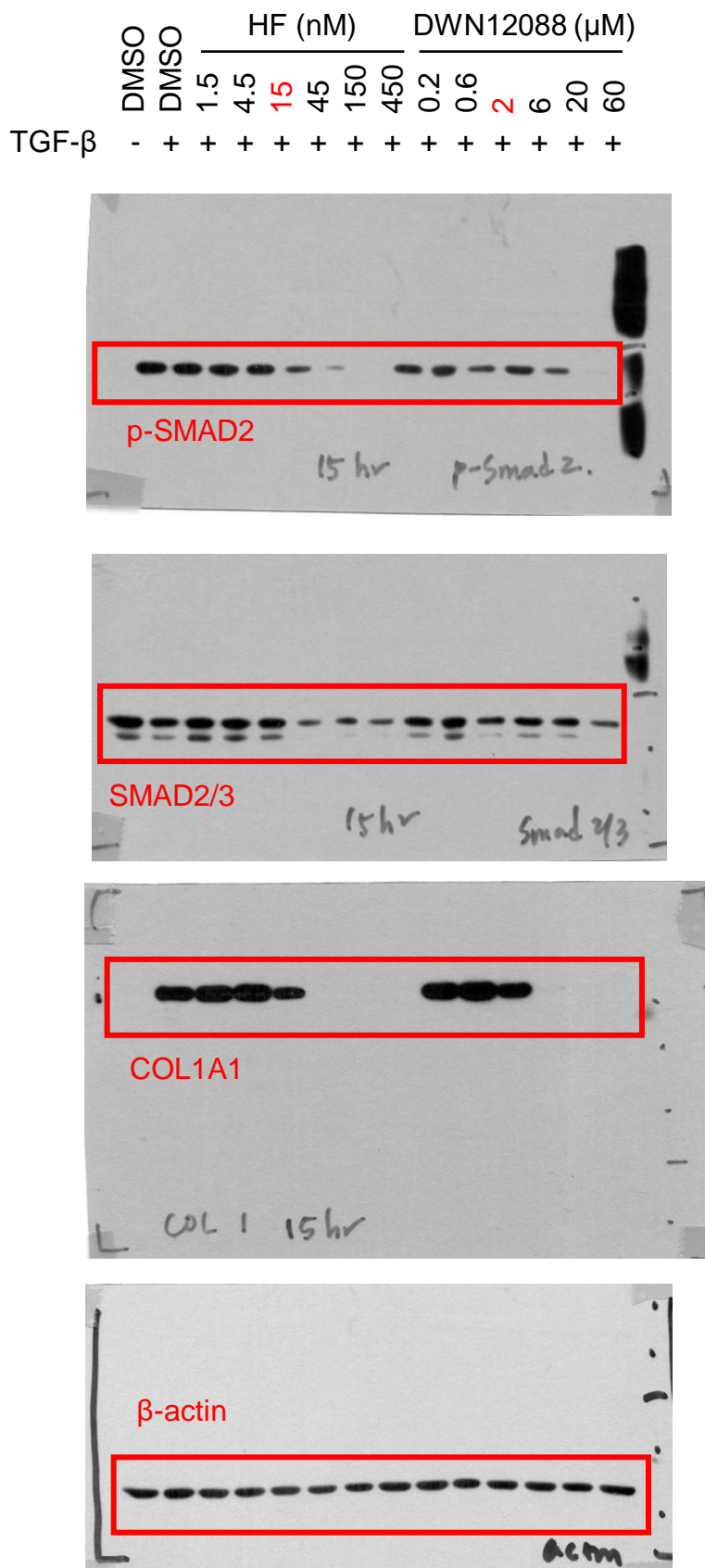

Supplement: Supplementary file 7 — Source Data for Figure 3 [file EMMM-15-e16940-s008.zip › EMM-2022-16940-Figure_2C_Source_Data.pdf]
