# Supplementary material for: Control of fibrosis with enhanced safety via asymmetric inhibition of prolyl‐tRNA synthetase 1
Source: EMBO Mol Med. 2023 May 22;15(7):e16940. doi: 10.15252/emmm.202216940 (PMC10331583; doi:10.15252/emmm.202216940)
Supplement: Supplementary file 4 — PDF+ [file EMMM-15-e16940-s004.pdf]

Article

SOURCE DATA TRANSPARENT PROCESS OPEN ACCESS

EMBO Molecular Medicine

Control of fibrosis with enhanced safety via asymmetric inhibition of prolyl-tRNA synthetase 1

Ina Yoon[1,2,†], Sulhee Kim[3,†], Minjae Cho[4], Kyung Ah You[1], Jonghyeon Son[3], Caroline Lee[4], Ji Hun Suh[1,2], Da-Jeong Bae[4], Jong Min Kim[4], Sinae Oh[1], Songhwa Park[1], Sanga Kim[1], Seong Hyeok Cho[1], Seonha Park[3], Kyuhyeon Bang[3], Minjeong Seo[3], Jong Hyun Kim[1,5], Bongyong Lee[4,‡], Joon Seok Park[4], Kwang Yeon Hwang[3,*] & Sunghoon Kim[1,2,6,**]

Abstract

Prolyl-tRNA synthetase 1 (PARS1) has attracted much interest in controlling pathologic accumulation of collagen containing high amounts of proline in fibrotic diseases. However, there are concerns about its catalytic inhibition for potential adverse effects on global protein synthesis. We developed a novel compound, DWN12088, whose safety was validated by clinical phase 1 studies, and therapeutic efficacy was shown in idiopathic pulmonary fibrosis model. Structural and kinetic analyses revealed that DWN12088 binds to catalytic site of each protomer of PARS1 dimer in an asymmetric mode with different affinity, resulting in decreased responsiveness at higher doses, thereby expanding safety window. The mutations disrupting PARS1 homodimerization restored the sensitivity to DWN12088, validating negative communication between PARS1 promoters for the DWN12088 binding. Thus, this work suggests that DWN12088, an asymmetric catalytic inhibitor of PARS1 as a novel therapeutic agent against fibrosis with enhanced safety.

Keywords collagen; drug development; fibrosis; prolyl-tRNA synthetase 1
Subject Categories Pharmacology & Drug Discovery; Respiratory System
DOI 10.15252/emmm.202216940 | Received 25 September 2022 | Revised 3 May 2023 | Accepted 3 May 2023 | Published online 22 May 2023
EMBO Mol Med (2023) 15: e16940

Introduction

Idiopathic pulmonary fibrosis (IPF) is an irreversible interstitial lung disease characterized by chronic and progressive fibrotic injuries for unknown cause. Dysregulated repair of lung injuries results in the deposition of excessive extracellular matrix, particularly collagen, leading to fibrosis with decreased lung function (Wolters *et al*, 2014; Mei *et al*, 2021). The median survival of IPF patient after diagnosis is approximately 2–4 years because there is little effective treatment for patients with end-stage disease (Ley *et al*, 2011; Richeldi *et al*, 2017). So far, two drugs, pirfenidone and nintedanib, have been approved for the management of IPF (King *et al*, 2014; Richeldi *et al*, 2014). Nevertheless, many patients are unable to take these medications due to side effects or considerations of other health statuses, implying high levels of unmet medical needs in IPF (King *et al*, 2014; Richeldi *et al*, 2014; Bando *et al*, 2016; Barratt *et al*, 2018).

Halofuginone (HF) is a halogenated derivative of fabrifuginone, an active ingredient of *Dichroa febrifuga* Lour, which was used in traditional Chinese medicine as an antimalarial agent (Coatney *et al*, 1950; Ryley & Betts, 1973). Following the finding that HF decreases the collagen content of skin, its potential applicability in anti-fibrotic therapy has been studied since abnormal function of fibrotic organs is in part attributed to pathologic accumulation of collagen (Granot *et al*, 1991, 1993; Pines & Nagler, 1998). However, studies on HF did not make a breakthrough until HF was reported to induce amino acid starvation response and its molecular target was finally identified as prolyl-tRNA synthetase 1 (PARS1) (Sundrud *et al*, 2009; Keller *et al*, 2012). After Keller *et al* (2012) proposed that HF inhibits catalytic activity of PARS1 (Keller *et al*, 2012), efforts have been made to validate the pharmacological involvement of PARS1 in diseases that were known to be controlled by HF (Katsyv *et al*, 2016; Liu *et al*, 2021). As for fibrotic diseases, the significance of PARS1 on pulmonary, hepatic, and cardiac fibrosis has been suggested so far (Song *et al*, 2018, 2019; Wu *et al*, 2020).

PARS1 is one of the aminoacyl-tRNA synthetases (ARSs), which mediate the covalent ligation of amino acids to their cognate tRNAs

1 Institute for Artificial Intelligence and Biomedical Research, Medicinal Bioconvergence Research Center, Yonsei University, Incheon, Korea
2 Yonsei Institute of Pharmaceutical Sciences, College of Pharmacy, Yonsei University, Incheon, Korea
3 Department of Biotechnology, College of Life Sciences and Biotechnology, Korea University, Seoul, Korea
4 Drug Discovery Center, Daewoong Pharmaceutical Co., Ltd, Yongin, Korea
5 Department of Biochemistry, School of Medicine, Catholic University of Daegu, Daegu, Korea
6 College of Medicine, Gangnam Severance Hospital, Yonsei University, Seoul, Korea
*Corresponding author. Tel: +82 2 3290 3009; E-mail: chahong@korea.ac.kr
**Corresponding author. Tel: +82 32 749 5401; E-mail: sunghoonkim@yonsei.ac.kr
[†]These authors contributed equally to this work
[‡]Present address: Nextgen Bioscience, Seongnam, Korea

© 2023 The Authors. Published under the terms of the CC BY 4.0 license.

for protein synthesis (Kim *et al*, 2011; Kwon *et al*, 2019). PARS1 catalyzes the covalent linkage of proline to $tRNA^{Pro}$. Unlike the other ARSs, PARS1 exists as a part of the bifunctional enzyme, glutamyl-prolyl-tRNA synthetase 1 (EPRS1), which comprises glutamyl-tRNA synthetase 1 (EARS1), three repeats of WHEP domain, and PARS1 (Kwon *et al*, 2019). Inhibiting the catalytic activity of PARS1 using HF decreases proline-rich proteins more specifically than proline-rare proteins by depriving $Pro\text{-}tRNA^{Pro}$ (Wu *et al*, 2020). Since collagen, which plays a significant role in the pathogenesis of IPF (Sgalla *et al*, 2018; Mei *et al*, 2021), contains abundant amounts of proline, the biosynthesis of collagen is thought to be highly susceptible to catalytic inhibition of PARS1.

Several clinical studies have been investigated the role of HF in various diseases, including cancer (ClinicalTrial.gov identifier: NCT00027677 and NCT00064142) and Duchenne muscular dystrophy (DMD) (ClinicalTrials.gov identifier: NCT01978366 and NCT02525302; de Jonge *et al*, 2006; Koon *et al*, 2011). In a study of cancer, most patients required 5-hydroxytryptamine ($5\text{-}HT_3$) antagonists to control side effects even at lower dose levels (de Jonge *et al*, 2006). Studies on DMD were terminated after clinical trial–related death occurred, although the Food and Drug Administration (FDA) later concluded that the company could resume the study.

Although the toxicity of HF appeared to be obvious with these clinical outcomes, the origin of its toxicity is still not well understood, and systematic studies are needed at multidimensional levels, covering from molecular and cellular assays, *in vivo* models to clinical test. In an effort to harness the therapeutic value of PARS1 catalytic inhibitors with improved tolerability, we developed a new candidate, DWN12088, and validated its safety in phase 1 studies among healthy adults (ANZCTR identifier: ACTRN12619001239156; ClinicalTrials.gov identifier: NCT04767815, NCT04888715 and NCT04888728), and recently approved by FDA to conduct phase 2 studies among IPF patients. In this paper, we investigate how DWN12088 would work more safely than HF at the molecular and cellular levels using comparative structural and kinetic analyses with HF as well as other HF derivatives in combination with their efficacy and toxicity studies.

Results

EPRS1 as a positive regulator of collagen levels

To validate the significance of EPRS1 for the regulation of collagen levels, we made WI-26 VA4 lung cells stably expressing EPRS1 only in the presence of doxycycline. The effect of EPRS1 on collagen type I alpha 1 chain (COL1A1) was monitored through the suppression of endogenous EPRS1 using siRNAs targeting the untranslated region (UTR) and then rescue of the exogenous EPRS1. Since one of the most important pathologic changes in the development of IPF is the increase of TGF-β (Roberts *et al*, 1986; Sime *et al*, 1997; Budi *et al*, 2021), we treated TGF-β to mimic the disease condition. COL1A1 protein levels were decreased by EPRS1 knockdown and increased by EPRS1 rescue (Fig EV1A). To rule out the possible involvement of nonspecific ARSs, the effects of leucyl-tRNA synthetase 1 (LARS1) and alanyl-tRNA synthetase 1 (AARS1) on COL1A1 protein levels were monitored by immunoblotting. COL1A1 protein levels showed higher dependency on the expression level of EPRS1 compared with other ARSs (Fig EV1B and C).

To see whether the higher dependency of collagen on the EPRS1 expression is related to its high content of proline, we also monitored the EPRS1 dependency of other proteins with different proline content. Since the steady-state levels of cellular proteins can be also influenced by their cellular half-lives, we also considered the potential effect of intrinsic protein half-life, and thus included cyclin D1, which has a lower proline percentage and a shorter half-life compared with COL1A1 (Fig EV1D). Whereas cellular level of COL1A1 was significantly reduced by the suppression of EPRS1, that of cyclin D1 showed no changes (Fig EV1B and C), supporting the selective effect of EPRS1 on the biosynthesis of proline-rich proteins.

Identification of novel PARS1 catalytic inhibitor, DWN12088

To identify catalytic inhibitors of PARS1 with decreased toxicity compared with HF, we synthesized 523 HF derivatives and screened them through comprehensive assays including *in vitro* prolylation (*in vitro* efficacy), collagen level in three-dimensional culture (intracellular efficacy), cytotoxicity, efficacy in transverse aortic constriction (TAC) model (*in vivo* efficacy), and 2-week repeated dose toxicity (*in vivo* toxicity) (Fig EV2A). The assays were not limited to IPF model in considering potential applications of the candidate compound to other fibrotic diseases in the future. Among the synthesized compounds, 28 compounds were selected based on their efficacy in inhibiting the catalytic activity of PARS1 (80% of the control at 1 μM and then half-maximal inhibitory concentration (IC_{50}) values less than 100 nM; Fig EV2B). We further tested their ability to decrease collagen levels (50% of the control at 1 μM and then IC_{50} values less than 400 nM) and selected eight compounds (Fig EV2C). Among them, two compounds (DWN10290 and DWN12088) were selected based on the ratio of 50% cytotoxic concentration (CC_{50}) to the IC_{50} of collagen (Fig EV2D). Both compounds showed similar efficacy in TAC model (Fig EV2E–H), whereas toxic dose of DWN12088 was higher than that of DWN10290 (Fig EV2I and Table 1). Based on these results, we finally selected DWN12088 as the candidate compound because it showed the highest therapeutic index (TI) (Fig 1A) and further applied it in an in-depth comparative analysis with HF.

Comparative analysis of HF and DWN12088 in efficacy and toxicity

We first examined the effect of proline and ATP on the PARS1 inhibitory activities of HF and DWN12088 to determine the substrate-dependent properties of DWN12088. Through *in vitro* prolylation assays, we found that DWN12088 inhibited PARS1 in proline-competitive and ATP-uncompetitive manners, similar to HF (Fig EV3A–G). Interestingly, the IC_{50} values of DWN12088, compared with those of HF, were affected more sensitively to changes in proline concentrations (Fig EV3A, B, and E). Since the physiological proline concentration is expected to be hundreds of μM (Toyoshima *et al*, 2017), the potency of DWN12088 would be more significantly affected by cellular levels of proline compared with HF. We additionally compared the effect of ATP using surface plasmon resonance (SPR). The affinity of both compounds to PARS1 was

 © 2023 The Authors

Table 1. Comparison of *in vivo* toxicities of HF and DWN12088.

	HF			DWN12088		
Dose level (mg/kg)	0.375	0.75	1.5	30	60	120 → 90*
Mortality	–	–	Found dead: 3/6 (1 animal on day 5, 2 animals on day 6) Terminal sacrifice: 3/6 (on day 6)	–	–	Terminal sacrifice: 4/6 (Each animal on days 2, 5, 6, and 12)
Clinical signs	–	Rough fur, inanimation	Rough fur, inanimation, hypothermia, shivering, incomplete eye opening	–	–	Inanimation, hypothermia
Body weight	−11% (−10% on day 5)	−20% (−10% on day 5)	NA (−14% on day 5)	–	–	NA
Hematology						
↑	–	–	NEUT	–	–	–
↓	–	–	PLT, LYMP, RET	–	HGB, MCHC	RBC, HGB, MCHC
Clinical chemistry						
↑	–	BUN, CPK	AST, ALT, ALP, BUN, CRE, IP, Ca^{2+}, Cl^{-}	–	–	AST, ALT, ALP, GLU, BUN, CRE, IP, K^{+}
↓	TG	TG	TG, TP, TCHO	–	–	TP, TCHO
Organ weight						
↑	–	–	Lung	–	–	Kidney, liver
↓	Spleen, thymus	Spleen, thymus	Spleen, thymus, testis	–	–	Spleen, thymus
Necropsy finding	• Pale liver (5/6)	• Pale liver (5/6) • Thymus atrophy (1/6) • Damaged stomach mucosa (2/6) • Dark red contents in stomach (2/6)	• Pale liver (5/6) • Dark red contents in stomach (2/6) • Testicular congestion (1/6)	–	–	• Pale liver (1/6) • Thymus atrophy (3/6) • Pale and large kidney (1/6) • Dark red contents in the stomach (1/6)
NOAEL	Below 0.375 mg/kg			60 mg/kg		

ICR mice were orally administered with the indicated amount of HF and DWN12088 once daily for 2 weeks. Clinical signs, body weight, hematology results, clinical chemistry results, organ weight, and necropsy findings were observed as described in the Materials and Methods. ALP, alkaline phosphatase; ALT, alanine aminotransferase; AST, aspartate aminotransferase; BUN, blood urea nitrogen; CRE, creatinine; GLU, glucose; HGB, hemoglobin; IP, inorganic phosphorus; LYMP, lymphocyte; MCHC, mean corpuscular hemoglobin concentration; NEUT, neutrophil; NOAEL, no observed adverse effect level; PLT, platelet; RBC, red blood cell; RETI, reticulocyte; TCHO, total cholesterol; TG, triglyceride; TP, total protein.
*The dose was changed from 120 mg/kg to 90 mg/kg from day 6.

significantly increased in the presence of ATP, further validating the positive role of ATP in the binding of the compounds to PARS1 (Fig EV3H).

We compared the efficacy of HF and DWN12088 in decreasing collagen levels via enzyme-linked immunosorbent assay (ELISA) detecting the level of pro-collagen 1 in WI-26 VA4 human lung cells. HF and DWN12088 showed IC_{50} values of 15.19 and 2,025 nM, respectively (Fig 1B), indicating that DWN12088 is about 133-fold less potent than HF in the inhibition of collagen biosynthesis. The CC_{50} values of HF and DWN12088 were determined as 52.55 and 20,847 nM (~ 397 fold), respectively (Fig 1B). Based on the obtained dose–response curves, we calculated TI by dividing the CC_{50} values by the IC_{50} values for collagen levels, because pharmacological safety is determined by TI (Fig 1C). The TI of DWN12088 ($TI_{DWN12088}$ = 10.29) was about three times wider than that of HF (TI_{HF} = 3.46). Taken together, DWN12088 shows the higher safety in cell compared with HF with the lower potency in the inhibition of collagen synthesis.

To investigate *in vivo* efficacy of DWN12088, we first monitored the dose-dependent efficacy and plasma concentrations of DWN12088 to determine the optimal dosage. In the efficacy test of DWN12088 using a preventive model of bleomycin (BLM)-induced lung fibrosis, in which the compounds were administered a day before BLM administration, DWN12088 alleviated fibrotic properties in mice treated with 10 and 30 mg/kg but not in mice treated with 3 mg/kg (Fig EV4A–C). DWN12088 decreased fibrotic properties to similar extent at both 10 and 30 mg/kg (Fig EV4A–C) while plasma concentrations of DWN12088 were higher at 30 mg/kg (Appendix Fig S1A–D), suggesting the potential saturation of the DWN12088 efficacy at 10 mg/kg. DWN12088 at 10 mg/kg showed the efficacy comparable with nintedanib and pirfenidone in a therapeutic BLM model, in which the compounds were administered a week after BLM administration (Fig EV4D–F) (Carrington *et al*, 2018). Based on these results, we determined 10 mg/kg as the optimal dosage of DWN12088.

We compared the *in vivo* efficacy of HF and DWN12088 in two different therapeutic BLM models (Fig 1D). These models differ in the timing of compound administration after BLM challenge. In both models, we monitored peripheral oxygen saturation (SpO_2) as a measurement for pulmonary function, total cell counts in

© 2023 The Authors

bronchoalveolar lavage fluid (BALF), and collagen content. Although mice treated with 0.05 mg/kg of HF did not show an improvement in SpO_2, mice with HF 0.1 mg/kg and DWN12088 10 mg/kg showed a similar recovery in SpO_2 (Figs 1E and EV4G). Mice treated with HF 0.1 mg/kg and DWN12088 10 mg/kg also showed a similar decrease in total cell count in BALF (Figs 1F and EV4H) and collagen content in the right lung (Figs 1G and EV4I). Masson's trichrome staining images also revealed a similar decrease in collagen levels between the groups. Based on the images, we observed that bleomycin administration increased collagen accumulation in interstitial spaces, bronchioles, and blood vessels. Administration of HF and DWN12088 decreased collagen deposition in the interstitial spaces, which resulted in the restoration of alveolar structures (Fig 1H and I; Appendix Fig S2). All these results implied that the *in vivo* effective dose of DWN12088 would be about 100 times higher than that of HF, which was consistent with the results shown in the cell-based assays (Fig 1B).

Next, we compared the *in vivo* toxicity of HF and DWN12088 through a 2-week repeated-dose toxicity study (Table 1). HF was administered to mice at concentrations of 0.375, 0.75, and 1.5 mg/kg.

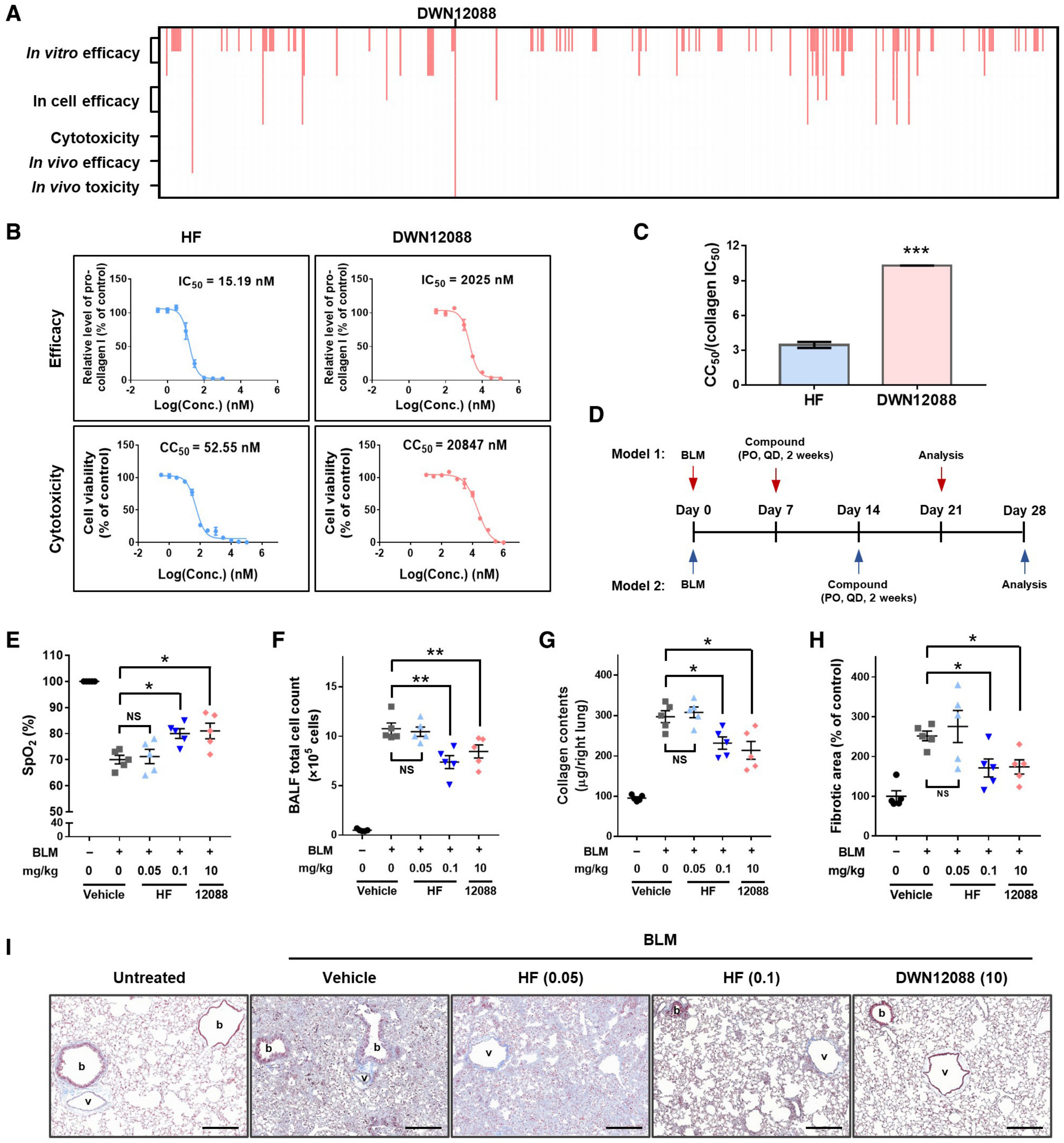

Figure 1.

 © 2023 The Authors

Figure 1. Identification and characterization of DWN12088 with higher safety compared with HF.

A A total 523 HF derivatives were tested for *in vitro* efficacy, intracellular efficacy, cytotoxicity, *in vivo* efficacy, and *in vivo* toxicity as described in the Methods section. DWN12088 was selected as the final candidate. At each step, the compounds that met each screening criteria are shown in pink lines. The detailed screening strategy is described in Fig EV2.

B Dose–response curve of HF and DWN12088 in WI-26 VA4 cells. The IC_{50} value for collagen level was measured as a representative of chemical efficacy. The cells were starved of serum for 6 h and then incubated with the indicated concentrations of compounds in the presence of TGF-β (2 ng/ml) for 72 h. The secreted pro-collagen I levels were determined by ELISA. The CC_{50} values were determined by CellTiter-Glo assay. Dose–response curves were determined by combining at least two biologically independent experiments. The IC_{50} values were calculated using GraphPad Prism 7.0 (Collagen level, $n = 5$ from three independent experiments (singlicate for one experiment and duplicate for two experiments; mean ± SEM); Celltiter-Glo assay, $n = 4$ from two independent experiments (duplicate for each experiment; mean ± SEM).

C The TI of HF and DWN12088 was determined by dividing the CC_{50} with the IC_{50} for collagen level (Welch's t test; ***$P < 0.001$; mean ± SEM).

D Schedules of two different *in vivo* efficacy tests.

E–I *In vivo* efficacy of HF and DWN12088 was determined in a bleomycin-induced lung fibrosis model. The indicated concentrations of HF or DWN12088 were orally administered to mice once a day for 2 weeks from a week after intratracheal injection of bleomycin (D, model 1). The saturation of percutaneous oxygen (SpO_2) (E) was determined as a measurement of lung function. The number of cells in bronchoalveolar lavage fluid (BALF) was counted as a measure of inflammation response (F). The collagen level was determined by hydroxyproline assay (G) and Masson's trichrome staining (H and I) of lung tissues. The remaining Masson's trichrome staining images shown in Fig 1I are presented in Appendix Fig S2 ($n = 5$; Mann–Whitney test after Kruskal–Wallis test; *$P < 0.05$, **$P < 0.01$; mean ± SEM). scale bar = 200 μm. BLM, bleomycin; PO, per oral; QD, once a day; 12088, DWN12088; HF (0.05), HF 0.05 mg/kg; HF (0.1), HF 0.1 mg/kg; DWN12088 (10), DWN12088 10 mg/kg; b, bronchiole; v, blood vessel.

Source data are available online for this figure.

In the group treated with HF at a dose of 1.5 mg/kg, one mouse was found dead on day 5, two mice died on day 6, and we euthanized remaining three mice on day 6 due to general poor health. On the other hand, in the group treated with DWN12088 at doses of 30, 60, and 120 mg/kg, we euthanized three mice treated with 120 mg/kg due to poor health conditions on day 2, 5, and 6. We then treated the remaining three mice at a reduced dose of 90 mg/kg from day 6. Among them, one mouse was euthanized on day 12, and the remaining two mice survived to the end of the study.

The HF-treated group showed a > 10% reduction in body weight at all the tested doses, whereas DWN12088-treated group did not show weight loss. In the hematologic analysis, there were few significant changes in the toxicological profile of both the HF and DWN12088 groups. In the clinical chemistry analysis, the levels of liver (aspartate aminotransferase (AST), alanine aminotransferase (ALT), alkaline phosphatase (ALP))- and kidney (blood urea nitrogen (BUN), creatinine (CRE), electrolytes)-related factors were increased at the 1.5 mg/kg of HF and 120 mg/kg of DWN12088 groups. The weights of the spleen and thymus were decreased in the HF-treated groups at all doses and in the DWN12088-treated group at 120 mg/kg. At necropsy, the HF-treated groups at all tested doses and the DWN12088-treated group at 120 mg/kg showed pale livers. In addition, dark red contents in the stomach suspected to be due to bleeding were found more frequently in the HF-treated groups. Determination of the no observed adverse effect level (NOAEL) of HF was difficult because HF-treated mice showed a > 10% weight loss even in the lowest dose. We just presumed that the NOAEL of HF might be lower than 0.375 mg/kg. On the other hand, the results obviously suggested the NOAEL of DWN12088 to be 60 mg/kg. Thus, the NOAEL of DWN12088 was more than 160 times higher than that of HF, implying that DWN12088 is a safer compound than HF *in vivo*.

Effect of HF and DWN12088 on global translation and the TGF-β pathway

To understand the reason for the TI differences between HF and DWN12088, we compared the effect of HF and DWN12088 on known functions of HF such as inhibition of global translation and TGF-β pathway. When comparing dose-dependent effect of the compounds on the intracellular levels of some selected cellular proteins with different proline contents, the tested proteins showed the sensitivity to the compounds in proportion to their proline contents (Fig 2A and B), suggesting again that PARS1 would function as a suitable target to specifically control proline-rich proteins such as collagen with little or lower influence on general protein synthesis. In comparing the dose-dependent protein suppression patterns of HF and DWN12088, we found that proteins with lower proline proportions, such as AMPKα and cyclin D1, were three times more sensitive to HF, whereas proteins with higher proline proportions, such as COL1A1 and ULK1, showed similar patterns (Fig 2A). To observe the general trend, a plot of "proline percentage" versus "relative chemical concentration required for complete suppression of protein" was produced (Fig 2B). The trend lines showed a negative association between the two factors, suggesting proline content-dependent control of protein levels, and the absolute value of the slope was larger in DWN12088, suggesting that, compared with HF, a higher concentration of DWN12088 should be required to suppress biosynthesis of proteins with low proline content.

Since HF was previously shown to decrease the phosphorylation of SMAD2/3 (Zion *et al*, 2009; Roffe *et al*, 2010; Assis *et al*, 2015), we also monitored the effects of HF and DWN12088 on the TGF-β pathway. Although both of HF and DWN12088 reduced SMAD2 phosphorylation, the concentrations required for the inhibition of SMAD2 phosphorylation were significantly higher than those required for the inhibition of collagen synthesis (Fig 2A and C). In addition, HF and DWN12088 showed similar dose-dependent suppression patterns of SMAD2 phosphorylation (Fig 2D). Thus, their effects on TGF-β signaling did not appear to be primarily responsible for the inhibition of collagen biosynthesis, and the TI difference between the compounds might result from their effects on global translation rather than TGF-β pathway.

Comparison of binding modes of the compounds

To understand the detailed differences between HF and DWN12088 at molecular level, we determined co-crystal structures of PARS1 complexed with DWN12088 and three additional DWN compounds

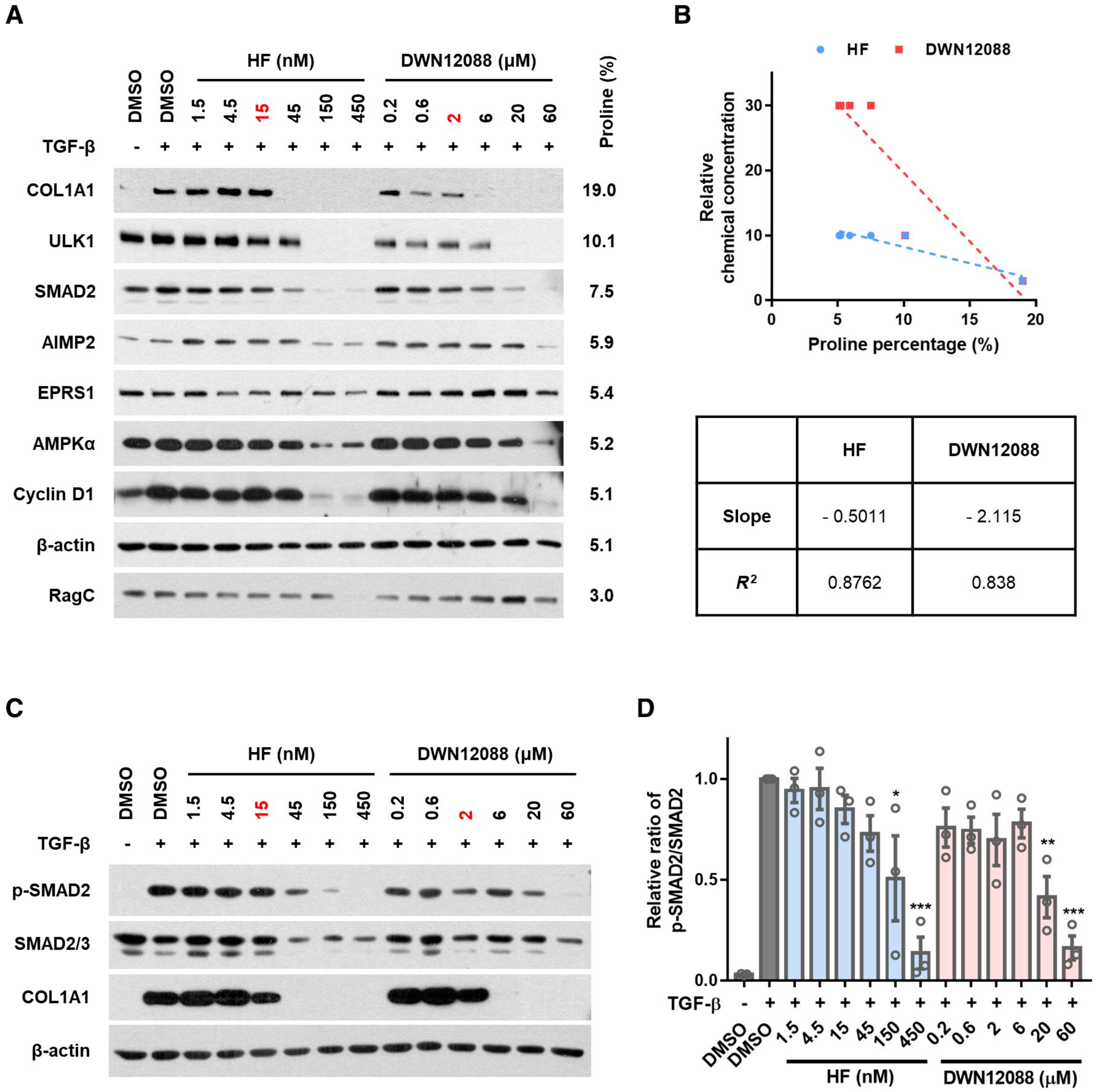

Figure 2. Effect of HF and DWN12088 on protein translation and TGF-β pathway.

A WI-26 VA4 cells were incubated in the presence of the indicated concentrations of HF and DWN12088 for 72 h. The chemical concentrations were determined based on the IC_{50} for collagen level (HF, 15 nM; DWN12088, 2 μM). The levels of proteins were determined by immunoblot assay. The proline content of the proteins based on Uniprot amino acid sequence is shown on the right side of the immunoblot images.

B The plot of "proline percentage in the protein" versus "chemical concentration relative to IC_{50} for collagen" is displayed based on (A). The lowest concentration at which protein levels started to decrease was normalized by IC_{50} values for collagen level and used as the y-axis value. Proteins with no change in level, such as RagC in the DWN12088-treated group, were excluded from the plot. The slope and R^2 value were calculated using GraphPad Prism 7.0. ULK1, unc-51 like autophagy activating kinase 1; AIMP2, aminoacyl tRNA synthetase complex interacting multifunctional protein 2; AMPKα, protein kinase AMP-activated catalytic subunit alpha; RagC, Ras-related GTP binding C.

C WI-26 VA4 cells were incubated with the indicated concentrations of HF and DWN12088 in the presence of 2 ng/ml TGF-β for 15 h. The chemical concentrations were determined based on the IC_{50} values for collagen levels (HF, 15 nM; DWN12088, 2 μM). The activation of the TGF-β pathway was determined by monitoring the phosphorylation of SMAD2. Immunoblot images are representative of three biologically independent experiments.

D The band intensities of the immunoblot images in (C) were quantified using ImageJ ($n = 3$; One-way ANOVA; *$P < 0.05$, **$P < 0.01$, ***$P < 0.001$; mean ± SEM).

Source data are available online for this figure.

along with ATP (Table 2) and compared their structures with the PARS1-HF-ANP (phosphoaminophosphonic acid-adenylate ester) complex structure (PDB: 4HVC) (Zhou *et al*, 2013). Overall, DWN compounds commonly bound to the catalytic site in a similar mode as HF (Figs 3A–C and EV5A–D). HF and DWN compounds showed similar chemical structures consisting of a piperidine ring, halogenated bicyclic ring, and 3-carbon bridge. The bicyclic ring of DWN compounds is located in the tRNA-binding site via π-stacking interaction with F1097 and via the interaction of halogen atoms with H1093 (Fig EV5D). The piperidine ring moiety of DWN compounds

 © 2023 The Authors

Table 2. X-ray diffraction data collection and refinement statistics.

	DWN11251 (7Y28)	DWN11748 (7Y1H)	DWN11761 (7Y3S)	DWN12088 (7Y1W)
Structural characteristics				
Wavelength (Å)	1.0000	1.0000	1.0000	1.0000
Space group	P 2_1	P 2_1	P 2_1	P 2_1
Cell dimensions				
a, b, c (Å)	71.6 92.4 86.1	71.7 92.4 87.5	71.9 92.0 87.3	71.6 92.1 86.7
β (°)	108.7	108.2	108.2	108.8
Resolution (Å)	50.00–2.29 (2.33–2.29)	50.00–1.99 (2.02–1.99)	50.00–2.60 (2.64–2.60)	50.00–2.50 (2.54–2.50)
R_{merge}	0.047 (0.296)	0.052 (0.330)	0.080 (0.502)	0.052 (0.483)
$I/I\sigma I$	22.8 (2.47)	23.3 (2.63)	13.7 (2.74)	22.3 (2.57)
Completeness (%)	93.8 (68.0)	89.1 (63.2)	98.1 (94.9)	88.4 (74.2)
Redundancy	4.5 (2.2)	4.1 (2.0)	4.7 (3.1)	2.7 (1.6)
Refinement characteristics				
Resolution (Å)	45.43–2.29	37.90–1.99	41.25–2.60	33.22–2.50
No. reflections	45,110	65,979	32,739	32,528
R_{work}/R_{free}	0.21 / 0.26	0.21 / 0.24	0.20 / 0.25	0.23 / 0.29
No. atoms				
Protein	7,626	7,698	7,742	7,748
Ligand/ion	122	112	111	77
Water	132	201	67	37
Ramachandran plot (%)				
Favored regions	98	99	99	98
Allowed regions	2	1	1	2
Outlier regions	0	0	0	0
B-factors				
Protein	52.1	46.5	44.3	76.9
Ligand/ion	50.8	42.1	41.1	71.6
Water	46.8	42.2	39.2	66.5
R.m.s deviations				
Bond length (Å)	0.011	0.007	0.004	0.008
Bond angles (°)	1.406	1.097	0.934	1.405

Structural and refinement characteristics of human PARS1 complexed with the indicated DWN compounds are listed. Values in parentheses are for the highest resolution shell.

occupies a proline-binding site and a ketone group (DWN11251, DWN11748, and DWN11761) or alkane group (DWN12088) connects the piperidine ring with the bicyclic ring, similar to HF (Son *et al*, 2013). We defined the PARS1 residues K1091–F1097 (interacting with the bicyclic ring of the compounds) as the R-loop and A1152– R1165 (interacting with ATP) as the A-loop (Fig 3A and B).

PARS1 is a homodimer formed by the PARS1 protomers A and B (Zhou *et al*, 2013). Interestingly, when the protomers were superimposed, the compounds were classified into two groups according to binding modes. DWN12088 and DWN11251 bound to PARS1 protomers asymmetrically (compounds with asymmetric binding, ABC), whereas HF, DWN11748, and DWN11761 bound symmetrically (compounds with symmetric binding, SBC) (Fig 3C). More specifically, protomers A and B of SBC-bound PARS1 and protomer A of ABC-bound PARS1 showed similar structures to each other, whereas protomer B of ABC-bound PARS1 was different from the others in terms of the binding of both ATP and the compounds (Fig 4A and B).

The most striking difference between the structures was the loss of the electron density map of ATP in protomer B of the DWN12088-bound structure (Fig 4A). The electron density map of ATP in protomer B of the DWN11251-bound structure was also unclear compared with the others (Fig 4A). To presume the binding affinity of ATP to PARS1, we observed hydrogen bond networks of ATP with PARS1 and the compounds (Fig 4B). In protomers A and B of SBC-bound PARS1, and protomer A of ABC-bound PARS1, the phosphate group of the ATP formed multiple hydrogen bonds with the A-loop via water molecules and a magnesium ion. Particularly, R1152 formed hydrogen bonds with the ketone group of the compounds (except DWN12088), α-phosphate of ATP, and water molecules. E1154 and R1163 also formed direct hydrogen bonds with the γ-phosphate of ATP and indirect hydrogen bond networks via water

© 2023 The Authors

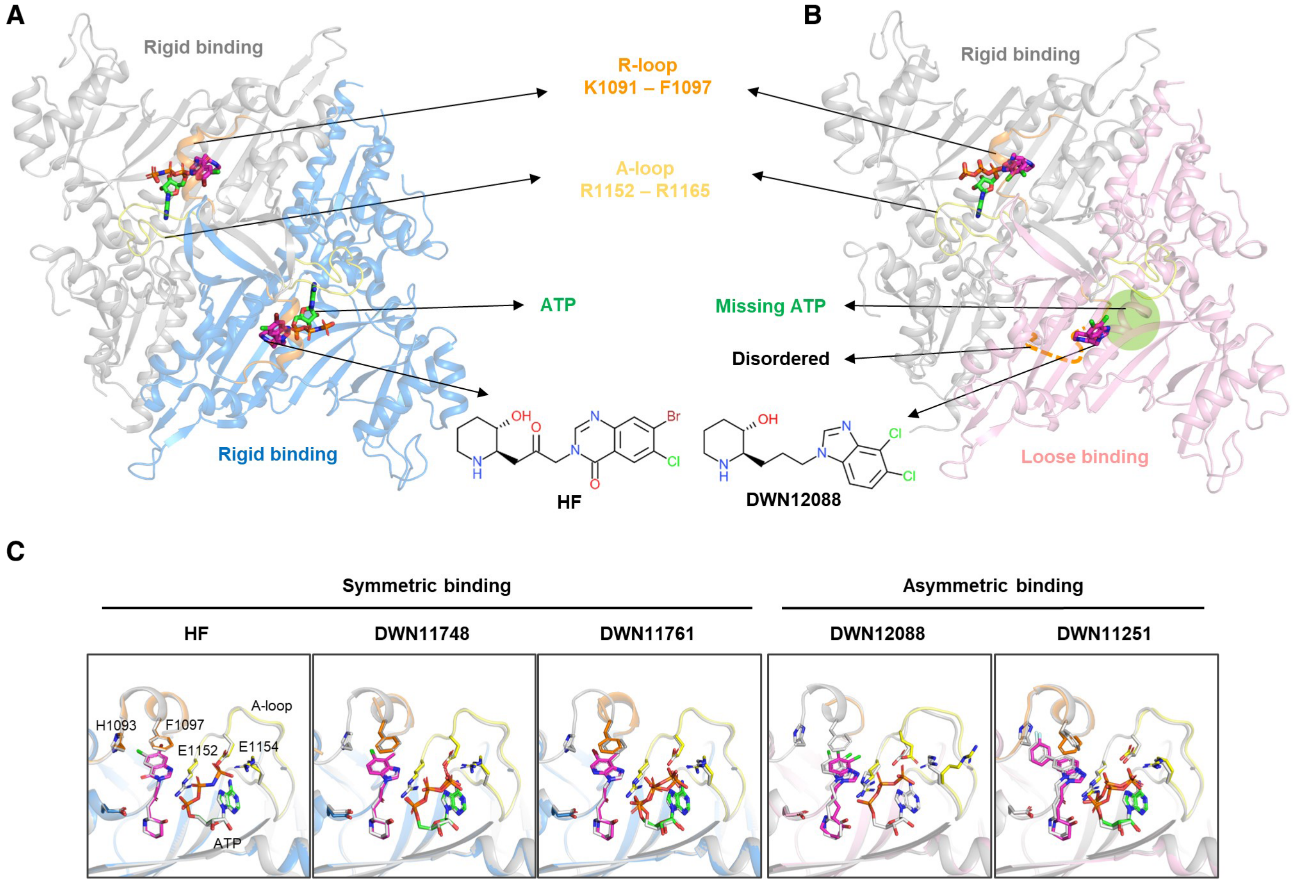

Figure 3. Comparison of HF and DWN compounds in the PARS1 binding mode.

A, B Overall structures of PARS1 complexed with the indicated compounds. PARS1 shows two types of the compound-binding states (rigid and loose binding). PARS1 chains with rigid binding are displayed in gray and blue, whereas those with loose binding are displayed in pink. ATP is designated as green, and the compounds are designated as magenta stick models. R-loop (K1091 – F1097) and A-loop (R1152 – R1165) are presented as orange and yellow, respectively. The disordered region of R-loop is presented as orange dashed lines.

C Superposition of PARS1 protomers A and B of DWN compound- and HF-bound structures at the residues important for the interaction with the compounds and ATP. Structures of protomer A are represented as transparent gray stick models. The color code of the structures is the same as that shown in Fig 3A and B.

Source data are available online for this figure.

molecules. However, in protomer B complexed with DWN12088, there was no hydrogen bond due to the absence of an ATP molecule. Protomer B of the DWN11251-bound structure also showed the decreased number of hydrogen bonds compared with protomer A. When comparing the number of hydrogen bonds of protomers A and B with each compound, relatively similar numbers of hydrogen bonds were counted in the complexes with SBC. In contrast, protomer A made over 10 more hydrogen bonds than protomer B in the complexes with ABC, implying the flexible or weakened binding of ATP in PARS1 protomer B (Fig 4B). Considering that the binding of the compounds to PARS1 is increased in the presence of ATP (Fig EV3H), differences in ATP binding affinity would affect compound binding.

Asymmetric binding of DWN12088 and DWN11251 to PARS1 dimer

Other than ATP binding to PARS1, we also observed the different binding modes of the compounds themselves. ABC in protomer B exhibited a less clear electron density map compared with SBC in protomers A and B and ABC in protomer A (Fig 4A). The different compound-binding modes in protomer B of ABC-bound PARS1 structures may result from different reasons. The flexibility of the bridge between bicyclic and piperidine rings could be the reason for DWN12088. While the ketone group of the SBC formed stable hydrogen bonds with ATP, contributing to their tight binding to PARS1, DWN12088 did not have a ketone group in the bridge, which could decrease the rigidity of bicyclic ring orientation (Fig 4B). In fact, the electron density of the bicyclic ring moiety of DWN12088 in protomer B of the complex structure was not well defined, and the R-loop interacting with the bicyclic ring moiety was almost disordered (Fig 4A), implying a potential loose binding of DWN12088 in PARS1 protomer B.

Another ABC, DWN11251, had a ketone group in the bridge, unlike DWN12088, and the halogen substituent of the bicyclic ring in HF was changed to a halogenated benzyl group, making the structure bulkier (Fig EV5C). All the compounds stabilized the R-loop through the interaction between the halogen atom and H1093

 © 2023 The Authors

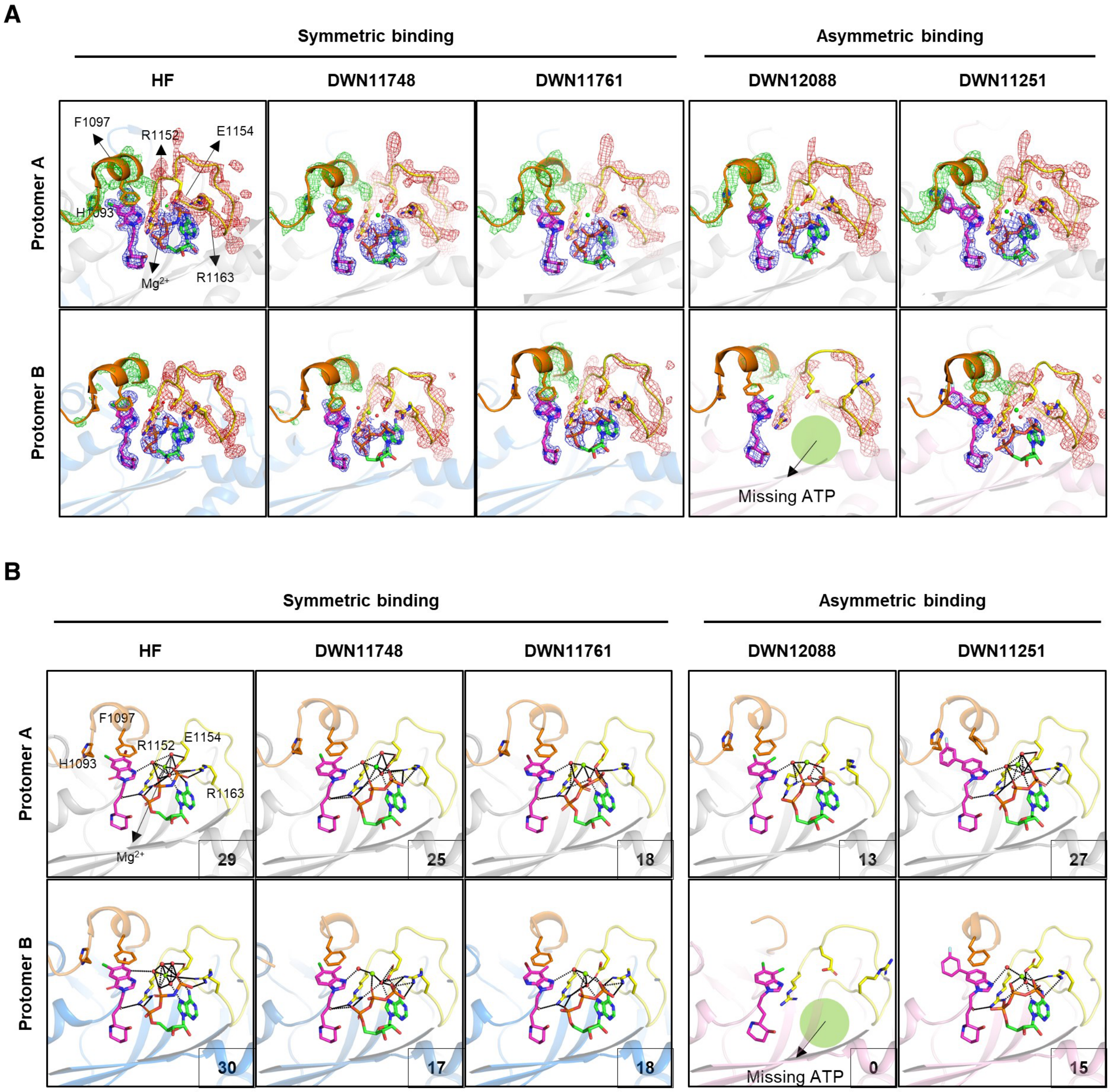

Figure 4. Detailed PARS1 binding modes of HF and DWN compounds.

A Zoomed view of the compound binding site and its surrounding. The 2Fo − Fc map of compounds and the loop are shown as mesh model at a 2.0-sigma (blue mesh) and 1.5-sigma contour level (green mesh, R-loop; brown mesh, A-loop), respectively.

B Detailed binding mode of HF and DWN compounds. The color scheme of PARS1 is identical to that used in Fig 3A and B. Water molecules and Mg^{2+} ion are represented as red and green spheres, respectively. The residues that interact with ATP and compounds are represented as stick models. Hydrogen bond network is represented as black dotted lines. The number of hydrogen bonds between compounds or ATP and the residues of the A-loop is shown at the bottom right side of the image.

(Lu *et al*, 2010; Jiang *et al*, 2016). In protomer A, halogen atoms in HF and DWN12088 showed an optimal distance to H1093 of 3.36–4.26 Å, and the distance between H1093 and the fluoride group in DWN11251 was 3.22 Å (Appendix Fig S3A and B). Considering that the van der Waals radius of the fluoride (147 pm) is smaller than that of chloride (175 pm) or bromide (185 pm), this distance seems to be suitable for each halogen atom to form a halogen interaction with H1093. On the other hand, since the distance was very close (2.2 Å) in DWN11251-bound protomer B, the R-loop was not stabilized due to the van der Waals repulsion or steric hindrance with halogen atom (Appendix Fig S3C), possibly resulting in the unstable binding of DWN11251 to the R-loop in protomer B. Overall, the flexibility of the A-loop and R-loop in the PARS1-compound complex structures seemed to be responsible for the different binding modes between ABC and SBC.

Effect of asymmetric binding of DWN12088 on the dose–response curve

Since both DWN12088 and DWN11251 appeared to bind to the two promoters of PARS1 in an asymmetric mode, we suspected that the catalytic activity of each PARS1 protomer would respond differently

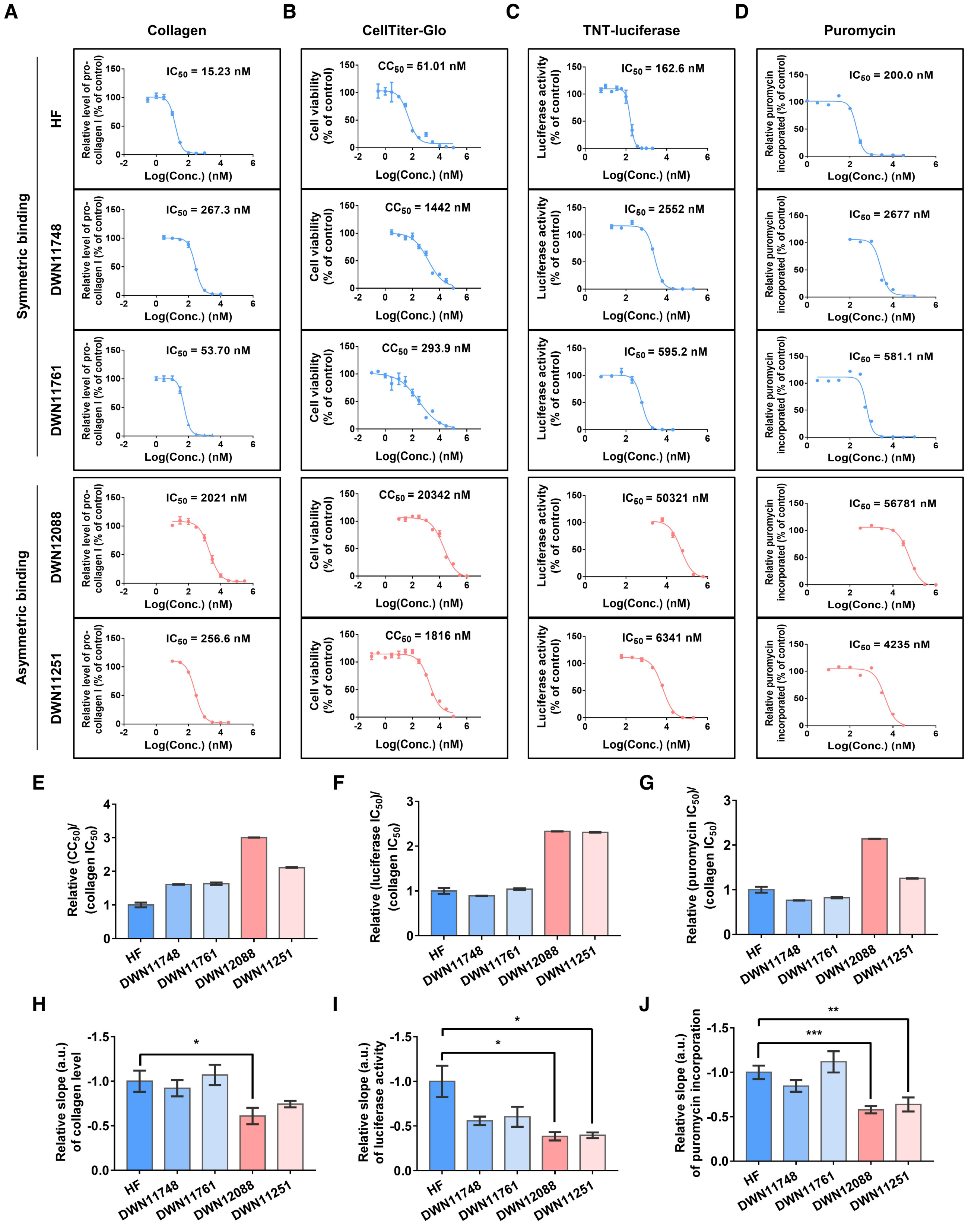

Figure 5.

© 2023 The Authors

Figure 5. Comparative analysis of HF and DWN compounds for their efficacy and toxicity.

A The IC_{50} values of the indicated compounds were determined for collagen level as described in Methods section (HF and DWN12088, $n = 8$ from four independent experiments (duplicate for each experiment); DWN11748, $n = 12$ from six independent experiments (duplicate for each experiment); DWN11761 and DWN11251, $n = 6$ from three independent experiments (duplicate for each experiment); mean ± SEM).

B The IC_{50} values of the indicated compounds were determined for cytotoxicity (CC_{50}) as described in Methods section ($n = 4$ from two independent experiments (duplicate for each experiment); mean ± SEM).

C The IC_{50} values of the indicated compounds were determined for TNT-luciferase activity as described in Methods section ($n = 6$ from two independent experiments (triplicate for each experiment); only for DWN11761, $n = 9$ from three independent experiments (triplicate for each experiment); mean ± SEM).

D The IC_{50} values of the indicated compounds were determined for puromycin incorporation as described in Methods section (HF, $n = 12$ from four independent experiments (triplicate for each experiment); DWN11748, DWN12088, and DWN11251, $n = 9$ from three independent experiments (triplicate for each experiment); DWN11761, $n = 8$ from three independent experiments (triplicate for two experiments and duplicate for one experiment); mean ± SEM).

E–G Relative ratio of CC_{50} (E), TNT-luciferase activity IC_{50} (F) and puromycin incorporation IC_{50} (G) to the IC_{50} of collagen levels shown in (A) was calculated (mean ± SEM). IC_{50} values were determined by using Graph Pad Prim 7.0.

H–J Relative slopes of the dose–response curves shown in (A, C and D) were calculated using GraphPad Prism 7.0 (Welch's *t* test; $^{*}P < 0.05$, $^{**}P < 0.01$, $^{***}P < 0.001$; mean ± SEM).

Source data are available online for this figure.

to the compounds. More specifically, we suspected that when the first compound occupies one protomer for catalytic inhibition, the compound-bound protomer may induce the other promoter to bind less effectively to the second compound, which may result in a higher TI.

To investigate this suspicion, we monitored the effects of the five compounds in terms of collagen level, cytotoxicity, and global translation by determining dose–response curves (Fig 5A–D). The level of global translation was determined by TNT-coupled luciferase activity and puromycin incorporation assays. We then compared the ratios of the IC_{50} values for toxic response assays (cytotoxicity, TNT-coupled luciferase, and puromycin incorporation assays) to those for collagen level assay to estimate margins between efficacy and toxicity (Fig 5E–G). We also analyzed slopes of the dose–response curves to compare dose-dependent responsiveness of the compounds (Fig 5H–J) and analyzed relationship between the binding modes, margins, and slopes. ABC showed relatively wide margins and gentle slopes while SBC showed narrow margins and steep slopes. Considering that the slope of the dose–response curve, Hill slope, reflects the degree of interaction between the ligand-binding sites, these results suggest that binding of ABC to the first protomer would negatively affect their binding to the second protomer of PARS1 and decrease sensitivity of PARS1 inhibition at higher dose, thereby increase safety margin.

Significance of the asymmetric PARS1 binding of DWN12088 on efficacy and toxicity

To test whether asymmetric binding of DWN12088 to PARS1 was responsible for the increased TI, we introduced mutations to PARS1 residues, which play a role in the interaction with proline (E1123), ATP (R1152), and the compounds (F1097, E1123, and R1152) (Fig 6A). PARS1 F1097A/E1123A/R1152L (FA/EA/RL) showed a reduced affinity for the compounds (Fig 6B) and lost its catalytic capability to ligate proline to $tRNA^{Pro}$ without disturbing the catalytic activity of PARS1 WT (Fig 6C). Although kinetic studies of

Figure 6. Effect of PARS1 dimeric interaction on the efficacy and toxicity of HF and DWN12088.

A The role of F1097, E1123 and R1152 in the binding to the compounds, ATP and proline in complex structures of PARS1-HF-ANP (PDB: 4HVC), PARS1-DWN12088-ATP and PARS1-proline (PDB: 4K87). The color codes of the structures are the same as those shown in Fig 3A and B.

B The binding of HF and DWN12088 to *in vitro* purified PARS1 WT and F1097A/E1123A/R1152L (FA/EA/RL) mutant was determined via microscale thermophoresis (MST). K_d values are listed in the top left part of the graph (technical replicates $n = 3$; mean ± SD).

C Catalytic activities of PARS1 WT and FA/EA/RL mutant were determined by prolylation assay in the presence of [^{3}H]proline. To monitor the effect of PARS1 RA/EA/RL mutant on the activity of PARS1 WT, the same concentrations of the proteins were pre-incubated for 10 min and then used for the assay. The formation of Pro-$tRNA^{Pro}$ was measured using liquid scintillation counter (technical replicates $n = 3$; mean ± SEM).

D The kinetic values of PARS1 with homodimer (WT/WT) and heterodimer (WT/MT) settings were determined by prolylation assay in the presence of [^{3}H]proline. MT, PARS1 FA/EA/RL (biological replicates $n \geq 3$; mean ± SEM).

E 293 T cells were transfected with the indicated plasmid DNAs for 24 h. The cell lysates were prepared and pulled down with Strep bead for 2 h. Co-precipitated proteins were determined by immunoblot assay. WT, PARS1 wild type; MT, PARS1 FA/EA/RL.

F Schematic representation of the strategy for generating a monomer-like EPRS1. Endogenous EPRS1 dimer would function as a monomer-like EPRS1 by forming a dimer with exogenous PARS1 mutant that cannot bind to the compounds and does not show the catalytic activity.

G, H WI-26 VA4 cells stably expressing each of EV (empty vector) or PARS1 FA/EA/RL mutant were incubated with the indicated compounds for 72 h. Dose–response curves of the compounds for collagen levels and cytotoxicity were determined and relative ratio of CC_{50} to the IC_{50} of collagen for HF (G) and DWN12088 (H) were calculated. The IC_{50} values were calculated using GraphPad Prism 7.0 (collagen ELISA, $n = 12$ from four independent experiments (triplicate for each experiment); cytotoxicity assay, $n = 8$ from four independent experiments (duplicate for each experiment); Welch's *t* test; $^{***}P < 0.001$; mean ± SEM).

I, J Relative slopes of dose–response curves of HF (I) and DWN12088 (J) for collagen levels were determined in cells stably expressing each of EV or PARS1 FA/EA/RL mutant. The slopes were determined by using GraphPad Prism 7.0 ($n = 3$ in triplicates for collagen ELISA, $n = 4$ in duplicates for cytotoxicity assay; mean ± SEM; Welch's *t* test; $^{***}P < 0.001$; mean ± SEM).

K WI-26 VA4 cells stably expressing each of EV or PARS1 FA/EA/RL mutant were incubated with the indicated compounds in the presence of TGF-β for 72 h. The levels of the indicated proteins were determined by immunoblotting. The percentage of proline in polypeptide sequences is shown on the right side of immunoblot images.

Source data are available online for this figure.

© 2023 The Authors

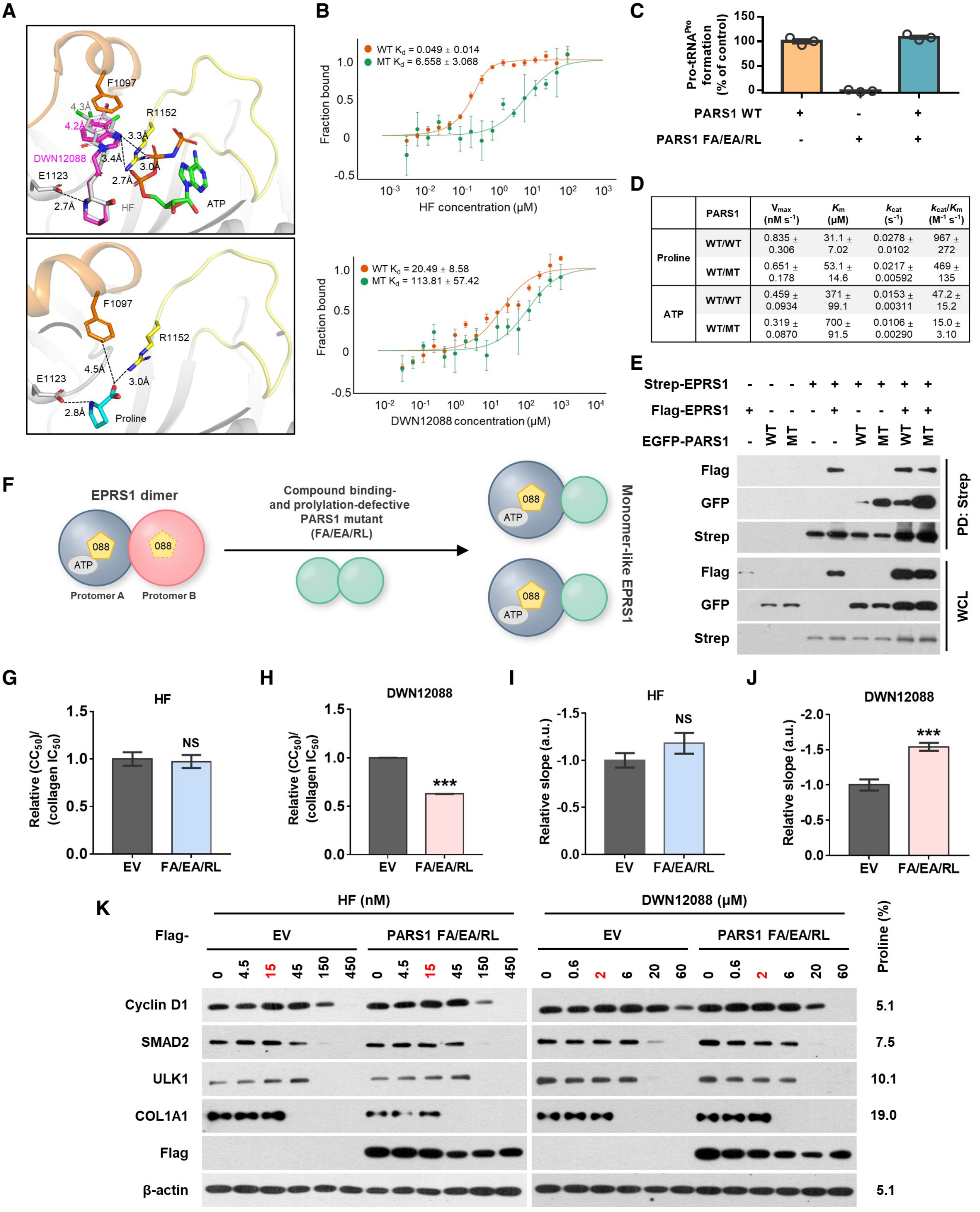

	PARS1	V_{max} (nM s^{-1})	K_m (μM)	k_{cat} (s^{-1})	k_{cat}/K_m (M^{-1} s^{-1})
Proline	WT/WT	0.835 ± 0.306	31.1 ± 7.02	0.0278 ± 0.0102	967 ± 272
	WT/MT	0.651 ± 0.178	53.1 ± 14.6	0.0217 ± 0.00592	469 ± 135
ATP	WT/WT	0.459 ± 0.0934	371 ± 99.1	0.0153 ± 0.00311	47.2 ± 15.2
	WT/MT	0.319 ± 0.0870	700 ± 91.5	0.0106 ± 0.00290	15.0 ± 3.10

Figure 6.

 © 2023 The Authors

PARS1 homodimer (WT/WT) and heterodimer (WT/MT) revealed that PARS1 FA/EA/RL reduced the prolylation activity of wild-type PARS1 (Fig 6D), the effect appeared to be negligible in conditions where the substrates were abundant (Fig 6C). Since PARS1 FA/EA/RL could form a dimer with WT EPRS1 (Fig 6E), we expressed PARS1 FA/EA/RL in WI-26 VA4 cells to prepare EPRS1 heterodimer consisting of EPRS1 WT and PARS1 FA/EA/RL, which is expected to behave as a monomer-like EPRS1 (Fig 6F).

Then, we monitored the dose–response curves of HF and DWN12088 for collagen levels and cytotoxicity assay. The IC_{50} values of DWN12088 in the collagen assay were decreased in cells expressing PARS1 FA/EA/RL while those of HF were similar to those of control cells. The IC_{50} values for cytotoxicity were more significantly decreased in the mutant PARS1, leading to a reduction in the TI of DWN12088 (Fig 6G and H). The slopes of the DWN12088 curves were steeper for mutant-expressing cells compared with the control cells, whereas those of the curves for HF were similar for both cell types (Fig 6I and J). In addition, when comparing the effect of HF and DWN12088 on the levels of general proteins, the levels of proline-rare proteins such as cyclin D1 were decreased more sensitively in mutant-expressing cells as the concentrations of DWN12088 were increased, whereas the sensitivity to HF was similar between control and mutant-expressing cells (Fig 6K). These results validate that the asymmetric binding of DWN12088 to the two protomers of PARS1 via negative cooperativity would contribute to an improved safety compared with HF.

In summary, we demonstrated the possible molecular mechanism underlying the enhanced TI of DWN12088 compared with HF. Since PARS1 is a dimeric protein, the compounds bind to each protomer. As reported previously (Zhou *et al*, 2013), HF binds tightly to both protomers in an almost symmetric mode. On the other hand, when the first DWN12088 molecule binds to PARS1 protomer A, the second DWN12088 molecule could not bind tightly to protomer B due to allosteric effects (Figs 3 and 4). This negative cooperativity would make it difficult to inhibit the prolylation activity of PARS1 protomer B, broadening dose–response curve. Since both the efficacy and toxicity of HF and DWN12088 appear to result from catalytic inhibition of PARS1, the effect of the compounds on protein synthesis may be presented on a single dose–response curve (Synopsis). Proline-rich proteins, such as collagen, would be inhibited at lower concentrations (efficacy dose), and toxic responses induced by the inhibition of general proteins would be initiated at higher concentrations (toxic dose). The difference between the concentrations, or TI, is increased in the shallower dose–response curve. Through this curve, negative cooperativity-dependent TI improvement of DWN12088 can be clearly understood.

Discussion

IPF is a chronic and progressive disease characterized by tissue remodeling with excessive deposition of extracellular matrix leading to fibrosis and lung failure. Unfortunately, there are issues of discontinuation with the FDA-approved drugs, pirfenidone and nintedanib, prompting development of new classes of drugs with clear mode of action. Although the incomplete understanding of the exact pathogenic mechanism underlying IPF makes it difficult to suggest effective pathways to be targeted, reducing collagen accumulation itself has been regarded to have prognostic and therapeutic value in fibrotic diseases by relieving tissue stiffness and preventing prolonged existence of cytokines in tissues (Barry-Hamilton *et al*, 2010; Huang *et al*, 2012; Friedman *et al*, 2013; Parker *et al*, 2014). As an approach to reduce collagen levels, HF, a potent inhibitor of PARS1, was previously suggested as a potential candidate (Granot *et al*, 1993; Wu *et al*, 2020), but has not been approved for clinical usage due to its toxicity concern. In an effort to develop a new type of PARS1 catalytic inhibitor with higher safety, we synthesized HF derivatives and compared them for efficacy and toxicity. The final candidate compound, DWN12088, exhibited about three times improved safety compared with HF in cell-based assays although its effective dose was significantly increased (Fig 1B and C).

To compare efficacy of HF and DWN12088, we utilized two different BLM-induced lung fibrosis models (Fig 1D). While both compounds significantly reduced fibrotic properties in the model 1 (drug administration from day 7) (Fig 1E–I), their effects were less apparent in the model 2 (drug administration from day 14) (Fig EV4G–I). Previous reports investigating the kinetics of lung fibrosis induced by intratracheal injection of BLM showed that inflammatory phase is initially observed (until day 7–10), followed by collagen accumulation (from day 7 to 21), subsequently by spontaneous resolution (from day 21) (Liu *et al*, 2013, 2017). Collagen levels appear to remain constant after day 21, suggesting that collagen synthesis, which is the target process of PARS1 catalytic inhibitors, may be spontaneously decreased during this period. Therefore, the reduced efficacy in the model 2 may be attributed to the combination of pathological process of BLM-induced lung fibrosis and mode of action of the drugs. In a clinical context, the PARS1 catalytic inhibitors such as DWN12088 may be more effective in patients with progressive IPF who exhibit increased levels of collagen synthesis markers compared with patients with stable IPF (Organ *et al*, 2019).

The toxic response patterns of HF and DWN12088 were similar in the 2-week repeated dose toxicity study, implying that the major mechanisms underlying the toxicity of the two compounds would be similar (Table 1). The effective doses of HF for decreasing general proteins, such as SMAD2, AIMP2, AMPKα, or cyclin D1, compared with COL1A1 differed by about three times while those of DWN12088 differed by about 10 times (Fig 2A), consistent with the difference in TI values among the tested compounds (TI_{HF} = 3.46, $TI_{DWN12088}$ = 10.29) (Fig 1C). The effects of HF and the DWN compounds on the dose-dependent changes in global translation gave an insight into the potential mechanism underlying their toxicity (Fig 5). These results suggest that the major cause of toxic responses would result from their inhibitory effects on general protein synthesis. Interestingly, the arithmetic average of proline content in cellular proteins is 6.3%, which is approximately a third of the proportion of proline in COL1A1 (19.0%). Therefore, the threefold difference in effective dose between collagen and general proteins observed in HF appears to be reasonable, and the narrow TI of HF might be attributed to the safety issues observed in clinical trials. In contrast, DWN12088 appears to show better tolerability with an extended TI of around 10, which is generally considered a good safety profile (Tamargo *et al*, 2015). While the increase in TI for DWN12088 may look modest, it could result in significant effect on the toxicity profile. In this regard, it is worth noting the case of theophylline and its derivative, doxofylline. Doxofylline is known to

© 2023 The Authors

improve TI only by a factor of 3–6 compared with theophylline, based on therapeutic dosage and lethal dose 50 (LD_{50}). Nonetheless, doxofylline can be administered to patients without the need to monitor plasma concentrations while theophylline requires careful monitoring to keep safe and effective levels (Page, 2010; Matera *et al*, 2017).

Co-crystal structures of PARS1 complexed with various DWN compounds and ATP were classified into two groups based on their modes of binding to the two protomers of PARS1 (Fig 3C). The allosteric effect resulting from the binding of DWN12088 and DWN11251 to protomer A of PARS1 could disturb the binding of the second compound to protomer B. Interestingly, it appears that the compound-induced negative cooperativity between the two protomers is responsible for the improvement in pharmacological safety. In order to reduce drug toxicity, this strategy can be applied not only to other ARSs but also to other essential enzymes with multimeric forms.

While the catalytic sites of bacterial ARSs have been used to develop anti-infectives, those of human ARSs have not been actively explored for the concern of safety (Francklyn & Mullen, 2019). However, the results of this work encourage that the catalytic sites of ARSs can be pharmacologically targetable if drugs are suitably designed for a mild or partial inhibition of the catalytic activities of ARSs to the levels that do not disturb global protein synthesis. Selection of ARS targets based on amino acid composition of the pathologically associated proteins would further warrant safety and specificity of drug action. Of note, with similar logical background as targeting PARS1 to reduce collagen levels, targeting the catalytic activity of threonyl-tRNA synthetase 1 (TARS1) was shown effective specifically to block biosynthesis of mucin (Jeong *et al*, 2018), which contains threonine up to 35% in the sequence. Since mucin is believed to be crucial for cancer cell migration, TARS1 catalytic inhibitors could be applied to control cancer metastasis. With the rationale of target selection based on amino acid composition and the controlled inhibition of their catalytic activities without causing serious damage to global protein synthesis, the catalytic sites of human ARSs can provide a new therapeutic target space for diverse refractory diseases to specifically inhibit pathogenic synthesis and accumulation of the related proteins.

Our study has several limitations. First, in the experiments examining *in vivo* toxicity, we could not determine the exact NOAEL due to the toxic responses of HF even in low-dose groups (Table 1). The use of drug concentration rather than drug exposure also limits the accurate calculation of TI in the *in vivo* studies (Muller & Milton, 2012). Second, we did not show the PARS1 structure that is composed of protomer A bound to both DWN12088 and ATP, and protomer B bound to only ATP or nothing, despite numerous trials of crystallization. Last, although we suggested negative cooperativity as a strategy to improve toxic response of excessive inhibition to essential drug target, it is difficult to design a drug with negative cooperativity from the current knowledge of medicinal biology. Therefore, future work will also be needed to rational design of this kind of drugs, but at least, monitoring slope of dose–response curves at initial screening stages would provide information about cooperativity.

Overall, our study demonstrates the enhanced TI of DWN12088 compared with HF at the cellular and animal levels, together with the mechanistic understanding and validation at the molecular and cellular levels. This study provides evidence of DWN12088 safety for the control of collagen level in fibrosis as well as a novel strategy for targeting essential proteins with enhanced safety.

Materials and Methods

Materials

Reagents were obtained from following sources. Antibodies to ULK1 (D8H5) (8054, 1:1,000), phosphor-Smad2 (Ser465/467) (138D4) (3108, 1:1,000), AMPKα (D5A2) (5831, 1:2,000), Cyclin D1 (2922, 1:1,000), RagC (3360, 1:1,000), Smad2/3 (D7G7, 1:2,000) (8685) from Cell Signaling Technology; to COL1A1 (SP1.D8, 1:300) from Developmental Studies Hybridoma Bank (DSHB); β-actin (A1978, 1:80,000), Flag (F3165, 1:20,000) from Sigma-Aldrich; to Strep (conjugated to HRP) (2-1509-001, 1:1,000) from IBA Lifesciences; to LARS1 (A304-315A, 1:5,000) from Bethyl Laboratories; to AARS1 (H-268) (sc-98547, 1:1,000) from Santa Cruz Biotechnology; to puromycin (MABE343) from Millipore; to mouse IgG (H + L) secondary antibody (HRP) (31430, 1:20,000), rabbit IgG (H + L) secondary antibody (HRP) (31460, 1:20,000) from Thermo Fisher Scientific. Halofuginone hydrobromide (32481-10MG), DMSO (D2650), ATP disodium salt hydrate (A26209-5G), and tRNA from baker's yeast (10109495001) were obtained from Sigma-Aldrich. L-[2,3,4,5-^{3}H]-Proline (NET483005MC) was obtained from Perkin Elmer.

Cell culture

WI-26 VA4 cells (human) were obtained from Korean Cell Line Bank (KCLB) (10095.1) and authenticated by STR profiling. 293 T cells (human) were obtained from American Type Culture Collection (ATCC) (CRL-3216). LX2 cells (human) were obtained from Millipore (SCC064). WI-26 VA4 and 293 T cells were cultured in Dulbecco's Modified Eagle's Medium (DMEM) (Cytiva, SH30243.01) supplemented with 10% fetal bovine serum (FBS) (Cytiva, SH30084.03) and 1% penicillin–streptomycin (Cytiva, SV30010), and grown in 5% CO_2 at 37°C. LX2 cells were cultured in DMEM supplemented with 2% FBS and 1% penicillin–streptomycin and grown in 5% CO_2 at 37°C.

Normal Human Lung Fibroblast (NHLF) cells were obtained from Lonza (CC-2512, batch number 0000608197; human, 67-year-old, male, Caucasian). NHLF cells were cultured in FBM™-2 Fibroblast Growth Medium-2 BulletKit™ (Lonza, CC-3132) and grown in 5% CO_2 at 37°C.

Transfection

293 T cells were seeded (5×10^5, 6 well scale) and incubated for 24 h in 5% CO_2 at 37°C. pEXPR-105-strep-EPRS1, pCMV6-myc-DDK-EPRS1, pEGFP-N3-PARS1 plasmid DNAs were transfected to the cells, which were starved of serum using Turbofect (Thermo Fisher Scientific, R5031) following the manufacturer's instructions. After 4 h, the transfected cells were recovered with DMEM supplemented with 10% FBS and 1% penicillin–streptomycin and grown for additional 20 h.

WI-26 VA4 and WI-26 VA4$^{Flag\text{-}EPRS1}$ stable cells were seeded (1.5×10^5, 6 well scale) and incubated for 24 h in 5% CO_2 at 37°C.

 © 2023 The Authors

200 pmol of small interference RNAs targeting UTR of EPRS1 (Sense, CUA AGU UAA CAG UGG AUA AUU; Antisense, UUA UCC ACU GUU AAC UUA GUU), LARS1 (Sense, CCA GGG UCA UUG UCG UGG AUU UGC A; Antisense, UGC AAA UCC ACG ACA AUG ACC CUG G) was transfected to the cells using Lipofectamine 3000 (Thermo Fisher Scientific, L3000001) following the manufacturer's instructions. After 4 h, the transfected cells were recovered with DMEM supplemented with 10% FBS and 1% penicillin–streptomycin and grown for an additional 68 h.

Lentivirus transduction and stable cell line establishment

293 T cells were transfected with plasmid DNAs (pLVX-TetOne-puro-EV, pLVX-TetOne-puro-flag-EPRS1, pLVX-TetOne-puro-flag-PARS1 F1097A/E1123A, and pLVX-TetOne-puro-flag-PARS1 F1097A/E1123A/R1125L) using Lenti-X™ Packaging Single Shots (VSV-G) (Takara, 631275) following the manufacturer's instructions and incubated for 48 h to produce lentivirus. After the lentivirus was collected from the supernatant of 293 T cells, WI-26 VA4 cells were infected with the lentivirus. The infected cells were selected using 1 μg/ml puromycin (Takara, 631306) for a week to establish stable cell lines. The protein expression was induced by incubating the cells with 0.5 μg/ml doxycycline (Sigma Aldrich, D9891) for 48 h.

Collagen ELISA

WI-26 VA4 cells were seeded at 3.5×10^4 cells/well on 24 well plates. After 24 h, the cells were starved of serum for 6 h. Then, the cells were incubated with the indicated concentrations of compounds in the presence of TGF-β (2 ng/ml) (R&D systems, 240-B-002) for 72 h. The supernatant of the cells was collected and centrifuged at 500 *g* for 5 min to remove cell debris. Then, the level of pro-collagen I in the supernatant was determined using Human Pro-collagen I alpha 1 DuoSet ELISA (R&D systems, DY6220-05) and DuoSet ELISA Ancillary Reagent Kit 2 (R&D systems, DY008) following the manufacturer's instructions.

CellTiter-Glo assay

To screen HF derivatives, NHLF cells were seeded at 7×10^3 cells/well in 96-well white plates. After 24 h, the cells were incubated with various concentrations of compounds for 48 h. The level of ATP as a measure of cell viability was determined using a CellTiter-Glo® luminescent cell viability assay kit (Promega, G7571) following the manufacturer's instructions.

WI-26 VA4 cells were seeded at 1.2×10^4 cells/well in 96-well white plates. After 24 h, the cells were incubated with the indicated concentrations of compounds for 72 h. The viability was determined as described above.

In vitro prolylation assay

To screen HF derivatives, reaction buffer (20 mM Tris–HCl (pH 7.5), 6 mM MgAc, 0.5 mM DTT, 5 mM ATP, 5 mg/ml yeast total tRNA, and 1 μM [^{3}H]proline (81.4 Ci/mmol)) was pre-warmed at 37°C. The enzyme reaction was initiated by the addition of 100 nM purified His-PARS1 (1001–1,512 aa). After 5 min, the reaction mixture was quenched on Whatman filter paper presoaked with 5% trichloroacetic acid (TCA). The filter papers were washed three times in 5% TCA for 10 min at 4°C and once in 100% ethanol for 10 min. The filter papers were dried, and radioactivity was quantified using liquid scintillation counter (LSC).

To measure the prolylation activity of PARS1 WT and FA/EA/RL, the reaction buffer (20 mM Tris–HCl (pH 7.5), 6 mM MgAc, 0.5 mM DTT, 5 mM ATP, 5 mg/ml yeast total tRNA, 99 μM proline, 1 μM [^{3}H]proline (81.4 Ci/mmol)) was pre-warmed at 37°C, and the enzyme reaction was initiated by the addition of 100 nM purified His-PARS1 (1001–1,512 aa). After 1 h, the reaction mixture was quenched on Whatman filter paper presoaked with 5% TCA. The filter papers were washed three times in 5% TCA for 10 min at 4°C and once in 100% ethanol for 10 min. The filter papers were dried, and radioactivity was quantified using LSC.

To determine the kinetic values of PARS1 homodimer (WT and WT) and heterodimer (WT and FA/EA/RL mutant) proteins, we pre-incubated the proteins for 10 min at room temperature. The reaction buffer A (20 mM Tris–HCl (pH 7.5), 6 mM MgAc, 0.5 mM DTT, 5 mM ATP, 5 mg/ml yeast total tRNA, 1 μM [^{3}H]proline (81.9 Ci/mmol) and various concentrations of proline) was prepared for kinetic of proline, and the reaction buffer B (20 mM Tris–HCl (pH 7.5), 6 mM MgAc, 0.5 mM DTT, 5 mg/ml yeast total tRNA, 49 μM proline, 1 μM [^{3}H]proline (81.9 Ci/mmol) and various concentrations of ATP) was prepared for that of ATP. The reaction buffers were pre-warmed, and the enzyme reaction was initiated by the addition of homodimer (100 nM PARS1 WT) or heterodimer PARS1 (100 nM PARS1 WT and 100 nM PARS1 FA/EA/RL mutant) proteins. The reaction mixture was quenched on Whatman filter paper presoaked with 5% TCA at 1, 2, 3 and 5 min. The filter papers were washed three times in 5% TCA for 10 min at 4°C and once in 100% ethanol for 10 min. The filter papers were dried, and radioactivity was quantified using liquid scintillation counter. By using standard curve of [^{3}H]proline, the absolute amount of reaction product was calculated. The velocity of the reaction was obtained based on a graph of the reaction product versus time. By using the double reciprocal plot of substrate concentration and velocity (Lineweaver-Burk plot), V_{max}, K_M, k_{cat}, and k_{cat}/K_M values were determined.

Determination of collagen level using 3D culture

To screen HF derivatives, LX2 cells were resuspended with growth medium containing 20% Matrigel (BD Bioscience, 354234) and then seeded at 1×10^4 cells/well on a Lipidure-coated U bottom 96-well plate (NOF, LCU96). After 48 h, the cells were treated with TGF-β1 (Sigma-Aldrich, T7039) for 24 h to mimic fibrotic conditions and then additionally incubated in the presence of various concentrations of test compounds for 48 h. To determine the level of collagen, hydroxyproline levels were measured following the manufacturer's instructions (BioVision, K555-100). Briefly, after the cells were incubated with HCl for 3 h at 120°C, the soluble supernatant was prepared using activated charcoal (Sigma-Aldrich, 161551). The samples were dried for 3 h at 60°C and then, incubated with chloramine T mixture for 10 min and Ehrlich's reagent for 90 min at 60°C. The level of hydroxyproline was determined by measuring the absorbance at 540 nm.

© 2023 The Authors

Transverse aortic constriction (TAC) model

To screen HF derivatives, all experiments were conducted following the international guidelines on the ethical use of experimental animals and approved by the Institutional Animal Care and Use Committee of Gyeonggido Business & Science Accelerator Biocenter (IACUC 2016-11-0007). C57B/L6 mice (9-week-old, male) were randomized (n = 10 for each group) and anesthetized with isoflurane. After a piece of a 6.0 silk suture was placed, a 3 mm-long 26-gauge needle was placed between the right innominate artery and left carotid artery. Two loose knots were tied, and the needle was removed. The test compounds (10 mg/kg) were orally administered once daily for 2 weeks, and the thickness of the left ventricle, number of lytic and necrotic cardiac muscle fibers, diameter of cardiac muscle fibers, percentage of perivascular collagen fibers, percentage of interstitial collagen fibers, number of infiltrated inflammatory cells, and hydroxyproline levels were analyzed. The experiment was conducted as a blind test.

Two-week repeated-dose toxicity study

To screen HF derivatives, all experiments were conducted following the international guidelines on the ethical use of experimental animals and approved by the Institutional Animal Care and Use Committee of Daewoong Pharmaceutical (IACUC-15-177). ICR (CrljOri: CD1) mice (7-week-old, male) were randomized (n = 5 for each group) and then orally administered with various amounts of the test compounds once daily for 2 weeks. Data regarding mortality, general clinical signs, body weight, hematology, clinical chemistry, and organ weight were analyzed.

To compare the *in vivo* toxicity of HF and DWN12088, all experiments were conducted following the international guidelines on the ethical use of experimental animals and approved by the Institutional Animal Care and Use Committee of KNOTUS (KNOTUS IACUC 21-KE-250 and KNOTUS IACUC 21-KE-359). ICR (CrljOri: CD1) mice (7-week-old, male, obtained from Koatech) were randomized (n = 6 for each group) and then orally administered with the indicated amounts of HF or DWN12088 once daily for 2 weeks. In the high-dose group, the dose of DWN12088 was reduced from 120 mg/kg to 90 mg/kg from day 6. All of the mice were observed at least once a day for any clinical signs of toxicity. Body weight was measured at day 1, 2, 3, 4, 5, 8, 14, and 15.

After administering the drugs for 2 weeks, mice were fasted overnight and anesthetized with isoflurane. Blood samples were collected in EDTA-coated tubes, and the following parameters were determined using a hematology analyzer (ADVIA 2120, SIEMENS, USA); red blood cell, platelet count, hematocrit, white blood cell, red cell distribution width, hemoglobin concentration distribution width, hemoglobin concentration, neutrophil, mean corpuscular volume, lymphocyte, mean cell hemoglobin, monocyte, mean cell hemoglobin concentration, eosinophil, mean platelet volume, basophil, reticulocytes, and large unstained cells.

For clinical chemistry, the remaining blood samples were incubated in a vacutainer tube with clot activator and then centrifuged to prepare serum. The following parameters were measured in the serum using a chemistry analyzer (7180, Hitachi, Japan) and electrolyte analyzer (AVL 9180, Roche, Switzerland); aspartate aminotransferase, albumin, alanine aminotransferase, albumin/globulin ratio, alkaline phosphatase, blood urea nitrogen, creatine phosphokinase, creatinine, total bilirubin, inorganic phosphorus, glucose, calcium, total cholesterol, sodium, triglyceride, potassium, total protein, and chloride.

After blood collection, blood was drained by incising the abdominal aorta and posterior vena cava, and all organs of the skin, brain, thoracic cavity, and abdominal cavity were observed. The relative weights of the lung, spleen, liver, heart, kidney, thymus, and testes were calculated by dividing the weight of each organ by the body weight. The experiments were conducted as non-blind test.

Bleomycin-induced lung fibrosis model

In a preventive model of bleomycin-induced lung fibrosis, all experiments were conducted following the international guidelines on the ethical use of experimental animals and approved by the Institutional Animal Care and Use Committee of Aragen Bioscience (17-0331-M-1). C57BL/6 mice (6–7-week-old, male, obtained from Simonsen Laboratories) were randomized into groups (n = 10 for each group) such that mean body weights were similar for the different groups. Oral administration of the compounds (vehicle, DWN12088, and pirfenidone) was started a day before oropharyngeal administration of 70 μl bleomycin (2 units/kg). DWN12088 (3, 10, 30 mg/kg) was treated once a day while 100 mg/kg pirfenidone was treated twice a day (200 mg/kg/day) for 3 weeks, and then the outcome was analyzed.

To examine therapeutic efficacy of DWN12088, all experiments were conducted following the international guidelines on the ethical use of experimental animals and approved by the Institutional Animal Care and Use Committee of Daewoong Pharmaceutical (IACUC-21-014). C57BL/6 mice (7–8-week-old, male, obtained from Orient Bio) were anesthetized with Zoletil and injected intratracheally with 100 μl bleomycin (2 units/kg) (Merck, B8416) to induce lung fibrosis. A group of disease-free mice ("untreated") were injected with PBS instead of bleomycin. After a week, mice treated with bleomycin were randomized to (i) vehicle (saline), (ii) HF 0.05 mg/kg, (iii) HF 0.1 mg/kg, and (iv) DWN12088 10 mg/kg groups (n = 5 for each group) according to their body weight. The drugs were orally administered once daily for 2 weeks, and then the outcome was analyzed. For the experiments testing the effect of the compounds 2 weeks after bleomycin challenge, the experiment was approved by IACUC of Daewoong Pharmaceutical (IACUC-22-009). The experimental procedures were performed following the methods described previously, with the exception that the compounds were administered 2 weeks after bleomycin challenge. The experiments were conducted as non-blind tests.

Measurement of peripheral oxygen saturation (SpO_2) levels

Mice were anesthetized with Zoletil for 10 min, and a portable mouse pulse oximeter (Veterinary Pulse Oximeter, Berry Electronic) was placed on each mouse's body. The measurements were continued to allow the recording of 3 min of a stable signal.

Sircol collagen assay

The collagen contents of lung tissues were measured using Sircol™ soluble collagen assay (Biocolor Ltd., S1000) following the manufacturer's instructions. Briefly, the right lung lobes were homogenized, and collagen was solubilized in 0.5 M acetic acid. Tissue extracts

 © 2023 The Authors

were incubated with Sirius red dye, and the absorbance was determined at 540 nm using a spectrophotometer. The amount of collagen was expressed relative to the wet weight of the tissues.

Masson's trichrome staining

The lung tissues were fixed in paraformaldehyde and embedded in paraffin. The paraffin-embedded blocks were sectioned at 4 μm and then mounted onto glass slides. After deparaffinization and rehydration, the slides were fixed with Bouin's solution (Sigma Aldrich, HT10132-1L) for 1 h. Then, the slides were incubated with hematoxylin (Sigma Aldrich, MHS16-500ML), Biebrich Scarlet (Sigma Aldrich, B-6008) and acid fuchsin (Sigma Aldrich, A-3908), phosphomolybdic (DAEJUNG, 6531-4105) and phosphotungstic acid (DAEJUNG, 6539-4105), aniline blue (DAEJUNG, 1087-4125), and then 1% (v/v) acetic acid (DUKSAN, UN2789). After dehydration, the slides were mounted onto cover slips and scanned using slide scanner (3D HISTECH, Pannoramic MIDI). Fibrotic area of the images was quantified by using Image J plugin, color deconvolution 2.

Pharmacokinetic study

All experiments were performed according to the guidelines on the Institutional Animal Care and Use Committee of QuBEST BIO (QBIACUC-A21112). Seven-week-old C57BL/6 male mice were purchased from Samtako (Osan, South Korea) and fed with regular chow and water ad libitum for 1 week before the experiments. Nine C57BL/6 mice in each group received three single ascending doses (3, 10, and 30 mg/kg) of DWN12088 formulated in normal saline intravenously and orally in fasted conditions. Post-dosing serial blood samples were obtained using eye bleeding into heparinized capillary tubes with composite sampling (three mice per time point) at predetermined time points. The plasma was separated from the whole blood by centrifugation at 15,000 *g* for 2 min at 4°C, and then the proteins were precipitated using acetonitrile containing a deuterated-labeled DWN12088 as an internal standard (IS).

The amount of DWN12088 in mouse plasma was measured by the AB Sciex ExionLC HPLC system connected to an AB Sciex 6500+ Qtrap mass spectrometer equipped with an electrospray ionization (ESI) source (AB Sciex LLC, Framingham, MA, USA). A Phenomenex Gemini-NX C18 column (100 × 2 mm, 5 μm) was used for chromatographic separations, which was maintained at 40°C. The mobile phase consisted of 0.1% formic acid in water (A) and acetonitrile (B), which was run at flow rate of 0.4 ml/min. The gradient elution program was set as follows: 0–1 min, 10% B; 1.0–1.5 min, 10–95% B; 1.5–3.6 min, 95% B; 4.0–6.0 min, 10% B. Multiple reaction monitoring (MRM) was selected for the quantification of DWN12088 and IS in positive ion mode. The mass parameters such as transition ion pairs (*m/z*), declustering potential (DP), collision energy (CE), and collision cell exit potential (CXP) were 328.0 → 142.1, 16, 21, and 10 V for DWN12088 and 332.0 → 146.1, 51, 25, and 14 V for IS.

General chemistry

All the commercial chemicals were of reagent grade and were used without further purification. Solvents were dried with standard procedures. All the reactions were carried out under an atmosphere of dried argon in flame-dried glassware. ^1H NMR and ^{13}C NMR spectra were recorded at 500 MHz using a Bruker AVANCE III 500 spectrometer in $CDCl_3$, or dimethyl sulfoxide (DMSO)-d_6 solution, with tetramethylsilane (TMS) serving as the internal standard. Multiplicities of NMR signals were reported using different abbreviations such as singlet (s), doublet (d), triplet (t), quartet (q), and multiplet (m). The chemical shifts are provided in parts per million (ppm) downfield with coupling constants in hertz (Hz). The mass spectra were recorded using high-resolution mass spectrometry (HRMS) (electron ionization MS) obtained on a JMS-700 mass spectrometer (JEOL, Japan) or 6890 mass pectrometer (Agilent, USA). The products from all the reactions were purified by flash column chromatography using silica gel 60 (230–400 mesh Kieselgel 60). Additionally, thin-layer chromatography on 0.25-mm silica plates (E. Merck; silica gel 60F254) was used to monitor reactions. Intermediate product purity was checked by reversed phase high-pressure liquid chromatography (RP-HPLC), performed on a Waters Corp. HPLC system equipped with an ultraviolet (UV) detector set at 260 nm. The mobile phases used were: (A) H_2O containing 0.10% formic acid; and (B) CH_3OH containing 0.10% formic acid. HPLC employed a Kromasil 100-5-C8 column (5-μm particle size) that was 4.6 mm in diameter × 250 mm in size with a flow rate of 1.0 ml/min. Compound purity was assessed using the following method: 0.10% FA in H_2O:0.10% FA in CH_3OH from 60:40 to 10:90. The purity of all chemically evaluated compounds was > 97.0%. Final product purity was checked by reversed phase high-pressure liquid chromatography (RP-HPLC), performed on a Waters Corp. HPLC system equipped with an ultraviolet (UV) detector set at 260 nm. The mobile phases used were: (A) H_2O containing 0.01 M Ammonium acetate; and (B) CH_3OH. HPLC employed a Kromasil 100-5-C8 column (5-μm particle size) that was 4.6 mm in diameter × 250 mm in size with a flow rate of 1.0 ml/min. Compound purity was assessed using the following method: 0.01 M Ammonium acetate in H_2O: CH_3OH from 70:30 to 10:90. The purity of all chemically evaluated compounds was > 99.0%.

Purification and crystallization of human PARS1 with DWN compounds and ATP

The overall expression and purification steps of human PARS1 in *Escherichia coli* have been described in the previous report (*35*). At the end of the purification, the buffer of purified PARS1 was changed to storage buffer (2 mM Tris–HCl (pH 8.0), 50 mM NaCl, 5 mM β-mercaptoethanol) using an Amicon tube (Merck Millipore). Finally purified PARS1 was concentrated to 50 mg/ml and stored at −80 °C. The co-crystallized crystals of PARS1 with various DWN compounds and ATP were obtained under the same condition as previous reported (*34*). Briefly, 40 mg/ml of purified PARS1 was incubated with 5 mM DWN compounds and 5 mM ATP for 30 min. One microliter of the PARS1 mixture was mixed with 1 μl of the reservoir solution (50 mM HEPES-Ca$(OH)_2$ (pH 7.5), 20% (w/v) polyethylene glycol 3350 and 0.6 M $CaCl_2$) and then incubated at 20 °C using the hanging-drop vapor-diffusion method. Three days later, the crystals of PARS1 appeared.

Data collection and structure determination

The crystals of PARS1 were frozen in liquid nitrogen using the solution containing 25% of glycerol as a cryoprotectant. The X-ray

diffraction data were collected using a PILATUS3 6 M detector (Dectris, Baden-Daettwil, Switzerland) of beamline 11C at the Pohang Light Source (Pohang, South Korea). Diffraction images were indexed and scaled using HKL-2000 (Otwinowski & Minor, 1997). The structure of PARS1-DWN compound complex was determined by molecular replacement using a PARS1 structure from PDB entry 4hvc (Zhou *et al*, 2013) as a search model with the Phaser (Adams *et al*, 2010) module in PHENIX. Next, pdb files and cif files of DWN compound were generated by using eLBOW (Moriarty *et al*, 2009) module in PHENIX. Further model building was performed using Coot (Emsley *et al*, 2010), and refinement (Afonine *et al*, 2012) was performed using PHENIX. The final model of the PARS1-DWN compound complex structures was validated using MolProbity (Chen *et al*, 2010). Data collection and refinement statistics are provided in Table 2.

TNT-luciferase activity assay

Luciferase protein was synthetized *in vitro* in the presence of the indicated concentrations of compounds using the TNT® Quick Coupled Transcription/Translation System (Promega, L1170) following the manufacturer's instructions. Briefly, T7 luciferase plasmid DNA was mixed with TnT Quick Master Mix, methionine, and the various concentrations of the compounds and incubated at 30°C for 90 min. The final concentration of DMSO was 2% (v/v). The reaction was terminated by incubating the tubes in ice. The reaction mixture was diluted 10-fold using 25 mM Tris–HCl (pH 7.5), and 3 μl of the diluted mixture was incubated with 50 μl of luciferase assay substrate (luciferin) (Promega, E1501) to measure luminescence intensity.

Puromycin incorporation assay

WI-26 VA4 cells were seeded at 1.8×10^5 cells/well on 6-well plates. After 18–24 h, the cells were treated with the indicated concentrations of the compounds for 72 h. The cells were incubated with 5 μg/ml puromycin for the last 10 min. After being rinsed with cold PBS twice, the cells were collected and resuspended in 200 μl of the carbonate bicarbonate buffer (Sigma Aldrich, C3041-50CAP) with protease inhibitor. The cell lysates were obtained by sonication and centrifugation at 20,000 *g* for 15 min at 4°C. After protein quantification using a Pierce™ BCA Protein Assay Kit (Thermo Fisher Scientific, 23225), 400 ng of the proteins was coated to ELISA plates for 18 h at 4°C. After being rinsed twice with the washing buffer (1× PBS with 0.05% (v/v) Tween 20), the plates were incubated with the blocking solution (1% (w/v) bovine serum albumin (A7906-50G) in the washing buffer) (1 h), anti-puromycin antibody (1:5,000) (2 h) and anti-mouse IgG (H + L) secondary antibody (HRP) (1:20,000) (1 h) at 25°C. The plates were rinsed with the washing buffer for three times after each antibody incubation step. Then, puromycin incorporated proteins were visualized with 3,3′,5,5′-tetramethylbenzidine (TMB) solution for 30 min at 37°C, and the reaction was stopped using 5N H_2SO_4. The amount of puromycin incorporated in proteins was determined by measuring the absorbance at 450 nm (reference wavelength at 620 nm). Experimental values marked in red with strikethrough text in the Figure Source Data file were excluded from the analysis. These values were determined by considering the values of biological and technical replicates under the same condition, as well as the values at the former and later chemical concentrations.

The paper explained

Problem
There are high unmet needs for the treatment of idiopathic pulmonary fibrosis (IPF) due to the lack of drugs with desirable efficacy and safety margin. Considering the high content of proline in collagen, the active site of prolyl-tRNA synthetase 1 (PARS1) has been considered as a target point for fibrotic diseases. However, possible adverse effects that might result from the catalytic inhibition of PARS1 shed a concern to drug discovery.

Results
We developed a compound, DWN12088, as a novel PARS1 catalytic inhibitor with enhanced safety that passed human clinical phase 1 studies. Mechanistically, DWN12088 binds to PARS1 homodimer with asymmetric mode to differently inhibit the two catalytic sites. This unique mode of action resulted in the improved therapeutic index of DWN12088 in IPF models.

Impact
This work suggests DWN12088 as a new potential drug to control fibrotic diseases such as IPF. In scientific perspectives, the data demonstrated that the catalytic sites of PARS1 (and potentially those of other aminoacyl-tRNA synthetases) can be explored as an effective pharmacological target.

Surface plasmon resonance (SPR)

Binding properties of PARS1 with HF or DWN12088 were analyzed using BIACORE T200 instrument (GE-Healthcare, Little Chalfont, UK). The optical bio-sensor was equipped with Series S CM5 chip (Cytiva, Sweden). The first flow cell of the chip was prepared as a reference while the second flow cell was coupled with His-PARS1. For His-PARS1 immobilization, the chip was primed with HBS-N (10 mM HEPES-NaOH (pH 7.4), 150 mM NaCl) and then activated by a flow (10 μl/min) of the 1:1 mixture of 400 mM 1-ethyl-3-(3-dimethylaminopropyl) carbodiimide (EDC) and 100 mM N-hydroxysuccinimide (NHS) for 7 min at 25°C. His-PARS1 was diluted to 40 μg/ml in 10 mM sodium acetate (pH 5.5) and then run at 10 μl/min until it reached 6,050 ± 100 RU. The chip was blocked with 1 M ethanolamine (pH 8.5) for 7 min. The immobilized chip was equilibrated eight times with running buffer (TBS-T; 20 mM Tris–HCl (pH 7.4), 150 mM NaCl, and 0.05% (v/v) Tween 20) containing 5% (v/v) DMSO. HF and DWN12088 were dissolved in the running buffer containing 5% DMSO at final concentrations of 0.03–100 μM and 0.1–300 μM, respectively. For the experiments in the presence of ATP, the running buffer was additionally supplemented with 1 mM of ATP, and HF and DWN12088 were tested at final concentrations of 0.003–10 μM and 0.1–100 μM, respectively. The prepared samples (HF and DWN12088) were injected at flow rates of 50 μl/min for 120 s, followed by dissociation for 300 s. After each binding reaction, the chip was readily regenerated by an injection of the running buffer at flow rate 50 μl/min for 30 s. Solvent correction was also performed by injecting eight solutions of the running buffer containing 4.5–5.8% DMSO and repeated after the cycle using the final sample. The binding sensorgrams were

© 2023 The Authors

estimated for all samples using the obtained reference-subtracted sensorgrams (sensorgram of flow cell 2 minus that of flow cell 1) by conducting a DMSO calibration and correction. Then, the steady-state affinity model was applied using the BIA evaluation software version 3.0 (Biacore AB, Uppsala, Sweden).

Pull-down assay

293 T cells were transfected with plasmid DNAs (empty vector, pEXPR-105-EPRS1, pCMV6-myc-DDK-EPRS1, pEGFP-N3-PARS1, and pEGFP-N3-PARS1 F1097A/E1123A/R1152L) for 24 h. After being rinsed with cold 1X PBS twice, the cells were lysed with the lysis buffer (20 mM HEPES-NaOH (pH 7.4), 150 mM NaCl, 5 mM $MgCl_2$, 0.5% Triton X-100, protease inhibitor (Calbiochem, 535140), and phosphatase inhibitor (Thermo Fisher Scientific, 78427)) for 30 min at 4°C. The cell lysates were obtained by centrifugation at 20,000 *g* for 15 min at 4°C. Protein levels were quantified using a Pierce™ BCA Protein Assay Kit (Thermo Fisher Scientific, 23225), and 100 μg of proteins was incubated with 10 μl of MagStrep type3 XT beads for 2 h at 4°C. After the supernatant was removed, the beads were incubated with 1 ml of the lysis buffer for 5 min at 4°C three times. The proteins pulled down with the beads were resuspended with the sample buffer (12.5 mM Tris–HCl (pH 6.8), 2% glycerol, 0.4% SDS, 143 mM β-mercaptoethanol, 0.01% BPB, 2 mM DTT) and denatured for 10 min at 100°C.

Microscale thermophoresis (MST)

MST was performed to study the biomolecular interaction of PARS1 with HF or DWN 12088 under conditions of sufficient ATP. PARS1 WT or mutant was labeled fluorescently using a His-tag Labeling Kit (The Monolith His-Tag Labeling Kit RED-tris-NTA 2nd generation, Nanotemper Technologies) at a constant concentration of 315 nM while ATP concentration was kept at 5 mM. HF and DWN12088 were serially diluted to 16 concentrations starting at 100 μM and 1 mM, respectively. The experiments were performed in PBS with 0.05% (w/v) Tween-20. A series of 16 1:1 protein-ligand dilutions were incubated for 30 min. The samples were loaded into capillaries (Monolith NT.115 capillaries, Nanotemper Technologies). The measurements were performed at 40% LED power and 40% MST power with 30 s laser on time. The affinity constant (K_d) was calculated from a fitted curve using the MO Affinity Analysis software (Nanotemper). All measurements were performed in triplicate.

Statistics

Statistical analysis was performed using Prism 7.0 (GraphPad) including One-way ANOVA, Kruskal–Wallis, Mann–Whitney, and Welch's *t* test. Bar graphs were plotted as mean ± SEM. *P*-values < 0.05 were considered statistically significant.

Data availability

The accession numbers for the structures of PARS1-DWN11251, PARS1-DWN11748, PARS1-DWN11761, and PARS1-DWN12088 reported in this paper are PDB: 7Y28 (http://identifiers.org/pdb/7Y28), PDB: 7Y1H (http://identifiers.org/pdb/7Y1H), PDB: 7Y3S (http://identifiers.org/pdb/7Y3S), and PDB: 7Y1W (http://identifiers.org/pdb/7Y1W), respectively.

Expanded View for this article is available online.

Acknowledgments
This work was supported by the following grants: National Research Foundation of Korea (NRF) grant funded by the Korean government (Ministry of Science and ICT (MSIT)) (2021R1A3B1076605) (Sunghoon Kim). NRF grant funded by Korean government (MSIT) (2020R1A2C2005670) (K.Y.H.). NRF grant funded by Korean government (MSIT) (2021R1C1C1013332) (I.Y.). Yonsei University Research Fund (2020-22-0358, 2020-22-0356 and 2021-22-0061) (Sunghoon Kim). Korea Drug Development Fund (KDDF) grant funded by Korean government (MSIT, Ministry of Trade, Industry, and Energy (MOTIE), and Ministry of Health and Welfare (MOHW)) (KDDF-201812-20) (J.S.P.).

Author contributions
Ina Yoon: Conceptualization; funding acquisition; investigation; visualization; methodology; writing – original draft; project administration. **Sulhee Kim:** Investigation; visualization; methodology. **Minjae Cho:** Conceptualization; investigation; methodology. **Kyung Ah You:** Investigation; methodology. **Jonghyeon Son:** Investigation; visualization; methodology. **Caroline Lee:** Investigation; methodology. **Ji Hun Suh:** Investigation; methodology. **Da-Jeong Bae:** Investigation; visualization; methodology. **Jong Min Kim:** Investigation; visualization; methodology. **Sinae Oh:** Investigation; methodology. **Songhwa Park:** Investigation; methodology. **Sanga Kim:** Investigation; methodology. **Seong Hyeok Cho:** Investigation; methodology. **Seonha Park:** Investigation; methodology. **Kyuhyeon Bang:** Investigation; methodology. **Minjeong Seo:** Investigation; methodology. **Jong Hyun Kim:** Investigation; methodology. **Bong Yong Lee:** Investigation. **Joon Seok Park:** Conceptualization; funding acquisition; project administration. **Kwang Yeon Hwang:** Conceptualization; funding acquisition; writing – original draft; project administration; writing – review and editing. **Sunghoon Kim:** Conceptualization; supervision; funding acquisition; project administration; writing – review and editing.

Disclosure and competing interests statement
MC and BL are the inventors of a published patent related to this work filed by Daewoong Pharmaceutical Co., Ltd. JSP is the director of the Drug Discovery Center of Daewoong Pharmaceutical Co., Ltd. MC, CL, DJB, and JMK are currently employed by Daewoong Pharmaceutical Co., Ltd. BL was an employee of Daewoong Pharmaceutical Co., Ltd.

For more information
http://aibi.re.kr/.

References

Adams PD, Afonine PV, Bunkoczi G, Chen VB, Davis IW, Echols N, Headd JJ, Hung LW, Kapral GJ, Grosse-Kunstleve RW *et al* (2010) PHENIX: a comprehensive python-based system for macromolecular structure solution. *Acta Crystallogr D Biol Crystallogr* 66: 213–221

Afonine PV, Grosse-Kunstleve RW, Echols N, Headd JJ, Moriarty NW, Mustyakimov M, Terwilliger TC, Urzhumtsev A, Zwart PH, Adams PD (2012) Towards automated crystallographic structure refinement with phenix.refine. *Acta Crystallogr D Biol Crystallogr* 68: 352–367

© 2023 The Authors

Assis PA, De Figueiredo-Pontes LL, Lima AS, Leao V, Candido LA, Pintao CT, Garcia AB, Saggioro FP, Panepucci RA, Chahud F *et al* (2015) Halofuginone inhibits phosphorylation of SMAD-2 reducing angiogenesis and leukemia burden in an acute promyelocytic leukemia mouse model. *J Exp Clin Cancer Res* 34: 65

Bando M, Yamauchi H, Ogura T, Taniguchi H, Watanabe K, Azuma A, Homma S, Sugiyama Y, Japan Pirfenidone Clinical Study Group (2016) Clinical experience of the long-term use of pirfenidone for idiopathic pulmonary fibrosis. *Intern Med* 55: 443 – 448

Barratt SL, Mulholland S, Al Jbour K, Steer H, Gutsche M, Foley N, Srivastava R, Sharp C, Adamali HI (2018) South-west of England's experience of the safety and tolerability pirfenidone and nintedanib for the treatment of idiopathic pulmonary fibrosis (IPF). *Front Pharmacol* 9: 1480

Barry-Hamilton V, Spangler R, Marshall D, McCauley S, Rodriguez HM, Oyasu M, Mikels A, Vaysberg M, Ghermazien H, Wai C *et al* (2010) Allosteric inhibition of lysyl oxidase-like-2 impedes the development of a pathologic microenvironment. *Nat Med* 16: 1009 – 1017

Budi EH, Schaub JR, Decaris M, Turner S, Derynck R (2021) TGF-beta as a driver of fibrosis: physiological roles and therapeutic opportunities. *J Pathol* 254: 358 – 373

Carrington R, Jordan S, Pitchford SC, Page CP (2018) Use of animal models in IPF research. *Pulm Pharmacol Ther* 51: 73 – 78

Chen VB, Arendall WB 3rd, Headd JJ, Keedy DA, Immormino RM, Kapral GJ, Murray LW, Richardson JS, Richardson DC (2010) MolProbity: all-atom structure validation for macromolecular crystallography. *Acta Crystallogr D Biol Crystallogr* 66: 12 – 21

Coatney GR, Cooper WC, Culwell WB, White WC, Imboden CA Jr (1950) Studies in human malaria. XXV. Trial of febrifugine, an alkaloid obtained from Dichroa febrifuga lour., against the Chesson strain of Plasmodium vivax. *J Natl Malar Soc* 9: 183 – 186

Emsley P, Lohkamp B, Scott WG, Cowtan K (2010) Features and development of Coot. *Acta Crystallogr D Biol Crystallogr* 66: 486 – 501

Francklyn CS, Mullen P (2019) Progress and challenges in aminoacyl-tRNA synthetase-based therapeutics. *J Biol Chem* 294: 5365 – 5385

Friedman SL, Sheppard D, Duffield JS, Violette S (2013) Therapy for fibrotic diseases: nearing the starting line. *Sci Transl Med* 5: 167sr161

Granot I, Bartov I, Plavnik I, Wax E, Hurwitz S, Pines M (1991) Increased skin tearing in broilers and reduced collagen synthesis in skin in vivo and in vitro in response to the coccidiostat halofuginone. *Poult Sci* 70: 1559 – 1563

Granot I, Halevy O, Hurwitz S, Pines M (1993) Halofuginone: an inhibitor of collagen type I synthesis. *Biochim Biophys Acta* 1156: 107 – 112

Huang X, Yang N, Fiore VF, Barker TH, Sun Y, Morris SW, Ding Q, Thannickal VJ, Zhou Y (2012) Matrix stiffness-induced myofibroblast differentiation is mediated by intrinsic mechanotransduction. *Am J Respir Cell Mol Biol* 47: 340 – 348

Hubner RH, Gitter W, El Mokhtari NE, Mathiak M, Both M, Bolte H, Freitag-Wolf S, Bewig B (2008) Standardized quantification of pulmonary fibrosis in histological samples. *Biotechniques* 44: 514 – 507

Jeong SJ, Kim JH, Lim BJ, Yoon I, Song JA, Moon HS, Kim D, Lee DK, Kim S (2018) Inhibition of MUC1 biosynthesis via threonyl-tRNA synthetase suppresses pancreatic cancer cell migration. *Exp Mol Med* 50: e424

Jiang S, Zhang L, Cui D, Yao Z, Gao B, Lin J, Wei D (2016) The important role of halogen bond in substrate selectivity of enzymatic catalysis. *Sci Rep* 6: 34750

de Jonge MJ, Dumez H, Verweij J, Yarkoni S, Snyder D, Lacombe D, Marreaud S, Yamaguchi T, Punt CJ, van Oosterom A *et al* (2006) Phase I and pharmacokinetic study of halofuginone, an oral quinazolinone derivative in patients with advanced solid tumours. *Eur J Cancer* 42: 1768 – 1774

Katsyv I, Wang M, Song WM, Zhou X, Zhao Y, Park S, Zhu J, Zhang B, Irie HY (2016) EPRS is a critical regulator of cell proliferation and estrogen signaling in ER+ breast cancer. *Oncotarget* 7: 69592 – 69605

Keller TL, Zocco D, Sundrud MS, Hendrick M, Edenius M, Yum J, Kim YJ, Lee HK, Cortese JF, Wirth DF *et al* (2012) Halofuginone and other febrifugine derivatives inhibit prolyl-tRNA synthetase. *Nat Chem Biol* 8: 311 – 317

Kim S, You S, Hwang D (2011) Aminoacyl-tRNA synthetases and tumorigenesis: more than housekeeping. *Nat Rev Cancer* 11: 708 – 718

King TE Jr, Bradford WZ, Castro-Bernardini S, Fagan EA, Glaspole I, Glassberg MK, Gorina E, Hopkins PM, Kardatzke D, Lancaster L *et al* (2014) A phase 3 trial of pirfenidone in patients with idiopathic pulmonary fibrosis. *N Engl J Med* 370: 2083 – 2092

Koon HB, Fingleton B, Lee JY, Geyer JT, Cesarman E, Parise RA, Egorin MJ, Dezube BJ, Aboulafia D, Krown SE (2011) Phase II AIDS malignancy consortium trial of topical halofuginone in AIDS-related Kaposi sarcoma. *J Acquir Immune Defic Syndr* 56: 64 – 68

Kwon NH, Fox PL, Kim S (2019) Aminoacyl-tRNA synthetases as therapeutic targets. *Nat Rev Drug Discov* 18: 629 – 650

Ley B, Collard HR, King TE Jr (2011) Clinical course and prediction of survival in idiopathic pulmonary fibrosis. *Am J Respir Crit Care Med* 183: 431 – 440

Liu W, Wan J, Han JZ, Li C, Feng DD, Yue SJ, Huang YH, Chen Y, Cheng QM, Li Y *et al* (2013) Antiflammin-1 attenuates bleomycin-induced pulmonary fibrosis in mice. *Respir Res* 14: 101

Liu T, De Los Santos FG, Phan SH (2017) The bleomycin model of pulmonary fibrosis. *Methods Mol Biol* 1627: 27 – 42

Liu H, Fredimoses M, Niu P, Liu T, Qiao Y, Tian X, Chen X, Kim DJ, Li X, Liu K *et al* (2021) EPRS/GluRS promotes gastric cancer development via WNT/GSK-3beta/beta-catenin signaling pathway. *Gastric Cancer* 24: 1021 – 1036

Lu Y, Wang Y, Zhu W (2010) Nonbonding interactions of organic halogens in biological systems: implications for drug discovery and biomolecular design. *Phys Chem Chem Phys* 12: 4543 – 4551

Matera MG, Page C, Cazzola M (2017) Doxofylline is not just another theophylline! *Int J Chron Obstruct Pulmon Dis* 12: 3487 – 3493

Mei Q, Liu Z, Zuo H, Yang Z, Qu J (2021) Idiopathic pulmonary fibrosis: an update on pathogenesis. *Front Pharmacol* 12: 797292

Moriarty NW, Grosse-Kunstleve RW, Adams PD (2009) electronic Ligand Builder and Optimization Workbench (eLBOW): a tool for ligand coordinate and restraint generation. *Acta Crystallogr D Biol Crystallogr* 65: 1074 – 1080

Muller PY, Milton MN (2012) The determination and interpretation of the therapeutic index in drug development. *Nat Rev Drug Discov* 11: 751 – 761

Organ LA, Duggan AR, Oballa E, Taggart SC, Simpson JK, Kang'ombe AR, Braybrooke R, Molyneaux PL, North B, Karkera Y *et al* (2019) Biomarkers of collagen synthesis predict progression in the PROFILE idiopathic pulmonary fibrosis cohort. *Respir Res* 20: 148

Otwinowski Z, Minor W (1997) Processing of X-ray diffraction data collected in oscillation mode. *Methods Enzymol* 276: 307 – 326

Page CP (2010) Doxofylline: a "novofylline". *Pulm Pharmacol Ther* 23: 231 – 234

Parker MW, Rossi D, Peterson M, Smith K, Sikstrom K, White ES, Connett JE, Henke CA, Larsson O, Bitterman PB (2014) Fibrotic extracellular matrix activates a profibrotic positive feedback loop. *J Clin Invest* 124: 1622 – 1635

Pines M, Nagler A (1998) Halofuginone: a novel antifibrotic therapy. *Gen Pharmacol* 30: 445 – 450

Richeldi L, du Bois RM, Raghu G, Azuma A, Brown KK, Costabel U, Cottin V, Flaherty KR, Hansell DM, Inoue Y *et al* (2014) Efficacy and safety of nintedanib in idiopathic pulmonary fibrosis. *N Engl J Med* 370: 2071 – 2082

Richeldi L, Collard HR, Jones MG (2017) Idiopathic pulmonary fibrosis. *Lancet* 389: 1941 – 1952

 © 2023 The Authors

Roberts AB, Sporn MB, Assoian RK, Smith JM, Roche NS, Wakefield LM, Heine UI, Liotta LA, Falanga V, Kehrl JH *et al* (1986) Transforming growth factor type beta: rapid induction of fibrosis and angiogenesis in vivo and stimulation of collagen formation in vitro. *Proc Natl Acad Sci USA* 83: 4167 – 4171

Roffe S, Hagai Y, Pines M, Halevy O (2010) Halofuginone inhibits Smad3 phosphorylation via the PI3K/Akt and MAPK/ERK pathways in muscle cells: effect on myotube fusion. *Exp Cell Res* 316: 1061 – 1069

Ryley JF, Betts MJ (1973) Chemotherapy of chicken coccidiosis. *Adv Pharmacol Chemother* 11: 221 – 293

Schwanhausser B, Busse D, Li N, Dittmar G, Schuchhardt J, Wolf J, Chen W, Selbach M (2011) Global quantification of mammalian gene expression control. *Nature* 473: 337 – 342

Sgalla G, Iovene B, Calvello M, Ori M, Varone F, Richeldi L (2018) Idiopathic pulmonary fibrosis: pathogenesis and management. *Respir Res* 19: 32

Sime PJ, Xing Z, Graham FL, Csaky KG, Gauldie J (1997) Adenovector-mediated gene transfer of active transforming growth factor-beta1 induces prolonged severe fibrosis in rat lung. *J Clin Invest* 100: 768 – 776

Son J, Lee EH, Park M, Kim JH, Kim J, Kim S, Jeon YH, Hwang KY (2013) Conformational changes in human prolyl-tRNA synthetase upon binding of the substrates proline and ATP and the inhibitor halofuginone. *Acta Crystallogr D Biol Crystallogr* 69: 2136 – 2145

Song DG, Kim D, Jung JW, Nam SH, Kim JE, Kim HJ, Kim JH, Pan CH, Kim S, Lee JW (2018) Glutamyl-prolyl-tRNA synthetase regulates epithelial expression of mesenchymal markers and extracellular matrix proteins: implications for idiopathic pulmonary fibrosis. *Front Pharmacol* 9: 1337

Song DG, Kim D, Jung JW, Nam SH, Kim JE, Kim HJ, Kim JH, Lee SJ, Pan CH, Kim S *et al* (2019) Glutamyl-prolyl-tRNA synthetase induces fibrotic extracellular matrix via both transcriptional and translational mechanisms. *FASEB J* 33: 4341 – 4354

Sundrud MS, Koralov SB, Feuerer M, Calado DP, Kozhaya AE, Rhule-Smith A, Lefebvre RE, Unutmaz D, Mazitschek R, Waldner H *et al* (2009) Halofuginone inhibits TH17 cell differentiation by activating the amino acid starvation response. *Science* 324: 1334 – 1338

Tamargo J, Le Heuzey JY, Mabo P (2015) Narrow therapeutic index drugs: a clinical pharmacological consideration to flecainide. *Eur J Clin Pharmacol* 71: 549 – 567

Toyoshima K, Nakamura M, Adachi Y, Imaizumi A, Hakamada T, Abe Y, Kaneko E, Takahashi S, Shimokado K (2017) Increased plasma proline concentrations are associated with sarcopenia in the elderly. *PLoS One* 12: e0185206

Wolters PJ, Collard HR, Jones KD (2014) Pathogenesis of idiopathic pulmonary fibrosis. *Annu Rev Pathol* 9: 157 – 179

Wu J, Subbaiah KCV, Xie LH, Jiang F, Khor ES, Mickelsen D, Myers JR, Tang WHW, Yao P (2020) Glutamyl-prolyl-tRNA synthetase regulates proline-rich pro-fibrotic protein synthesis during cardiac fibrosis. *Circ Res* 127: 827 – 846

Zhou H, Sun L, Yang XL, Schimmel P (2013) ATP-directed capture of bioactive herbal-based medicine on human tRNA synthetase. *Nature* 494: 121 – 124

Zion O, Genin O, Kawada N, Yoshizato K, Roffe S, Nagler A, Iovanna JL, Halevy O, Pines M (2009) Inhibition of transforming growth factor beta signaling by halofuginone as a modality for pancreas fibrosis prevention. *Pancreas* 38: 427 – 435

License: This is an open access article under the terms of the Creative Commons Attribution License, which permits use, distribution and reproduction in any medium, provided the original work is properly cited.

Expanded View Figures

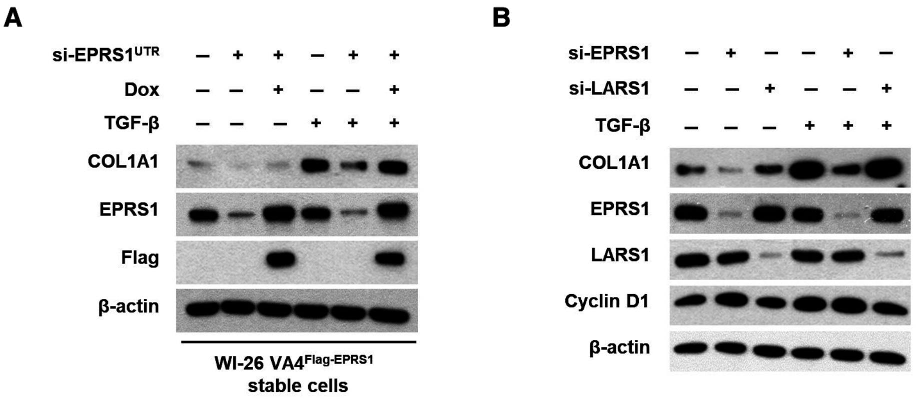

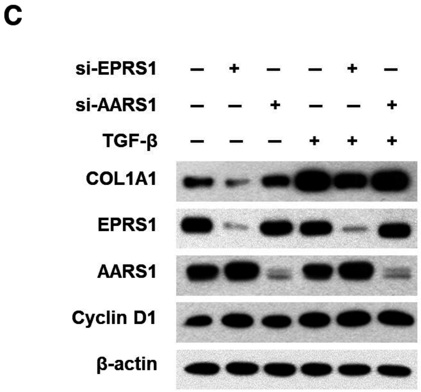

D

Protein	Proline %	Half-life* (h)
COL1A1	19.0	2.9
Cyclin D1	5.1	1.3
EPRS1	5.4	193
LARS1	5.1	262
AARS1	3.6	135
β-actin	5.1	61.9

Figure EV1. Significance of EPRS1 for cellular collagen synthesis.

A WI-26 VA4 cells were made to stably express Flag-EPRS1 in the presence of doxycycline (WI-26 VA4$^{Flag\text{-}EPRS1}$ stable cells). The cells were transfected with negative control or untranslated region (UTR) of EPRS1-targeting siRNAs for 72 h. The cells were also incubated in the presence of doxycycline for 68 h. After starved of serum for 6 h, the cells were treated with TGF-β for 15 h. The level of COL1A1 was determined by immunoblot assay. The level of β-actin was used as loading control.

B, C WI-26 VA4 cells were transfected with the indicated siRNAs for 72 h and incubated with TGF-β for 15 h. The level of COL1A1 was determined by immunoblot assay.

D The proportion of proline and the half-lives of proteins detected in (B) and (C) are listed in the table. The proportion of proline in the polypeptide sequence was calculated based on UniProtKB. The half-lives of proteins were cited from a study that measured half-lives of whole proteome in NIH3T3 cells via mass spectrometry (Schwanhausser *et al*, 2011).

Source data are available online for this figure.

© 2023 The Authors

Figure EV2. Screening workflow and selection criteria of HF derivatives.

A Strategy used in screening for HF derivatives. In total, 523 synthesized compounds were tested for their efficacy in inhibiting the catalytic activity of PARS1 *in vitro*. Among them, 92 compounds which could inhibit PARS1 activity by more than 80% at 1 μM were further examined to obtain their IC_{50} values. Twenty-eight compounds that showed IC_{50} values lower than 100 nM were tested for their efficacy to reduce collagen in cell. Among them, 13 compounds that suppressed collagen levels by more than 50% at 1 μM were tested at multiple doses and 8 compounds showing IC_{50} values lower than 400 nM were tested for cytotoxicity. By comparing the IC_{50} values of collagen levels to the CC_{50} values, two compounds were selected and further tested in *in vivo* efficacy and *in vivo* toxicity models. DWN12088 was finally selected by considering both its efficacy and toxicity doses *in vivo*.

B Heat-map of the inhibitory activities of 523 DWN compounds in *in vitro* prolylation assay at 1 μM.

C Heat-map of the inhibitory activities of 28 DWN compounds to collagen levels in 3D cell culture at 1 μM.

D IC_{50} values for collagen level in 3D cell culture and CC_{50} values of eight compounds were listed in the table. The ratio of CC_{50} to collagen IC_{50} was calculated to estimate TI.

E–H *In vivo* Efficacy of DWN compounds was determined in a transverse aortic constriction model. Pirfenidone was used as a positive control for anti-fibrotic effect. The thickness of left ventricle (E), percentage of perivascular (F) and interstitial (G) collagen fiber, and number of infiltrated inflammatory cells (H) were determined as a measurement of fibrosis. DWN compounds and pirfenidone were administered at 10 mg/kg/day and 200 mg/kg/day, respectively ($n = 10$; One-way ANOVA; $^{***}P < 0.001$; mean ± SEM).

I *In vivo* toxicity of DWN10290 was determined in 2-week repeated dose toxicity study. HDW, hemoglobin concentration distribution width; RDW, red cell distribution width; MPV, mean platelet volume; MONO, monocyte; HGB, hemoglobin concentration; HCT, hematocrit; MCH, mean cell hemoglobin; MCHC, mean cell hemoglobin concentration; RBC, red blood cell; WBC, white blood cell; LYMP, lymphocyte; TBIL, total bilirubin; ALP, alkaline phosphatase; TG, triglyceride; HDL, high density lipoprotein; LDL, low density lipoprotein; CHO, cholesterol; BUN, blood urea nitrogen.

Source data are available online for this figure.

© 2023 The Authors

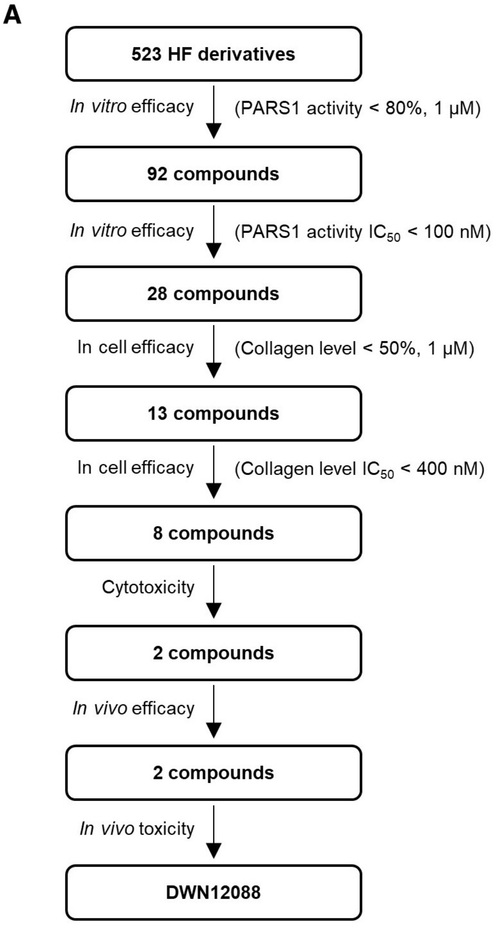

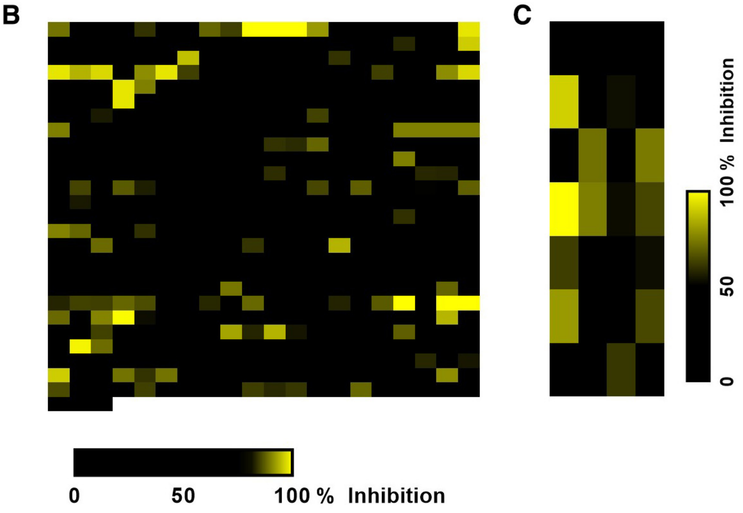

D

Compound	IC_{50} for collagen level (μM)	CC_{50} (μM)	CC_{50}/collagen IC_{50}
DWN10290	0.07398	77.05	1041
DWN10620	0.2150	29.28	136
DWN10993	0.04804	45.64	950
DWN12088	0.04307	> 100	> 2321
DWN12787	0.2745	> 100	> 364
DWN12923	0.2919	69.70	239
DWN12949	0.14750	> 100	> 678
DWN13012	0.10580	74.47	704

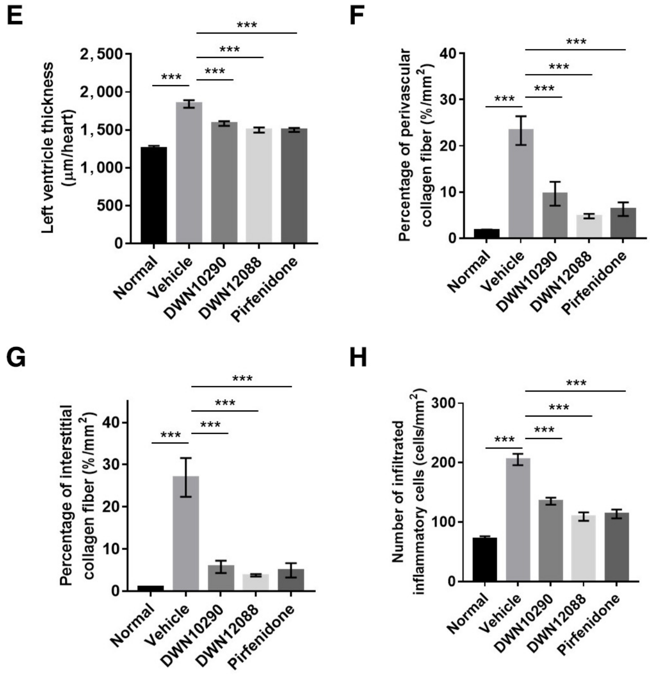

I

		DWN10290		
Dose level (mg/kg)		10	20	50
Mortality		-	-	Found dead: 1/5 (on day 9)
Clinical signs		-	Diarrhea	Diarrhea, Inanimation
Body weight		-	-	-
Hematology	↑	HDW	RDW, HDW	RDW, HDW, MPV, MONO
	↓	-	HGB, HCT, MCH, MCHC	RBC, HGB, HCT, WBC, LYMP
Clinical chemistry	↑	-	TBIL	ALP
	↓	HDL, LDL, CHO, BUN	TG, HDL, LDL, CHO, BUN	TG, HDL, LDL, CHO, BUN
Organ weight	↑	-	-	Liver, Kidney
	↓	-	-	Thymus
Necropsy finding		• Pale liver (4/5)	• Pale liver (1/5)	• Pale liver (4/5) • Spleen atrophy (1/5)

Figure EV2.

© 2023 The Authors

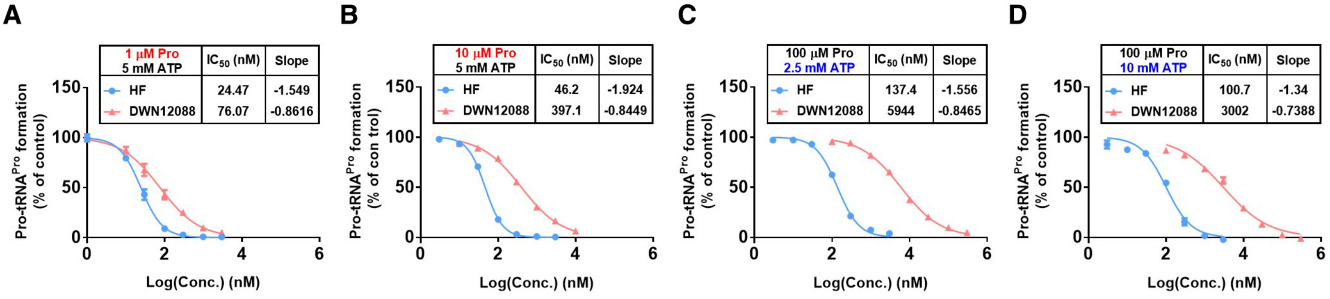

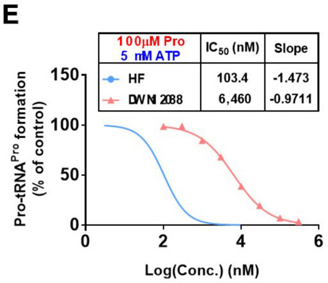

F

HF IC_{50} (nM)			
ATP (mM) \ Pro (µM)	1	10	100
2.5	-	-	137.4
5	24.47	46.2	103.4
10	-	-	100.7

G

DWN12088 IC_{50} (nM)			
ATP (mM) \ Pro (µM)	1	10	100
2.5	-	-	5,944
5	76.07	397.1	6,460
10	-	-	3,002

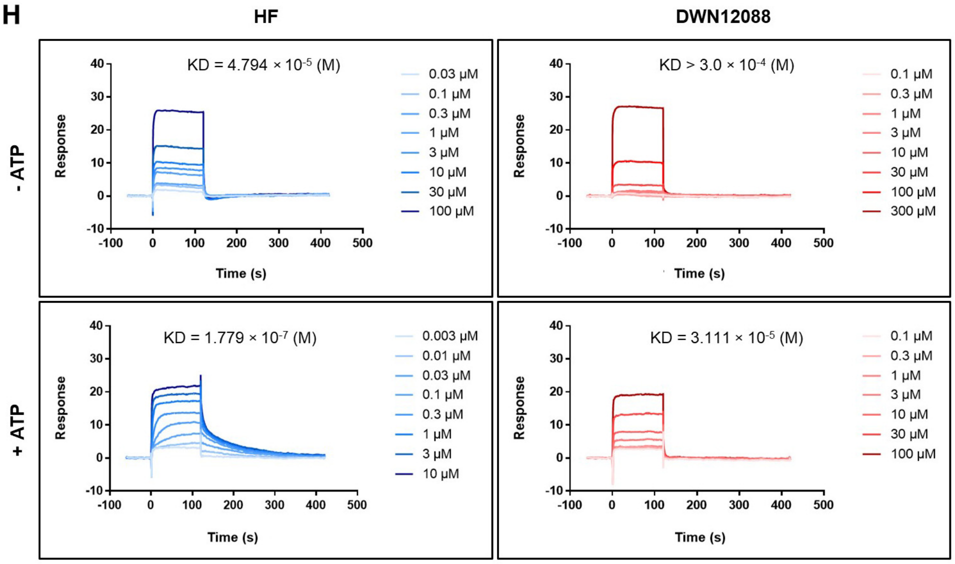

Figure EV3. Kinetic and binding properties of HF and DWN12088.

A–E The inhibitory activities of HF and DWN12088 were monitored by determining the catalytic activities of PARS1 under the indicated concentrations of proline and ATP. The IC_{50} values and slopes are listed in the table (A (HF), n = 5 from two independent experiments (triplicate for one experiment and duplicate for one experiment); A (DWN12088), n = 7 from three independent experiments (triplicate for one experiment and duplicate for two experiments); (B–D) technical replicate n = 3; (E) n = 9 from three independent experiments (triplicate for each experiment); mean ± SEM).

F, G IC_{50} values of HF and DWN12088 for *in vitro* prolylation assay were listed in the tables.

H The interaction of PARS1 and the indicated compounds in the presence or absence of ATP was determined via SPR. Affinity (KD) of the compound to PARS1 is shown on the top of the sensorgram.

Source data are available online for this figure.

Figure EV4. *In vivo* efficacy of DWN12088.

A–C The *in vivo* efficacy of DWN12088 was determined in a BLM-induced lung fibrosis model. The indicated compounds were administered a day before oropharyngeal administration of BLM. DWN12088 was administered at 3, 10, 30 mg/kg once a day and pirfenidone was administered at 100 mg/kg twice a day (200 mg/kg per day). After administering the compounds for 3 weeks, modified Ashcroft score (A) (Hubner *et al*, 2008), lung weight (B), and collagen content in BALF (C) were determined ($n = 10$; Mann–Whitney test after Kruskal–Wallis test; *$P < 0.05$, **$P < 0.01$; mean ± SEM). BLM, bleomycin; Pir, pirfenidone; NS, not significant.

D–F The *in vivo* efficacy of DWN12088, nintedanib and pirfenidone was compared in a BLM-induced lung fibrosis model. DWN12088 10 mg/kg, nintedanib 60 mg/kg and pirfenidone 200 mg/kg were orally administered to mice once a day from a week after intratracheal injection of BLM. After administering the compounds for 2 weeks, SpO_2 (D), BALF total cell count (E) and collagen contents (F) were determined ($n = 9$; Mann–Whitney test after Kruskal–Wallis test; *$P < 0.05$, **$P < 0.01$, ***$P < 0.001$; mean ± SEM). BLM, bleomycin; NS, not significant.

G–I The *in vivo* efficacy of HF and DWN12088 was compared in a BLM-induced lung fibrosis model. HF (0.05 and 0.1 mg/kg), and DWN12088 (10 mg/kg) were orally administered to mice once a day from 2 weeks after intratracheal injection of BLM. After administering for 2 weeks, SpO_2 (G), BALF total cell count (H), collagen contents (I) were determined ($n = 9$; Mann–Whitney test after Kruskal–Wallis test; mean ± SEM). BLM, bleomycin; 12088, DWN12088.

Source data are available online for this figure.

© 2023 The Authors

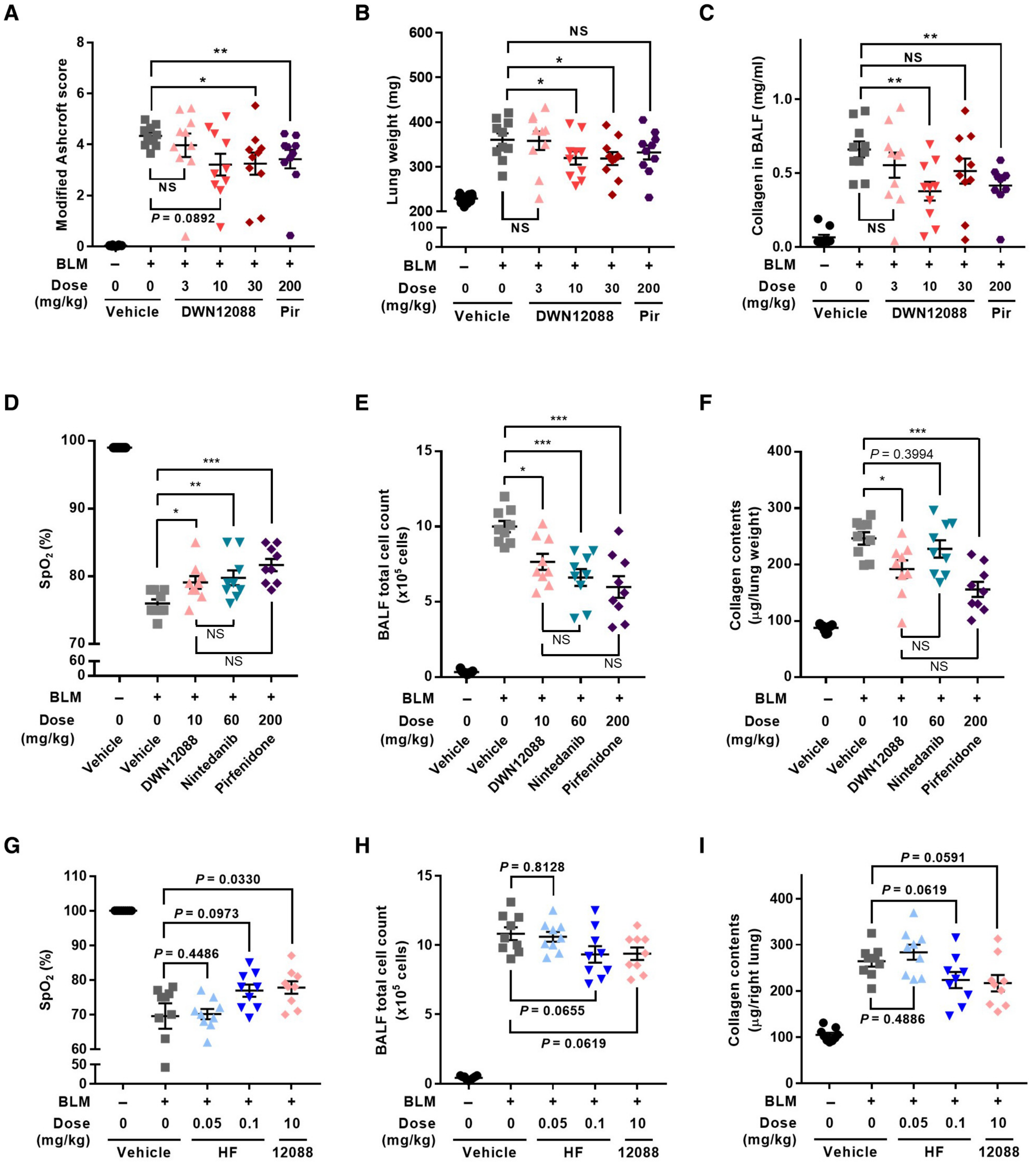
A
Modified Ashcroft score
BLM
Dose (mg/kg)
Vehicle
DWN12088
Pir
NS
P = 0.0892
B
Lung weight (mg)
C
Collagen in BALF (mg/ml)
D
SpO2 (%)
Vehicle
DWN12088
Nintedanib
Pirfenidone
E
BALF total cell count (x10^5 cells)
F
Collagen contents (µg/lung weight)
P = 0.3994
G
P = 0.0330
P = 0.0973
P = 0.4486
HF
12088
H
P = 0.8128
P = 0.0655
P = 0.0619
I
Collagen contents (µg/right lung)
P = 0.0591
P = 0.0619
P = 0.4886

Figure EV4.

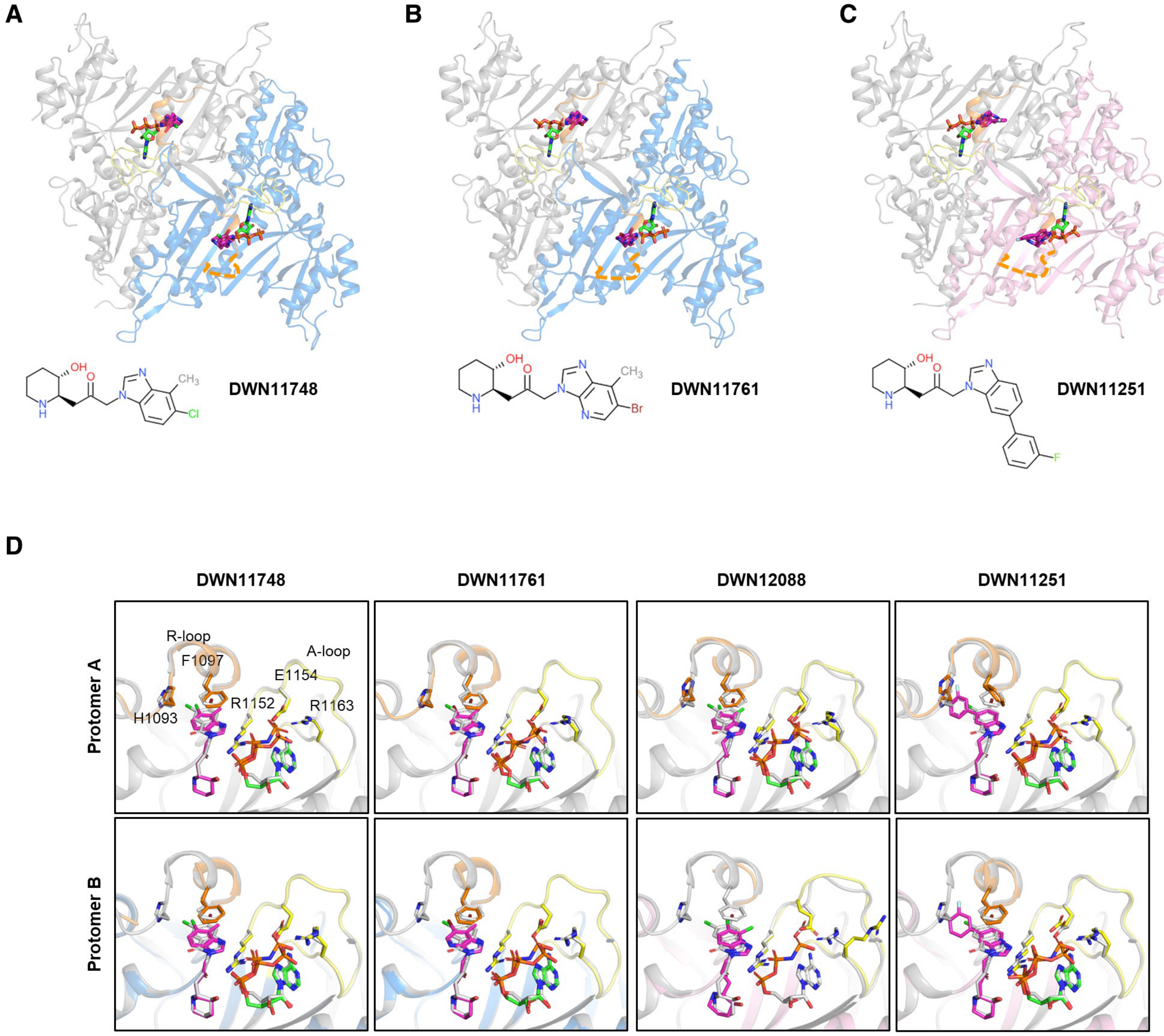

Figure EV5. The crystal structures of PARS1 complexed with DWN compounds.

A–C Overall structures of PARS1 complexed with the indicated compounds. PARS1 shows two types of the compound-binding states (rigid and loose binding). PARS1 chains with rigid binding are displayed in gray and blue whereas those with loose binding are displayed in pink. ATP is designated as green, and the compounds are designated as magenta stick models. R-loop (K1091 – F1097) and A-loop (R1152 – R1165) are presented as orange and yellow, respectively. The disordered region of R-loop is presented as orange dashed lines.

D Superposition of structures bound to DWN compounds and HF at the residues important for the interaction with compounds and ATP. DWN compound-bound structures are superposed with the F1097, R1152, E1154 and R1163 of 4HVC, which are represented as transparent gray stick models. The color codes of the structures are the same as those used in Fig 3A and B.

 © 2023 The Authors

Appendix for

Control of fibrosis with enhanced safety via asymmetric inhibition of prolyl-tRNA synthetase 1

Ina Yoon, Sulhee Kim, Minjae Cho, Kyung Ah You, Jonghyeon Son, Caroline Lee, Ji Hun Suh, Da-Jeong Bae, Jong Min Kim, Sinae Oh, Songhwa Park, Sanga Kim, Seong Hyeok Cho, Seonha Park, Kyuhyeon Bang, Minjeong Seo, Jong Hyun Kim, Bongyong Lee, Joon Seok Park, Kwang Yeon Hwang*, Sunghoon Kim*

*Correspondence to: chahong@korea.ac.kr (K.Y.H.), sunghoonkim@yonsei.ac.kr (S.K.)

Table of contents

Appendix Figure S1……………………………………………………………………….2

Appendix Figure S2……………………………………………………………………….3

Appendix Figure S3……………………………………………………………………….4

Appendix Figure S1

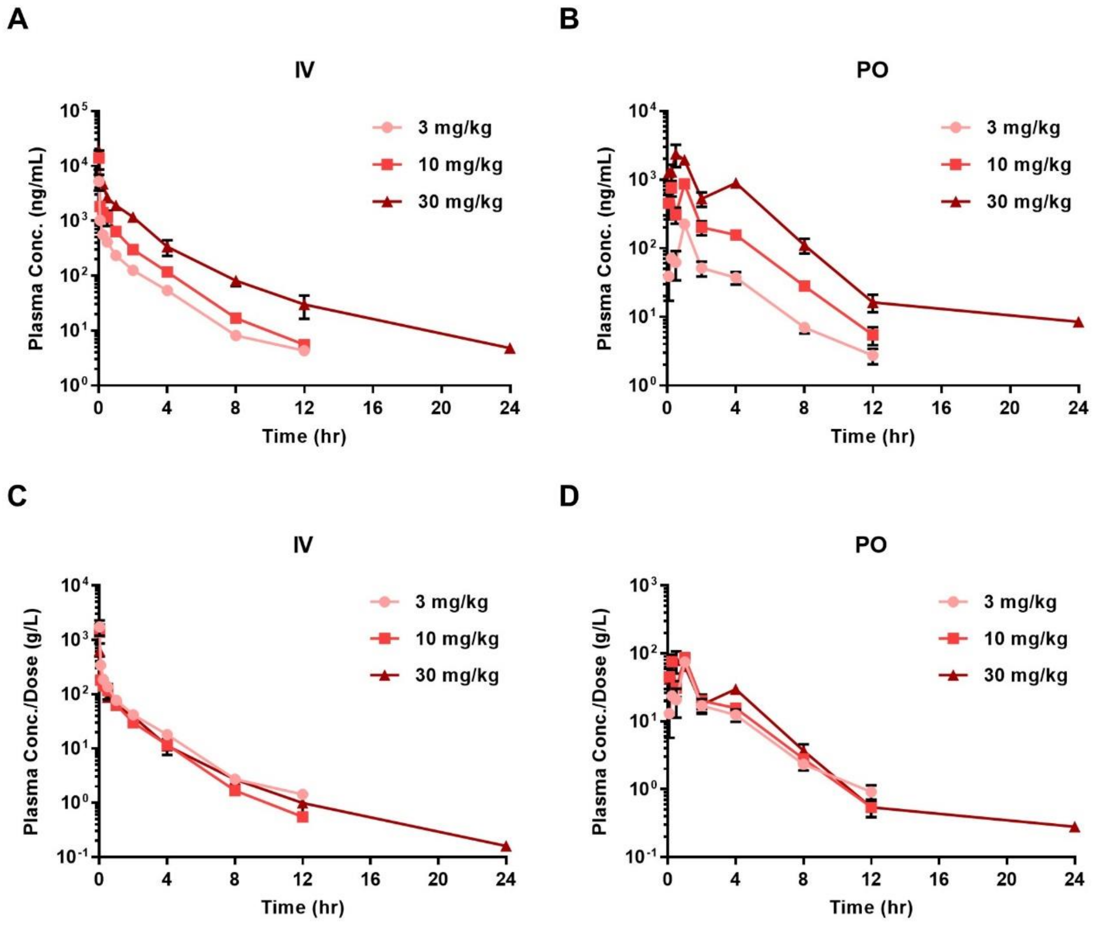

Appendix Fig S1. Pharmacokinetic study of DWN12088.

A, B. Plasma concentrations of DWN12088 at each time point were determined in mice intravenously (A) or orally (B) administered with the indicated dose of DWN12088 using LC-MS/MS (*n* = 3; mean ± SEM). IV, intravenous injection; PO, oral administration.

C, D. Dose-normalized plasma concentrations of DWN12088 at each time point were calculated based on (A) and (B).

Appendix Figure S2

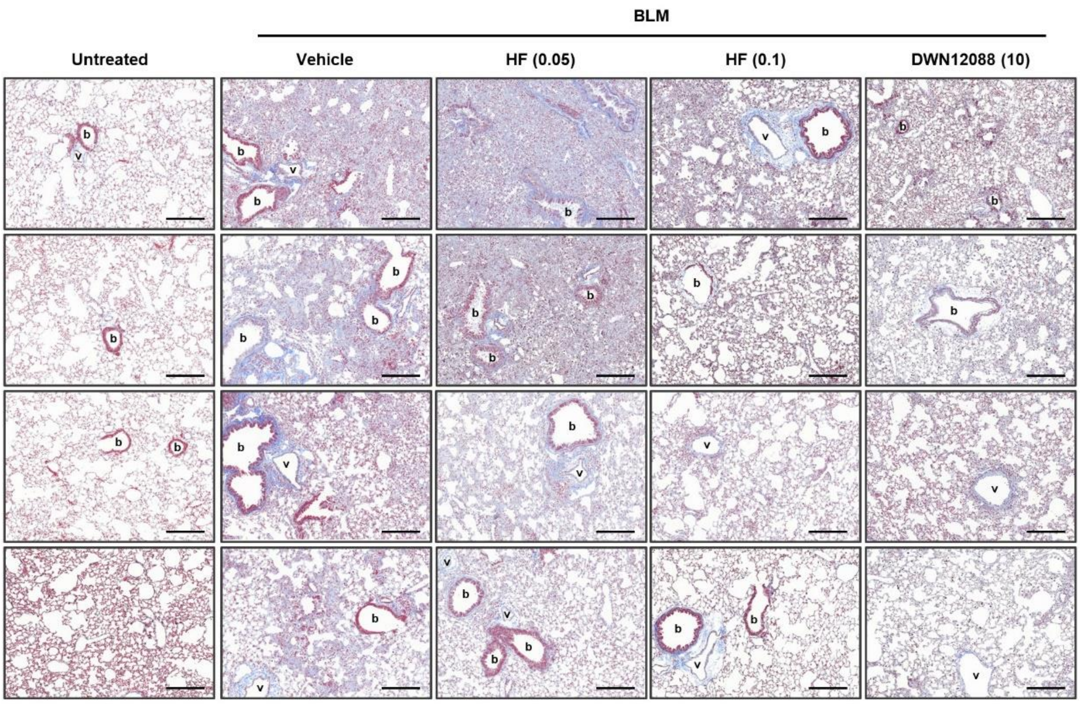

Appendix Fig S2. Comparison of *in vivo* efficacy of HF and DWN12088 in the collagen deposition of lung tissues obtained from the bleomycin-induced IPF model *in vivo*.

The *In vivo* efficacy of HF and DWN12088 was compared in a bleomycin-induced lung fibrosis model. The indicated concentrations of HF or DWN12088 were orally administered to mice once a day from a week after intratracheal injection of bleomycin. After administering the compounds for two weeks, collagen levels were determined by Masson's trichrome staining. Out of total five images, one is shown in Fig 1I (*n* = 1) and the rest are shown here (*n* = 4) (total, *n* = 5; scale bar = 200 μm). HF (0.05), HF 0.05 mg/kg; HF (0.1), HF 0.1 mg/kg; DWN12088 (10), DWN12088 10 mg/kg; BLM, bleomycin; b, bronchiole; v, blood vessel.

Appendix Figure S3

A

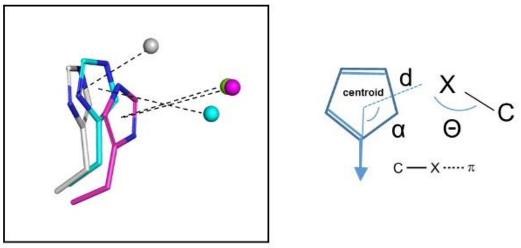

	Compound	d (Å)	α (°)	Θ (°)
Cl_green	HF Protomer A	3.36	96.0	110.9
Cl_magenta	HF Protomer B	3.36	92.1	115.7
Cl_cyan	DWN12088	4.26	81.2	98.9
F_gray	DWN11251	3.22	114.7	84.6

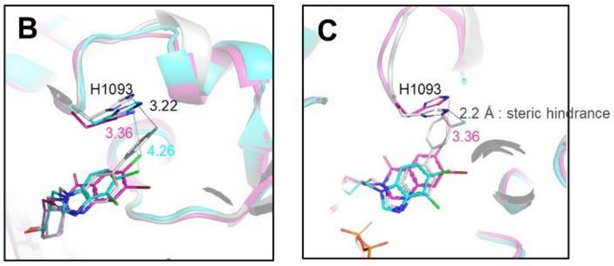

Appendix Fig S3. Comparison between H1093 and halogen atoms in each protomer.

A. Distance and angle between H1093 and halogen atoms of each compound. Various parameters related with halogen bond are summarized in the table.

B, C. Cartoon models near H1093 and halogen atoms of each compound. The individual protomers are superimposed. The color codes are same in (A). In protomer B, DWN compounds are predictive model when superimposed the ligands and model between HF and DWN compounds.
