# Supplementary material for: Control of fibrosis with enhanced safety via asymmetric inhibition of prolyl‐tRNA synthetase 1
Source: EMBO Mol Med. 2023 May 22;15(7):e16940. doi: 10.15252/emmm.202216940 (PMC10331583; doi:10.15252/emmm.202216940)
Supplement: Supplementary file 8 — Source Data for Figure 5 [file EMMM-15-e16940-s009.zip › EMM-2022-16940-Figure_3_Source_Data.pdf]

Source Data

Scheme 1. Synthesis of compound 5 (DWN12088)

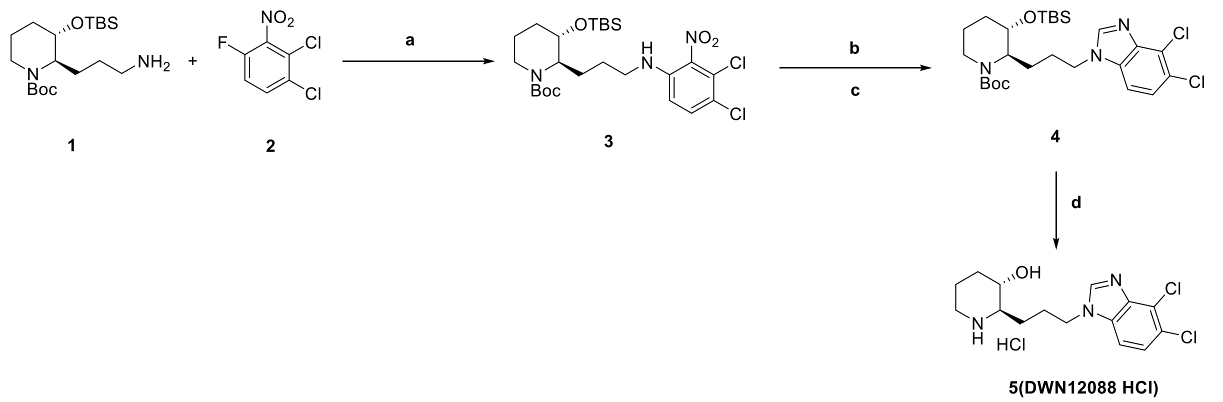

[a]***Reagents and Conditions:*** a) N,N-diisopropylethylamine (DIPEA), trifluoroacetic acid (THF), reflux; b) sodium dithionite ($Na_2S_2O_4$), potassium carbonate (K_2CO_3), ethanol (EtOH), H_2O; c) trimethyl orthoformate (TMOF), p-Toluenesulfonic acid (PTSA), toluene; d) i) concentrated Hydrochloric acid (c-HCl), ethyl acetate (EtOAc), ii) sodium hydroxide (NaOH), H_2O, methylene chloride (DCM), iii) c-HCl, Acetone/H_2O

tert-butyl (2R, 3S)-3-((tert-butyldimethylsilyl)oxy)-2-(3-((3,4-dichloro-2-nitrophenyl)amino)propyl)piperidine-1-carboxylate (3)

Commercially available compound tert-butyl (2R,3S)-2-(3-aminopropyl)-3-((tert-butyldimethylsilyl)oxy)piperidine-1-carboxylate (CAS : 2241812-39-3, 100.0 g, 0.27 mol, 1.0 eq) was dissolved in tetrahydrofuran 1000 mL (10 volumes) and 1,2-dichloro-4-fluoro-3-nitrobenzene (56.4 g, 0.27 mol, 1.0 eq) and N,N-Diisopropylethylamine (93.5 mL, 0.54 mol, 1.0 eq) were added. The mixture was stirred for seven hours at reflux (External Temperature: 80°C). Using in process control (IPC), the reaction was checked until 1,2-dichloro-4-fluoro-3-nitrobenzene became 2% or less. Then, the termination of the reaction was checked. The organic layers were extracted by adding 1000 mL of purified water (10 volumes). Re-extraction

was carried out by adding 1000 mL of ethyl acetate (10 volumes). Following vacuum evaporation, the residue was re-evaporated by adding 100 mL of ethanol (1 volume). The residue was dissolved in 400 mL of ethanol (4 volumes) at 40 °C and cooled down for crystallization (seeding administered if crystals are not formed). Crystallization was carried out for two hours at 0 - 5 °C by adding 400 mL of purified water (4 volumes). The mixture was vacuum-filtered and washed with a mixture of 100 mL each of ethanol and purified water to obtain 133.0 g of a labeled compound **3** as an orange solid (Yield: 88%). ^{1}H-NMR (500 MHz, CD_3OD) δ 7.43 (d, J = 9.0 Hz, 1H), 6.88 (d, J = 9.0 Hz, 1H), 4.08 (brs, 1H), 3.94 (brs, 1H), 3.76 (d, J = 1.5 Hz, 1H), 3.23 (m, 2H), 2.76 (brs, 1H), 1.83 (m, 1H), 1.74 (m, 2H), 1.58 (m, 3H), 1.41 (s, 10H), 1.32 (m, 1H), 0.89 (s, 9H), 0.07 (d, J = 10.5 Hz, 6H); HRMS (FAB+) m/z calcd for $C_{25}H_{42}Cl_2N_3O_5Si$ $[M+H]^+$ 562.2271, found 562.2280.

tert-butyl (2R, 3S)-3-((tert-butyldimethylsilyl)oxy)-2-(3-(4,5-dichloro-1H-benzo[d]imidazol-1-yl)propyl)piperidine-1-carboxylat (4)

tert-butyl (2R,3S)-3-((tert-butyldimethylsilyl)oxy)-2-(3-((3,4-dichloro-2-nitrophenyl) amino)propyl)piperidine-1-carboxylate (120.0 g, 0.21 mol, 1.0 eq) was dissolved in 1200 mL of ethanol (10 volumes) and potassium carbonate (117.8 g, 0.85 mol, 4.0 eq) and 1100 mL of a sodium dithionite solution (148.7 g, 2.7 mol, 4.0 eq) were added. The mixture was stirred for one hour at room temperature. Using IPC, the reaction was checked until Nitrophenyl compound **3** became 1% or less. Then, the termination of the reaction was checked. After vacuum-evaporating ethanol, extraction was carried out by adding 600 mL of purified water (5 volumes) and 1200 mL of ethyl acetate (10 volumes). Re-extraction was carried out by adding 600 mL of ethyl acetate (5 volumes). The organic layers were washed using 1200 mL of brine (10 volumes). The remaining moisture was removed by adding sodium sulfate. Through vacuum evaporation, 113.6 g of a compound was obtained as a brown liquid. The residue (113.6

g, 0.21 mol, 1.0 eq) was dissolved in 1136 mL of toluene (10 volumes) and trimethyl orthoformate (70.0 mL, 0.64 mol, 3.0 eq) and p-toluenesulfonic acid (0.4 g, 0.02 mol, 0.1 eq) were added. The mixture was stirred for one hour at 50 - 60 °C. Using IPC, the reaction was checked until intermediate aniline compound became 2% or less. Then, the termination of the reaction was checked. The 1136 mL of toluene used in the reaction was removed through vacuum evaporation. Extraction was carried out by adding 122 mL of a sodium bicarbonate solution, 1136 mL of ethyl acetate (10 volumes) and 1136 mL of purified water (10 volumes) (successfully stratified). Re-extraction was carried out by adding 568 mL of ethyl acetate (5 volumes). The remaining moisture was removed by adding sodium sulfate and the color was removed through Celite filtering using KB-B (11.4 g, 0.1 eq). After vacuum-evaporating the filtrate, 227 mL of hexane (2 volumes) was added to the residue. The mixture was vacuum-evaporated and then crystallized for two hours at 0 °C by adding 340 mL of hexane (3 volumes). The mixture was vacuum-filtered and washed with 113 mL of hexane (1 volume) to obtain 100 g of a labeled compound **4** as a white solid (Yield: 86%). ^{1}H-NMR (500 MHz, CD_3OD) δ 8.28 (s, 1H), 7.54 (d, J = 8.5 Hz, 1H), 7.41 (d, J = 8.5 Hz, 1H), 4.32 (m, 2H), 4.16 (m, 1H), 3.92 (d, J = 11.0 Hz, 1H), 3.72 (s, 1H), 2.67 (brs, 1H), 1.86 (m, 3H), 1.66 (m, 2H), 1.55 (m, 1H), 1.40 (m, 10H), 1.27 (m, 1H), 0.87 (s, 9H), 0.05 (d, J = 13.0 Hz, 6H); HRMS (FAB+) m/z calcd for $C_{26}H_{42}Cl_2N_3O_3Si$ $[M+H]^+$ 542.2373, found 542.2381

(2R, 3S)-2-(3-(4,5-dichloro-1H-benzo[d]imidazol-1-yl)propyl) piperidin-3-ol HCl (5, DWN12088)

tert-butyl (2R,3S)-3-((tert-butyldimethylsilyl)oxy)-2-(3-(4,5-dichloro-1H-benzo[d] imidazol-1-yl)propyl)piperidine-1-carboxylate (90.0 g, 0.17 mol, 1.0 eq) was dissolved in 540 mL of ethyl acetate (6 volumes) and stirred at 5 °C. 35% HCl (146.4 mL, 10.0 eq) was added and the

mixture was stirred for one hour. Using IPC, the reaction was checked until compound **4** became 0.2% or less. Then, the termination of the reaction was checked. Extraction was carried out by adding 540 mL of purified water (6 volumes). Re-extraction was carried out by adding 540 mL of ethyl acetate (6 volumes) to the water layer. The organic layer (ethyl acetate) was discarded and the pH was adjusted to 12.5 or higher by adding 8N sodium hydroxide solution. Extraction was carried out by adding 900 mL of methylene chloride (10 volumes). Re-extraction was carried out by adding 450 mL of methylene chloride (5 volumes). Following vacuum evaporation, the residue was dissolved in 1100 mL of acetone (20 volumes) and 54 mL of purified water (2 volumes). After adding hydrochloric acid (pH: 6.5 - 7) by 35% of one volume, the mixture was stirred for two hours at 5 °Cfor crystallization. The mixture was vacuum-filtered to obtain 58.5 g of a labeled compound **5** as a purified white solid (Yield: 97%). ^{1}H-NMR (500Mz, DMSO-d_6) δ 9.02 (brs, 1H), 8.45 (s, 1H), 7.72 (d, J = 8.5 Hz, 1H), 7.48 (d, J = 8.5 Hz, 1H), 5.40 (d, J = 5.5 Hz, 1H), 4.31 (m, 2H), 3.41 (m, 1H), 3.09 (d, J = 12 Hz, 1H), 2.77 (m, 2H), 2.08 (m, 1H), 1.97 (m, 1H), 1.87 (m, 2H), 1.75 (m, 1H), 1.66 (m, 1H), 1.53 (m, 1H), 1.36 (m, 1H); ^{13}C-NMR (125 MHz, DMSO-d_6) δ 146.1, 141.5, 133.4, 124.3, 123.7, 121.4, 110.9, 67.0, 59.8, 44.3, 43.1, 31.4, 26.1, 25.3, 20.3; HRMS (FAB+) m/z calcd for $C_{15}H_{20}Cl_2N_3O$ [M+H]$^+$ 328.0983, found 328.0988. purity >99.9% (as determined by RP-HPLC, R_t = 15.96 min)

1-(6-(3-fluorophenyl)-1*H*-benzo[d]imidzol-1-yl)-3-((2R,3S)-3-hydroxypiperidin-2-yl)propan-2-one (DWN11251)

^{1}H-NMR (500Mz, CD_3OD) δ 8.11 (s, 1H), 7.52 (d, J = 7.5 Hz, 2H), 7.58 (d, J = 8.5 Hz, 1H), 7.51 (d, J = 7.5 Hz, 1H), 7.44 (m, 2H), 7.06 (m, 1H), 4.82 (s, 2H), 3.23 (td, J = 10.5, 4.0 Hz, 1H), 3.12 (dd, J = 16.5, 5.0 Hz, 1H), 2.88 (m, 2H), 2.58 (m, 2H), 2.05(m, 1H), 1.75 (m, 1H),

1.53 (m, 1H), 1.37 (m, 1H); ^{13}C-NMR (125 MHz, CD_3OD) δ 203.1, 16.2, 162.2, 145.0, 143.8, 142.0, 135.4, 130.1, 122.8. 121.6, 118.9, 113.7, 113.5, 113.1, 108.7, 71.2, 59.4, 59.3, 45.3, 33.5, 24.8

1-(5-chloro-4-methyl-1H-benzo[d]imidazol-1-yl)-3-((2R,3S)-3-hydroxypiperidin-2-yl)propan-2-one (DWN11748)

^{1}H-NMR (500Mz, CD_3OD) δ 8.05(s, 1H), 7.22 (S, 2H), 4.82 (s, 2H), 3.20(td, J = 10.5, 4.5 Hz, 1H), 3.05 (dd, J = 16.0, 5.0Hz, 1H), 2.92 (d, J = 12.5 Hz, 1H), 2.83 (m, 1H), 2.60 (s, 3H), 2.51 (m, 2H), 2.04 (m, 1H), 1.72 (m, 1H), 1.52 (m, 1H), 1.37(m, 1H); ^{13}C-NMR (125 MHz, CD_3OD) δ 202.9, 144.6, 142.4, 132.5, 127.3, 126.6, 123.7, 108.5, 71.4, 71.3, 59.4, 59.3, 45.3, 33.5, 24.9, 12.7

1-(6-bromo-7-methyl-3H-imidazo[4,5-b]pyridin-3-yl)-3-((2R,3S)-3-hydroxypiperidin-2-yl)propan-2-one (DWN11761)

^{1}H-NMR (500Mz, CD_3OD) δ 8.32(s, 1H), 8.23 (s, 1H), 4.82 (s, 2H), 3.20 (td, J = 10.5, 4.5 Hz, 1H), 2.91 (d, J = 12.0 Hz, 1H), 2.82 (m, 1H), 2.63 (s, 3H), 2.62 (m, 1), 2.52 (m, 2H), 2.04 (d, J = 9.0 Hz, 1H), 1.71 (d, J = 13.0 Hz, 1H), 1.50 (m, 1H), 1.31 (m, 1H); ^{13}C-NMR (125 MHz, CD_3OD) δ 202.8, 145.9, 145.1, 144.9, 138.8, 134.7, 116.3, 70.8, 70.7, 59.4, 59.3, 45.3, 33.6, 24.8, 15.3
