# Supplementary material for: Control of fibrosis with enhanced safety via asymmetric inhibition of prolyl‐tRNA synthetase 1
Source: EMBO Mol Med. 2023 May 22;15(7):e16940. doi: 10.15252/emmm.202216940 (PMC10331583; doi:10.15252/emmm.202216940)
Supplement: Supplementary file 10 [file EMMM-15-e16940-s007.pdf]

EMBO Molecular Medicine

Control of fibrosis with enhanced safety via asymmetric inhibition of prolyl-tRNA synthetase 1

Ina Yoon, Sulhee Kim, Minjae Cho, Kyung Ah You, Jonghyeon Son, Caroline Lee, Ji Hun Suh, Da-Jeong Bae, Jong Min Kim, Sinae Oh, Songhwa Park, Sanga Kim, Seong Hyeok Cho, Seonha Park, Kyuhyeon Bang, Minjeong Seo, Jong Hyun Kim, Bong Yong Lee, Joon Seok Park, Kwang Yeon Hwang, and Sunghoon Kim
DOI: 10.15252/emmm.202216940

Corresponding author: Sunghoon Kim (sunghoonkim@yonsei.ac.kr) and Kwang Yeon Hwang (chahong@korea.ac.kr)

Review Timeline:

Submission Date:	25th Sep 22
Editorial Decision:	31st Oct 22
Revision Received:	12th Apr 23
Editorial Decision:	26th Apr 23
Revision Received:	3rd May 23
Accepted:	3rd May 23

Editor: Zeljko Durdevic

Transaction Report:

(Note: With the exception of the correction of typographical or spelling errors that could be a source of ambiguity, letters and reports are not edited. Depending on transfer agreements, referee reports obtained elsewhere may or may not be included in this compilation. Referee reports are anonymous unless the Referee chooses to sign their reports.)

31st Oct 2022

Dear Prof. Kim,

Thank you for the submission of your manuscript to EMBO Molecular Medicine. We have now received feedback from the three reviewers who agreed to evaluate your manuscript. As you will see from the reports, all three referees recognize potential interest of the study but also raise serious concerns that should be addressed in a major revision. Considering that the revision will require additional experimentations in mice we think six months would be more appropriate to provide the complete revision.

Further consideration of a revision that addresses reviewers' concerns in full will entail a second round of review. EMBO Molecular Medicine encourages a single round of revision only and therefore, acceptance or rejection of the manuscript will depend on the completeness of your responses included in the next, final version of the manuscript. For this reason, and to save you from any frustrations in the end, I would strongly advise against returning an incomplete revision.

We would welcome the submission of a revised version within six months for further consideration. Please let us know if you require longer to complete the revision.

I look forward to receiving your revised manuscript.

Yours sincerely,

Zeljko Durdevic

Zeljko Durdevic
Editor
EMBO Molecular Medicine

*****

When submitting your revised manuscript, please carefully review the instructions that follow below. We perform an initial quality control of all revised manuscripts before re-review; failure to include requested items will delay the evaluation of your revision.

We require:

1) A .docx formatted version of the manuscript text (including legends for main figures, EV figures and tables). Please make sure that the changes are highlighted to be clearly visible.

2) Individual production quality figure files as .eps, .tif, .jpg (one file per figure). For guidance, download the 'Figure Guide PDF': (https://www.embopress.org/page/journal/17574684/authorguide#figureformat).

3) A .docx formatted letter INCLUDING the reviewers' reports and your detailed point-by-point responses to their comments. As part of the EMBO Press transparent editorial process, the point-by-point response is part of the Review Process File (RPF), which will be published alongside your paper.

4) A complete author checklist, which you can download from our author guidelines (https://www.embopress.org/page/journal/17574684/authorguide#submissionofrevisions). Please insert information in the checklist that is also reflected in the manuscript. The completed author checklist will also be part of the RPF.

5) Please note that all corresponding authors are required to supply an ORCID ID for their name upon submission of a revised manuscript.

6) It is mandatory to include a 'Data Availability' section after the Materials and Methods. Before submitting your revision, primary datasets produced in this study need to be deposited in an appropriate public database, and the accession numbers and

database listed under 'Data Availability'. Please remember to provide a reviewer password if the datasets are not yet public (see https://www.embopress.org/page/journal/17574684/authorguide#dataavailability).

In case you have no data that requires deposition in a public database, please state so in this section. Note that the Data Availability Section is restricted to new primary data that are part of this study.

7) For data quantification: please specify the name of the statistical test used to generate error bars and P values, the number (n) of independent experiments (specify technical or biological replicates) underlying each data point and the test used to calculate p-values in each figure legend. The figure legends should contain a basic description of n, P and the test applied. Graphs must include a description of the bars and the error bars (s.d., s.e.m.). See also 'Figure Legend' guidelines: https://www.embopress.org/page/journal/17574684/authorguide#figureformat

8) At EMBO Press we ask authors to provide source data for the main manuscript figures. Our source data coordinator will contact you to discuss which figure panels we would need source data for and will also provide you with helpful tips on how to upload and organize the files.

9) Our journal encourages inclusion of *data citations in the reference list* to directly cite datasets that were re-used and obtained from public databases. Data citations in the article text are distinct from normal bibliographical citations and should directly link to the database records from which the data can be accessed. In the main text, data citations are formatted as follows: "Data ref: Smith et al, 2001" or "Data ref: NCBI Sequence Read Archive PRJNA342805, 2017". In the Reference list, data citations must be labeled with "[DATASET]". A data reference must provide the database name, accession number/identifiers and a resolvable link to the landing page from which the data can be accessed at the end of the reference. Further instructions are available at .

10) We replaced Supplementary Information with Expanded View (EV) Figures and Tables that are collapsible/expandable online. A maximum of 5 EV Figures can be typeset. EV Figures should be cited as 'Figure EV1, Figure EV2" etc... in the text and their respective legends should be included in the main text after the legends of regular figures.

- For the figures that you do NOT wish to display as Expanded View figures, they should be bundled together with their legends in a single PDF file called *Appendix*, which should start with a short Table of Content. Appendix figures should be referred to in the main text as: "Appendix Figure S1, Appendix Figure S2" etc.

- Additional Tables/Datasets should be labeled and referred to as Table EV1, Dataset EV1, etc. Legends have to be provided in a separate tab in case of .xls files. Alternatively, the legend can be supplied as a separate text file (README) and zipped together with the Table/Dataset file.

See detailed instructions here:

.

11) The paper explained: EMBO Molecular Medicine articles are accompanied by a summary of the articles to emphasize the major findings in the paper and their medical implications for the non-specialist reader. Please provide a draft summary of your article highlighting

- the medical issue you are addressing,

- the results obtained and

- their clinical impact.

This may be edited to ensure that readers understand the significance and context of the research. Please refer to any of our published articles for an example.

12) For more information: There is space at the end of each article to list relevant web links for further consultation by our readers. Could you identify some relevant ones and provide such information as well? Some examples are patient associations, relevant databases, OMIM/proteins/genes links, author's websites, etc...

13) Author contributions: You will be asked to provide CRediT (Contributor Role Taxonomy) terms in the submission system. These replace a narrative author contribution section in the manuscript.

14) A Conflict of Interest statement should be provided in the main text.

15) Every published paper now includes a 'Synopsis' to further enhance discoverability. Synopses are displayed on the journal

webpage and are freely accessible to all readers. They include a short stand first (maximum of 300 characters, including space) as well as 2-5 one-sentences bullet points that summarizes the paper. Please write the bullet points to summarize the key NEW findings. They should be designed to be complementary to the abstract - i.e. not repeat the same text. We encourage inclusion of key acronyms and quantitative information (maximum of 30 words / bullet point). Please use the passive voice. Please attach these in a separate file or send them by email, we will incorporate them accordingly.

Please also suggest a striking image or visual abstract to illustrate your article as a PNG file 550 px wide x 300-600 px high.

EMBO Molecular Medicine has a "scooping protection" policy, whereby similar findings that are published by others during review or revision are not a criterion for rejection. Should you decide to submit a revised version, I do ask that you get in touch after three months if you have not completed it, to update us on the status.

Please note: When submitting your revision you will be prompted to enter your funding and payment information. This will allow Wiley to send you a quote for the article processing charge (APC) in case of acceptance. This quote takes into account any reduction or fee waivers that you may be eligible for. Authors do not need to pay any fees before their manuscript is accepted and transferred to the publisher.

EMBO Press participates in many Publish and Read agreements that allow authors to publish Open Access with reduced/no publication charges. Check your eligibility: https://authorservices.wiley.com/author-resources/Journal-Authors/open-access/affiliation-policies-payments/index.html

***** Reviewer's comments *****

Referee #1 (Comments on Novelty/Model System for Author):

Technicality: This is a very through and competent piece of work which employs a large number of techniques to produce high quality data carried out by research groups with great expertise in the fields encompassed in this study. The study employs these advanced techniques to reveals that it is possible to increase the Safety Window of a promising modified prolyl-tRNA synthesise inhibitor (DWN12088) when used as a treatment of Idiopathic pulmonary fibrosis.

Novelty and Medical Impact: The work presented here involves the screening of a chemically library of drugs based on the molecular scaffold of Halgofuginone, a known Prolyl-tRNA synthetase inhibitor, to improve the efficacy and safety of a drug to control fibrosis. The most promising drug variant discovered, DWN12088, does indeed shows a significant increase in its Safety window, and therefore greatly enhances prolyl-tRNA syntheses inhibitors as prominent leads in combating fibrosis. In addition, the novel asymmetric inhibition mechanism employed by DWN12088 to improve its Therapeutic Index also suggests that other tRNA synthesise inhibitors maybe developed in a similar manner to create other promising drugs against analogous diseases. By its very nature, an article improving an existing inhibitor somewhat suffers on the Novelty criteria compared to say a paper describing the the discovery of a new inhibitor class. However, this unavoidable cost on Novelty in this study is more than compensated by the Medical Impact of findings that identify a very promising drug with improved Therapeutic Index to combat IPF - a disease which suffers from a lack of treatments available.

Referee #1 (Remarks for Author):

Major points:
(a) Figure 1 (H) Masson's trichrome staining of lung tissue appears to a lack any quantitation? The analysis should also look in more detail at collagen deposition around specific lung tissue structures such as airways and vessels in the images collected.
(b)The paper would greatly benefit from a more thorough steady state kinetic analysis of the aminoacylation reaction of PARS1 wild-type and mutant FA/EA/RL in both homo and heteromeric settings instead of simple prolylation assays looking at single time points (Fig 6c). Active site measurements of the homo and heteromeric PARS1 dimer activities might also help solidify the model outlined in Fig 6E.
(c) Have more through Ki determination studies been performed on PARS1 aminoacylation activity in the presence of DWN12088 inhibitor?

Minor points:
(a) The article, especially in the Introduction and Discussion, lacks detailed description/references on the improvement of the Therapeutic Index for other compounds targeting different diseases. This is important so the reader can judge how significant the improvement of the Safety Window of DWN12088 over Halgofuginone actually is.
(b) Is there anyway that the large number very similar panels in Figures 3 and 4 can be reduced/ rationalized/ partially moved to supplementary work section to make it easier on the reader to follow the work?

Referee #2 (Comments on Novelty/Model System for Author):

2. glutamyl-propyl-tRNA synthase is a established target in the context of pulmonary fibrosis since 2019.

4. Therapeutic, rather than the applied prophylactic, administration and/or the use of the Ad-TGFmodel, would streng results

Referee #2 (Remarks for Author):

Authors present in this paper a success story for controlling fibrosis through developing small molecule prolyl-tRNA synthetase 1 inhibitors endowed with a desired safety profile and a new binding mode (assymetric catalytic inhibitor). The authors went through a well-organized and conducted scrutiny, including medicinal chemistry, structural biology, in vitro, cell-based, cytotoxicity as well as in vivo bioefficacy and toxicity studies. Compound HF was used as a starting point, which suffers from serious toxicity issues. This scrutiny rendered them able to refine all those molecular determinants providing both potency (in vitro and in vivo) and drug-like properties at the same time, giving rise to compound DWN12088, a clinical candidate currently being found in phase II trials. Of note, the improved therapeutic index (efficacy/toxicity) of DWN12088 in IPF models has been achieved, at least in part, through the unique mode of action of this compound. This includes DWN12088's binding to PARS1 homodimer in an asymmetric manner, thus inhibiting enzyme's two catalytic sites differently compared to its precursor HF.

There are some questions remaining to be addressed and which would strengthen the study:
1) Both 1H-NMR and 13C-NMR at least of the compounds displayed in Figure 3 must be provided in the supporting information file.
2) Under which criteria DWN2088 was administered in vivo at 10 mg/kg? Was a pharmacokinetic study performed first? Or the dose of administration was selected randomly?
3) In the in vivo experiment using the bleomycin mouse model, DWN2088's activity was evaluated at a prophylactic mode. The strength of the in vivo study as well as the experimental findings could be enhanced by testing compound's activity at a therapeutic mode (administration of the compound after fibrosis establishment). Given that the majority of drugs against fibroproliferative diseases are prescribed and given at late stages of the disease, evaluating the impact of DWN2088 on IPF after disease establishment would mean a lot about the therapeutic potential of this novel series of asymmetric inhibitors of prolyl-tRNA synthetase 1.
4) an additional fibrosis model, like Ad-TGFβ, would also strengthen findings, especially since it bypasses the inflammatory stage of BLM.

Referee #3 (Remarks for Author):

Prolyl-tRNA synthetase 1 (PARS1) controls pathogenic accumulation of collagen in fibrotic diseases.
PARS1 regulates proline synthesis and can be a target for fibrotic diseased, however, regulation of proline synthesis may cause adverse effects on global protein synthesis. The authors validated that PARS1 worked as a positive regulator of collagen levels a positive regulator of collagen levels and developed a novel compound, DWN12088, which can be a candidate of therapeutic agent against fibrosis. The authors also showed binding mode of DWN12088 in the crystal structures.
The paper is well written, and the results are clear to discuss. I, therefore, recommend the manuscript will be accepted with small minor modifications.

Minor points to be corrected.
The authors should describe mode detail of X-ray diffraction data collection; such as beamline name, detector name,

In the crystallographic data table,

Space group P 1 2(1) 1 can be P2(1).
Cell angle, alpha and gamma can be omitted, since these values are evident from the space group.
"I" should be italic in I/sigmaI

Line 691
Reference of coot should be Emsley et al. 2010. See,
https://www2.mrc-lmb.cam.ac.uk/personal/pemsley/coot/docs/coot-faq.html#Citing

Referee #1 (Comments on Novelty/Model System for Author):

Technicality: This is a very through and competent piece of work which employs a large number of techniques to produce high quality data carried out by research groups with great expertise in the fields encompassed in this study. The study employs these advanced techniques to reveals that it is possible to increase the Safety Window of a promising modified prolyl-tRNA synthesise inhibitor (DWN12088) when used as a treatment of Idiopathic pulmonary fibrosis.

Novelty and Medical Impact: The work presented here involves the screening of a chemically library of drugs based on the molecular scaffold of Halgofuginone, a known Prolyl-tRNA synthetase inhibitor, to improve the efficacy and safety of a drug to control fibrosis. The most promising drug variant discovered, DWN12088, does indeed shows a significant increase in its Safety window, and therefore greatly enhances prolyl-tRNA syntheses inhibitors as prominent leads in combating fibrosis. In addition, the novel asymmetric inhibition mechanism employed by DWN12088 to improve its Therapeutic Index also suggests that other tRNA synthesise inhibitors maybe developed in a similar manner to create other promising drugs against analogous diseases.

By its very nature, an article improving an existing inhibitor somewhat suffers on the Novelty criteria compared to say a paper describing the discovery of a new inhibitor class. However, this unavoidable cost on Novelty in this study is more than compensated by the Medical Impact of findings that identify a very promising drug with improved Therapeutic Index to combat IPF - a disease which suffers from a lack

of treatments available.

Referee #1 (Remarks for Author):

Major points:

(a) Figure 1 (H) Masson's trichrome staining of lung tissue appears to a lack any quantitation? The analysis should also look in more detail at collagen deposition around specific lung tissue structures such as airways and vessels in the images collected.

Answer:

As the reviewer suggested, we quantified fibrotic area of the Masson's trichrome staining images and included the results in Fig. 1H of the revised manuscript. In addition, we marked bronchioles and vessels in the images and provided Masson's trichrome staining results of the remaining four mice in Appendix Fig S2. Moreover, we added a description of collagen deposition around specific lung tissue structures to the revised manuscript (Line 205-208).

(b)The paper would greatly benefit from a more thorough steady state kinetic analysis of the aminoacylation reaction of PARS1 wild-type and mutant FA/EA/RL in both homo and heteromeric settings instead of simple prolylation assays looking at single time points (Fig 6c). Active site measurements of the homo and heteromeric PARS1 dimer activities might also help solidify the model outlined in Fig 6E.

Answer:

We performed experiments for kinetic analysis of PARS1 aminoacylation reaction in

homodimer (PARS1 wild-type, WT/WT) and heterodimer (PARS1 wild-type and mutant FA/EA/RL, WT/MT) settings (Fig. 6D). The heterodimer PARS1 showed an increased K_m value for both proline and ATP compared to homodimer PARS1. However, the K_m value for proline and ATP in both settings are much smaller than physiological or cell culture conditions (Greiner & Glonek, 2021; Huang *et al*, 2010; Hutson *et al*, 1997; Toyoshima *et al*, 2017), which might be the reason for the similar aminoacylation activity between homodimer and heterodimer shown in Fig. 6C (experimental condition: 100 μM proline, 5 mM ATP).

(c) Have more through Ki determination studies been performed on PARS1 aminoacylation activity in the presence of DWN12088 inhibitor?

Answer:

Since a clear mode of inhibition is necessary for accurate determination of the Ki value through enzyme kinetic study, we were unable to determine the Ki value of DWN12088 through kinetic studies. Studies that determined IC_{50} values for DWN12088 with different substrate concentrations indicated that the compound is a competitive inhibitor for proline, and a mixed or uncompetitive inhibitor for ATP (Fig EV3). The complex inhibition mode of DWN12088 made it difficult to determine the Ki value through kinetic studies. Instead, we focused on the definition of Ki, the dissociation constant of an enzyme-inhibitor complex, and determined the dissociation constant using a microscale thermophoresis (MST) assay. The Kd values of DWN12088 were measured separately in the presence of ATP or proline concerning proline activation reaction. The Kd value of DWN12088 was 31 μM and

20 μM in SPR and MST assays, respectively, in the presence of ATP (Fig EV3H and Fig 6B), while it was 79 μM and 108 μM in the presence of proline (30 μM and 120 μM, respectively) (Figure below, n = 3, mean ± SD). Therefore, the Kd or Ki value of DWN12088 may be in the tens of μM range under physiological conditions.

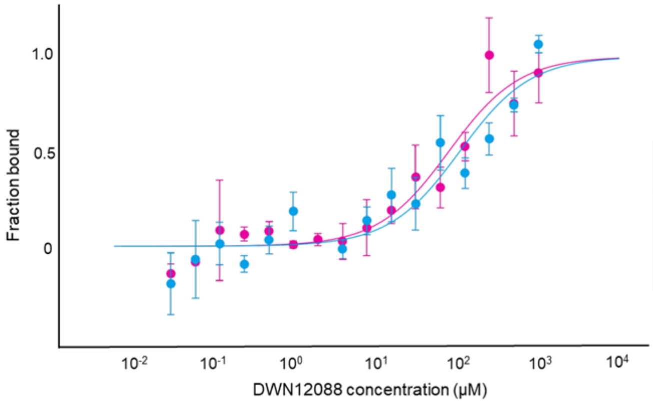

Protein	K_d for DWN 12088 (μM)
PARS1 with proline (30uM)	79.30 ± 31.22
PARS1 with proline (120uM)	108.18 ± 57.37

Minor points:

(a) The article, especially in the Introduction and Discussion, lacks detailed description/references on the improvement of the Therapeutic Index for other compounds targeting different diseases. This is important so the reader can judge how significant the improvement of the Safety Window of DWN12088 over Halgofuginone actually is.

Answer:

We revised the manuscript following the reviewer's recommendation by adding an example of theophylline and doxofylline in the Discussion section (Line 444-450).

(b) Is there anyway that the large number very similar panels in Figures 3 and 4 can

be reduced/ rationalized/ partially moved to supplementary work section to make it easier on the reader to follow the work?

Answer:

As the reviewer recommended, we moved some panels in Figures 3 and 4 to Fig EV 5. We wish that this change will make it easier on the reader to follow the work.

Referee #2 (Comments on Novelty/Model System for Author):

2. glutamyl-propyl-tRNA synthase is a established target in the context of pulmonary fibrosis since 2019.

4. Therapeutic, rather than the applied prophylactic, administration and/or the use of the Ad-TGFmodel, would streng results

Referee #2 (Remarks for Author):

Authors present in this paper a success story for controlling fibrosis through developing small molecule prolyl-tRNA synthetase 1 inhibitors endowed with a desired safety profile and a new binding mode (assymetric catalytic inhibitor). The authors went through a well-organized and conducted scrutiny, including medicinal chemistry, structural biology, in vitro, cell-based, cytotoxicity as well as in vivo bioefficacy and toxicity studies. Compound HF was used as a starting point, which suffers from serious toxicity issues. This scrutiny rendered them able to refine all

those molecular determinants providing both potency (in vitro and in vivo) and drug-like properties at the same time, giving rise to compound DWN12088, a clinical candidate currently being found in phase II trials. Of note, the improved therapeutic index (efficacy/toxicity) of DWN12088 in IPF models has been achieved, at least in part, through the unique mode of action of this compound. This includes DWN12088's binding to PARS1 homodimer in an asymmetric manner, thus inhibiting enzyme's two catalytic sites differently compared to its precursor HF.

There are some questions remaining to be addressed and which would strengthen the study:

1) Both 1H-NMR and 13C-NMR at least of the compounds displayed in Figure 3 must be provided in the supporting information file.

Answer:

As the reviewer's suggestion, we provided 1H-NMR and 13C-NMR of the DWN compounds (Figure 3 Source Data).

2) Under which criteria DWN2088 was administered in vivo at 10 mg/kg? Was a pharmacokinetic study performed first? Or the dose of administration was selected randomly?

Answer:

Pharmacokinetic and *in vivo* efficacy studies of DWN12088 were conducted at doses of 3, 10, and 30 mg/kg. In the efficacy test of DWN12088 in a bleomycin-induced

lung fibrosis model, we observed that the effect of DWN12088 was not further increased in mice administered with 30 mg/kg compared to those administered with 10 mg/kg (Fig EV4A to C) although plasma concentrations of DWN12088 were increased (Appendix Fig S1). Based on the results, we concluded that the efficacy of DWN12088 had reached a saturation point at a dose of 10 mg/kg, and this dose was used in subsequent experiments. We have included this explanation in the revised manuscript (Line 184-195).

3) In the in vivo experiment using the bleomycin mouse model, DWN2088's activity was evaluated at a prophylactic mode. The strength of the in vivo study as well as the experimental findings could be enhanced by testing compound's activity at a therapeutic mode (administration of the compound after fibrosis establishment). Given that the majority of drugs against fibroproliferative diseases are prescribed and given at late stages of the disease, evaluating the impact of DWN2088 on IPF after disease establishment would mean a lot about the therapeutic potential of this novel series of asymmetric inhibitors of prolyl-tRNA synthetase 1.

Answer:

A recent review has emphasized the importance of interventional timing in the bleomycin model of pulmonary fibrosis, suggesting that interventions beginning < 7days post-bleomycin are preventive models, while those beginning ≥ 7 days post-bleomycin are therapeutic models (Kolb *et al*, 2020). Thus, our model can be considered a therapeutic model.

However, we agree that the majority of drugs against fibroproliferative

diseases are prescribed and given at late stages of the disease. To examine the clinically relevant therapeutic potential of the compounds, we tested the efficacy of HF and DWN12088 by administering the compounds 14 days after bleomycin. Mice administered with HF 0.1 mg/kg or DWN12088 10 mg/kg showed a tendency to improve fibrotic symptoms such as SpO_2, hydroxyproline level, and BALF total cell count, although statistically significant changes ($P < 0.05$) were observed only in SpO_2 of DWN12088 administered mice (*P* values of the other measurements were around 0.06) (Fig EV4G to I).

We reasoned that the reduced efficacy might be due to the pathophysiology of BLM-induced lung fibrosis and mode of action of the drugs. Since spontaneous resolution begins 3 weeks post-intratracheal bleomycin and the rate of the collagen level increase is decreased from day 21 (Gharaee-Kermani *et al*, 2005; Liu *et al*, 2017; Liu *et al*, 2013; Phan *et al*, 1983), newly synthesized collagen, the targeting point of PARS1 inhibitors, would be decreased in the mouse model of administering the drugs two weeks after BLM challenge. We described the results obtained from the different animal models and suggested possible reasons for reduced efficacy and potential clinical implication in the revised manuscript (Line 415-428).

4) an additional fibrosis model, like Ad-TGFβ, would also strengthen findings, especially since it bypasses the inflammatory stage of BLM.

Answer:

We appreciate the reviewer's recommendation and agree with the strength of Ad-TGFβ model compared to the BLM model. However, we concerned that we could not

establish Ad-TGFβ model stably in our system within the limited period of revision. The Ad-TGFβ model is known for its highly variable and heterogeneous kinetics of injury, which makes it difficult to reproduce lesions consistently (Tashiro *et al*, 2017). Considering that the intratracheal injection of bleomycin-induced lung fibrosis model is acknowledged as "the best-characterized animal model available for preclinical testing" (Jenkins *et al*, 2017), our model system seems to be sufficient to compare effective doses between HF and DWN12088 for the major discovery point of our manuscript, "how DWN12088 overcomes safety issues at molecular mechanism despite targeting the catalytic site of prolyl-tRNA synthetase 1, an essential enzyme for protein synthesis".

Referee #3 (Remarks for Author):

Prolyl-tRNA synthetase 1 (PARS1) controls pathogenic accumulation of collagen in fibrotic diseases.

PARS1 regulates proline synthesis and can be a target for fibrotic diseased, however, regulation of proline synthesis may cause adverse effects on global protein synthesis. The authors validated that PARS1 worked as a positive regulator of collagen levels a positive regulator of collagen levels and developed a novel compound, DWN12088, which can be a candidate of therapeutic agent against fibrosis. The authors also showed binding mode of DWN12088 in the crystal structures.

The paper is well written, and the results are clear to discuss. I, therefore, recommend the manuscript will be accepted with small minor modifications.

Minor points to be corrected.

The authors should describe mode detail of X-ray diffraction data collection; such as beamline name, detector name,

Answer:

Following the reviewer's recommendation, we described beamline name and detector name in the Method section (Line 775-777).

In the crystallographic data table, Space group P 1 2(1) 1 can be P2(1).

Cell angle, alpha and gamma can be omitted, since these values are evident from the space group.

"I" should be italic in I/sigmaI

Line 691

Reference of coot should be Emsley et al. 2010. See,

https://www2.mrc-lmb.cam.ac.uk/personal/pemsley/coot/docs/coot-faq.html#Citing

Answer:

We corrected them in the revised manuscript (Line 1201).

References

Gharaee-Kermani M, Hatano K, Nozaki Y, Phan SH (2005) Gender-based differences in bleomycin-induced pulmonary fibrosis. *Am J Pathol* 166: 1593-1606

Greiner JV, Glonek T (2021) Intracellular ATP Concentration and Implication for Cellular Evolution. *Biology (Basel)* 10

Huang H, Zhang X, Li S, Liu N, Lian W, McDowell E, Zhou P, Zhao C, Guo H, Zhang C *et al* (2010)

Physiological levels of ATP negatively regulate proteasome function. *Cell Res* 20: 1372-1385

Hutson RG, Kitoh T, Moraga Amador DA, Cosic S, Schuster SM, Kilberg MS (1997) Amino acid control of asparagine synthetase: relation to asparaginase resistance in human leukemia cells. *Am J Physiol* 272: C1691-1699

Jenkins RG, Moore BB, Chambers RC, Eickelberg O, Konigshoff M, Kolb M, Laurent GJ, Nanthakumar CB, Olman MA, Pardo A *et al* (2017) An Official American Thoracic Society Workshop Report: Use of Animal Models for the Preclinical Assessment of Potential Therapies for Pulmonary Fibrosis. *Am J Respir Cell Mol Biol* 56: 667-679

Kolb P, Upagupta C, Vierhout M, Ayaub E, Bellaye PS, Gauldie J, Shimbori C, Inman M, Ask K, Kolb MRJ (2020) The importance of interventional timing in the bleomycin model of pulmonary fibrosis. *Eur Respir J* 55

Liu T, De Los Santos FG, Phan SH (2017) The Bleomycin Model of Pulmonary Fibrosis. *Methods Mol Biol* 1627: 27-42

Liu W, Wan J, Han JZ, Li C, Feng DD, Yue SJ, Huang YH, Chen Y, Cheng QM, Li Y *et al* (2013) Antiflammin-1 attenuates bleomycin-induced pulmonary fibrosis in mice. *Respir Res* 14: 101

Phan SH, Armstrong G, Sulavik MC, Schrier D, Johnson KJ, Ward PA (1983) A comparative study of pulmonary fibrosis induced by bleomycin and an O2 metabolite producing enzyme system. *Chest* 83: 44S-45S

Tashiro J, Rubio GA, Limper AH, Williams K, Elliot SJ, Ninou I, Aidinis V, Tzouvelekis A, Glassberg MK (2017) Exploring Animal Models That Resemble Idiopathic Pulmonary Fibrosis. *Front Med (Lausanne)* 4: 118

Toyoshima K, Nakamura M, Adachi Y, Imaizumi A, Hakamada T, Abe Y, Kaneko E, Takahashi S, Shimokado K (2017) Increased plasma proline concentrations are associated with sarcopenia in the elderly. *PLoS One* 12: e0185206

26th Apr 2023

Dear Prof. Kim,

Thank you for the submission of your revised manuscript to EMBO Molecular Medicine. I am pleased to inform you that we will be able to accept your manuscript pending the following final amendments:

1) In the main manuscript file, please do the following:
- In M&M, provide the antibody dilutions that were used for each antibody.
2) Synopsis: Please check your synopsis text and image before submission with your revised manuscript. Please be aware that in the proof stage minor corrections only are allowed (e.g., typos).
3) For more information: This space should be used to list relevant web links for further consultation by our readers. Could you identify some relevant ones and provide such information as well? Some examples are patient associations, relevant databases, OMIM/proteins/genes links, author's websites, etc...
4) Source data: Only numerical data should be in .xls format, western blots should be compiled in one .pdf file per figure. Please upload source data as one (zipped) file per figure.
5) As part of the EMBO Publications transparent editorial process initiative (see our Editorial at http://embomolmed.embopress.org/content/2/9/329), EMBO Molecular Medicine will publish online a Review Process File (RPF) to accompany accepted manuscripts. This file will be published in conjunction with your paper and will include the anonymous referee reports, your point-by-point response and all pertinent correspondence relating to the manuscript. Let us know whether you agree with the publication of the RPF and as here, if you want to remove or not any figures from it prior to publication. Please note that the Authors checklist will be published at the end of the RPF.
6) Please provide a point-by-point letter INCLUDING my comments as well as the reviewer's reports and your detailed responses (as Word file).

I look forward to reading a new revised version of your manuscript as soon as possible.

Yours sincerely,

Zeljko Durdevic

Zeljko Durdevic
Editor
EMBO Molecular Medicine

*** Instructions to submit your revised manuscript ***

*** PLEASE NOTE *** As part of the EMBO Publications transparent editorial process initiative (see our Editorial at https://www.embopress.org/doi/pdf/10.1002/emmm.201000094), EMBO Molecular Medicine will publish online a Review Process File to accompany accepted manuscripts.

In the event of acceptance, this file will be published in conjunction with your paper and will include the anonymous referee reports, your point-by-point response and all pertinent correspondence relating to the manuscript. If you do NOT want this file to be published, please inform the editorial office at contact@embomolmed.org.

When submitting your revised manuscript, please include:

1) a .docx formatted version of the manuscript text (including Figure legends and tables)

2) Separate figure files*

3) supplemental information as Expanded View and/or Appendix. Please carefully check the authors guidelines for formatting

Expanded view and Appendix figures and tables at
https://www.embopress.org/page/journal/17574684/authorguide#expandedview

4) a letter INCLUDING the reviewer's reports and your detailed responses to their comments (as Word file).

5) The paper explained: EMBO Molecular Medicine articles are accompanied by a summary of the articles to emphasize the major findings in the paper and their medical implications for the non-specialist reader. Please provide a draft summary of your article highlighting
- the medical issue you are addressing,
- the results obtained and
- their clinical impact.

This may be edited to ensure that readers understand the significance and context of the research.
Please refer to any of our published articles for an example.

6) For more information: There is space at the end of each article to list relevant web links for further consultation by our readers. Could you identify some relevant ones and provide such information as well? Some examples are patient associations, relevant databases, OMIM/proteins/genes links, author's websites, etc...

7) Author contributions: the contribution of every author must be detailed in a separate section.

8) EMBO Molecular Medicine now requires a complete author checklist (https://www.embopress.org/page/journal/17574684/authorguide) to be submitted with all revised manuscripts. Please use the checklist as guideline for the sort of information we need WITHIN the manuscript. The checklist should only be filled with page numbers were the information can be found. This is particularly important for animal reporting, antibody dilutions (missing) and exact values and n that should be indicted instead of a range.

9) Every published paper now includes a 'Synopsis' to further enhance discoverability. Synopses are displayed on the journal webpage and are freely accessible to all readers. They include a short stand first (maximum of 300 characters, including space) as well as 2-5 one sentence bullet points that summarise the paper. Please write the bullet points to summarise the key NEW findings. They should be designed to be complementary to the abstract - i.e. not repeat the same text. We encourage inclusion of key acronyms and quantitative information (maximum of 30 words / bullet point). Please use the passive voice. Please attach these in a separate file or send them by email, we will incorporate them accordingly.

You are also welcome to suggest a striking image or visual abstract to illustrate your article. If you do please provide a jpeg file 550 px-wide x 400-px high.

10) A Conflict of Interest statement should be provided in the main text

11) Please note that we now mandate that all corresponding authors list an ORCID digital identifier. This takes <90 seconds to complete. We encourage all authors to supply an ORCID identifier, which will be linked to their name for unambiguous name identification.

Currently, our records indicate that the ORCID for your account is 0000-0002-1570-3230.

Please click the link below to modify this ORCID:
Link Not Available

12) The system will prompt you to fill in your funding and payment information. This will allow Wiley to send you a quote for the article processing charge (APC) in case of acceptance. This quote takes into account any reduction or fee waivers that you may be eligible for. Authors do not need to pay any fees before their manuscript is accepted and transferred to our publisher.

*Additional important information regarding Figures

Each figure should be given in a separate file and should have the following resolution:
Graphs 800-1,200 DPI
Photos 400-800 DPI
Colour (only CMYK) 300-400 DPI"

Figures are not edited by the production team. All lettering should be the same size and style; figure panels should be indicated by capital letters (A, B, C etc). Gridlines are not allowed except for log plots. Figures should be numbered in the order of their appearance in the text with Arabic numerals. Each Figure must have a separate legend and a caption is needed for each panel.

*Additional important information regarding figures and illustrations can be found at https://bit.ly/EMBOPressFigurePreparationGuideline. See also figure legend preparation guidelines: https://www.embopress.org/page/journal/17574684/authorguide#figureformat

The system will prompt you to fill in your funding and payment information. This will allow Wiley to send you a quote for the article processing charge (APC) in case of acceptance. This quote takes into account any reduction or fee waivers that you may be eligible for. Authors do not need to pay any fees before their manuscript is accepted and transferred to our publisher.

***** Reviewer's comments *****

Referee #1 (Comments on Novelty/Model System for Author):

My reasons for the choices given above are outlined in my previous review of the earlier version of the manuscript. The changes introduced have increased the clarity of the manuscript.

Referee #1 (Remarks for Author):

The authors have responded to the questions raised in the previous review of the manuscript to my satisfaction.

Referee #2 (Comments on Novelty/Model System for Author):

The authors have sufficiently accommodated my concerns.

he uthors ddressed the re inin editori issues

3rd May 2023

Dear Prof. Kim,

We are pleased to inform you that your manuscript is accepted for publication and is now being sent to our publisher to be included in the next available issue of EMBO Molecular Medicine.

Please read below for additional IMPORTANT information regarding your article, its publication and the production process.

Congratulations on your interesting work,

Zeljko Durdevic

Zeljko Durdevic
Editor
EMBO Molecular Medicine

Follow us on Twitter @EmboMolMed
Sign up for eTOCs at embopress.org/alertsfeeds

*** *** *** IMPORTANT INFORMATION *** *** ***

SPEED OF PUBLICATION
The journal aims for rapid publication of papers, using using the advance online publication "Early View" to expedite the process: A properly copy-edited and formatted version will be published as "Early View" after the proofs have been corrected. Please help the Editors and publisher avoid delays by providing e-mail address(es), telephone and fax numbers at which author(s) can be contacted.

Should you be planning a Press Release on your article, please get in contact with embomolmed@wiley.com as early as possible, in order to coordinate publication and release dates.

LICENSE AND PAYMENT:

All articles published in EMBO Molecular Medicine are fully open access: immediately and freely available to read, download and share.

EMBO Molecular Medicine charges an article processing charge (APC) to cover the publication costs. You, as the corresponding author for this manuscript, should have already received a quote with the article processing fee separately. Please let us know in case this quote has not been received.

Once your article is at Wiley for editorial production you will receive an email from Wiley's Author Services system, which will ask you to log in and will present you with the publication license form for completion. Within the same system the publication fee can be paid by credit card, an invoice, pro forma invoice or purchase order can be requested.

Payment of the publication charge and the signed Open Access Agreement form must be received before the article can be published online.

PROOFS

You will receive the proofs by e-mail approximately 2 weeks after all relevant files have been sent o our Production Office. Please return them within 48 hours and if there should be any problems, please contact the production office at embopressproduction@wiley.com.

Please inform us if there is likely to be any difficulty in reaching you at the above address at that time. Failure to meet our

deadlines may result in a delay of publication.

All further communications concerning your paper proofs should quote reference number EMM-2022-16940-V3 and be directed to the production office at embopressproduction@wiley.com.

Thank you,

Zeljko Durdevic
Editor
EMBO Molecular Medicine

EMBO Press Author Checklist

Corresponding Author Name: Sunghoon Kim	
Journal Submitted to: EMBO Molecular Medicine	
Manuscript Number: EMM-2022-16940	

USEFUL LINKS FOR COMPLETING THIS FORM
The EMBO Journal - Author Guidelines
EMBO Reports - Author Guidelines
Molecular Systems Biology - Author Guidelines
EMBO Molecular Medicine - Author Guidelines

Reporting Checklist for Life Science Articles (updated January 2022)
This checklist is adapted from Materials Design Analysis Reporting (MDAR) Checklist for Authors. MDAR establishes a minimum set of requirements in transparent reporting in the life sciences (see Statement of Task: 10.31222/osf.io/9sm4x). Please follow the journal's guidelines in preparing your manuscript.
Please note that a copy of this checklist will be published alongside your article.

Abridged guidelines for figures
1. Data
The data shown in figures should satisfy the following conditions:
- the data were obtained and processed according to the field's best practice and are presented to reflect the results of the experiments in an accurate and unbiased manner.
- ideally, figure panels should include only measurements that are directly comparable to each other and obtained with the same assay.
- plots include clearly labeled error bars for independent experiments and sample sizes. Unless justified, error bars should not be shown for technical replicates.
- if n<5, the individual data points from each experiment should be plotted. Any statistical test employed should be justified.
- Source Data should be included to report the data underlying figures according to the guidelines set out in the authorship guidelines on Data Presentation.

2. Captions
Each figure caption should contain the following information, for each panel where they are relevant:
- a specification of the experimental system investigated (eg cell line, species name).
- the assay(s) and method(s) used to carry out the reported observations and measurements.
- an explicit mention of the biological and chemical entity(ies) that are being measured.
- an explicit mention of the biological and chemical entity(ies) that are altered/varied/perturbed in a controlled manner.
- the exact sample size (n) for each experimental group/condition, given as a number, not a range;
- a description of the sample collection allowing the reader to understand whether the samples represent technical or biological replicates (including how many animals, litters, cultures, etc.).
- a statement of how many times the experiment shown was independently replicated in the laboratory.
- definitions of statistical methods and measures:
  - common tests, such as t-test (please specify whether paired vs. unpaired), simple χ2 tests, Wilcoxon and Mann-Whitney tests, can be unambiguously identified by name only, but more complex techniques should be described in the methods section;
  - are tests one-sided or two-sided?
  - are there adjustments for multiple comparisons?
  - exact statistical test results, e.g., P values = x but not P values < x;
  - definition of 'center values' as median or average;
  - definition of error bars as s.d. or s.e.m.

Please complete ALL of the questions below.
Select "Not Applicable" only when the requested information is not relevant for your study.

Materials

Newly Created Materials	Information included in the manuscript?	In which section is the information available? (Reagents and Tools Table, Materials and Methods, Figures, Data Availability Section)
New materials and reagents need to be available; do any restrictions apply?	Not Applicable	Data availability

Antibodies	Information included in the manuscript?	In which section is the information available? (Reagents and Tools Table, Materials and Methods, Figures, Data Availability Section)
For **antibodies** provide the following information:<br>- Commercial antibodies: RRID (if possible) or supplier name, catalogue number and or/clone number<br>- Non-commercial: RRID or citation	Yes	Materials and Methods

DNA and RNA sequences	Information included in the manuscript?	In which section is the information available? (Reagents and Tools Table, Materials and Methods, Figures, Data Availability Section)
**Short novel DNA or RNA including primers, probes**: provide the sequences.	Yes	Materials and Methods

Cell materials	Information included in the manuscript?	In which section is the information available? (Reagents and Tools Table, Materials and Methods, Figures, Data Availability Section)
**Cell lines:** Provide species information, strain. Provide accession number in repository **OR** supplier name, catalog number, clone number, and/**OR** RRID.	Yes	Materials and Methods
**Primary cultures:** Provide species, strain, sex of origin, genetic modification status.	Yes	Materials and Methods
Report if the cell lines were recently **authenticated** (e.g., by STR profiling) and tested for mycoplasma contamination.	Yes	Materials and Methods

Experimental animals	Information included in the manuscript?	In which section is the information available? (Reagents and Tools Table, Materials and Methods, Figures, Data Availability Section)
**Laboratory animals or Model organisms:** Provide species, strain, sex, age, genetic modification status. Provide accession number in repository **OR** supplier name, catalog number, clone number, **OR** RRID.	Yes	Materials and Methods
**Animal observed in or captured from the field:** Provide species, sex, and age where possible.	Not Applicable	
Please detail **housing and husbandry conditions**.	Yes	Materials and Methods

Plants and microbes	Information included in the manuscript?	In which section is the information available? (Reagents and Tools Table, Materials and Methods, Figures, Data Availability Section)
**Plants:** provide species and strain, ecotype and cultivar where relevant, unique accession number if available, and source (including location for collected wild specimens).	Not Applicable	
**Microbes:** provide species and strain, unique accession number if available, and source.	Not Applicable	

Human research participants	Information included in the manuscript?	In which section is the information available? (Reagents and Tools Table, Materials and Methods, Figures, Data Availability Section)
If collected and within the bounds of privacy constraints report on age, sex and gender or ethnicity for all study participants.	Not Applicable	

Core facilities	Information included in the manuscript?	In which section is the information available? (Reagents and Tools Table, Materials and Methods, Figures, Data Availability Section)
If your work benefited from core facilities, was their service mentioned in the acknowledgments section?	Not Applicable	

Design

Study protocol	Information included in the manuscript?	In which section is the information available? (Reagents and Tools Table, Materials and Methods, Figures, Data Availability Section)
If study protocol has been **pre-registered, provide DOI in the manuscript**. For clinical trials, provide the trial registration number **OR** cite DOI.	Not Applicable	
Report the **clinical trial registration number** (at ClinicalTrials.gov or equivalent), where applicable.	Not Applicable	

Laboratory protocol	Information included in the manuscript?	In which section is the information available? (Reagents and Tools Table, Materials and Methods, Figures, Data Availability Section)
Provide DOI OR other citation details if **external detailed step-by-step protocols** are available.	Not Applicable	

Experimental study design and statistics	Information included in the manuscript?	In which section is the information available? (Reagents and Tools Table, Materials and Methods, Figures, Data Availability Section)
Include a statement about **sample size** estimate even if no statistical methods were used.	Yes	Materials and Methods
Were any steps taken to minimize the effects of subjective bias when allocating animals/samples to treatment (e.g. **randomization procedure**)? If yes, have they been described?	Yes	Materials and Methods
Include a statement about **blinding** even if no blinding was done.	Yes	Materials and Methods
Describe **inclusion/exclusion criteria** if samples or animals were excluded from the analysis. Were the criteria pre-established?<br><br>If sample or data points were omitted from analysis, report if this was due to attrition or intentional exclusion and provide justification.	Yes	Materials and Methods
For every figure, are **statistical tests** justified as appropriate? Do the data meet the assumptions of the tests (e.g., normal distribution)? Describe any methods used to assess it. Is there an estimate of variation within each group of data? Is the variance similar between the groups that are being statistically compared?	Yes	Figures

Sample definition and in-laboratory replication	Information included in the manuscript?	In which section is the information available? (Reagents and Tools Table, Materials and Methods, Figures, Data Availability Section)
In the figure legends: state number of times the experiment was **replicated** in laboratory.	Yes	
In the figure legends: define whether data describe **technical or biological replicates**.	Yes	

Ethics

Ethics	Information included in the manuscript?	In which section is the information available? (Reagents and Tools Table, Materials and Methods, Figures, Data Availability Section)
Studies involving **human participants**: State details of **authority granting ethics approval** (IRB or equivalent committee(s), provide reference number for approval.	Not Applicable	
Studies involving **human participants**: Include a statement confirming that **informed consent** was obtained from all subjects and that the experiments conformed to the principles set out in the WMA Declaration of Helsinki and the Department of Health and Human Services Belmont Report.	Not Applicable	
Studies involving **human participants:** For publication of **patient photos**, include a statement confirming that consent to publish was obtained.	Not Applicable	
Studies involving experimental **animals**: State details of **authority granting ethics approval** (IRB or equivalent committee(s), provide reference number for approval. Include a statement of compliance with ethical regulations.	Yes	Materials and Methods
Studies involving **specimen and field samples:** State if relevant **permits** obtained, provide details of authority approving study; if none were required, explain why.	Not Applicable	

Dual Use Research of Concern (DURC)	Information included in the manuscript?	In which section is the information available? (Reagents and Tools Table, Materials and Methods, Figures, Data Availability Section)
Could your study fall under dual use research restrictions? Please check biosecurity documents and list of **select agents and toxins** (CDC): https://www.selectagents.gov/sat/list.htm	Not Applicable	
If you used a select agent, is the security level of the lab appropriate and reported in the manuscript?	Not Applicable	
If a study is subject to dual use research of concern regulations, is the name of the **authority granting approval and reference number** for the regulatory approval provided in the manuscript?	Not Applicable	

Reporting

The MDAR framework recommends adoption of discipline-specific guidelines, established and endorsed through community initiatives. Journals have their own policy about requiring specific guidelines and recommendations to complement MDAR.

Adherence to community standards	Information included in the manuscript?	In which section is the information available? (Reagents and Tools Table, Materials and Methods, Figures, Data Availability Section)
State if relevant guidelines or checklists (e.g., **ICMJE, MIBBI, ARRIVE, PRISMA**) have been followed or provided.	Not Applicable	
For **tumor marker prognostic studies**, we recommend that you follow the **REMARK** reporting guidelines (see link list at top right). See author guidelines, under 'Reporting Guidelines'. Please confirm you have followed these guidelines.	Not Applicable	
For **phase II and III randomized controlled trials**, please refer to the **CONSORT** flow diagram (see link list at top right) and submit the CONSORT checklist (see link list at top right) with your submission. See author guidelines, under 'Reporting Guidelines'. Please confirm you have submitted this list.	Not Applicable	

Data Availability

Data availability	Information included in the manuscript?	In which section is the information available? (Reagents and Tools Table, Materials and Methods, Figures, Data Availability Section)
Have **primary datasets** been deposited according to the journal's guidelines (see 'Data Deposition' section) and the respective accession numbers provided in the Data Availability Section?	Yes	Data Availability
Were **human clinical and genomic datasets** deposited in a public access-controlled repository in accordance to ethical obligations to the patients and to the applicable consent agreement?	Not Applicable	
Are **computational models** that are central and integral to a study available without restrictions in a machine-readable form? Were the relevant accession numbers or links provided?	Not Applicable	
If publicly available data were reused, provide the respective **data citations in the reference list**.	Not Applicable	
