# Supplementary material for: Control of fibrosis with enhanced safety via asymmetric inhibition of prolyl‐tRNA synthetase 1
Source: EMBO Mol Med. 2023 May 22;15(7):e16940. doi: 10.15252/emmm.202216940 (PMC10331583; doi:10.15252/emmm.202216940)

Figure EV1A

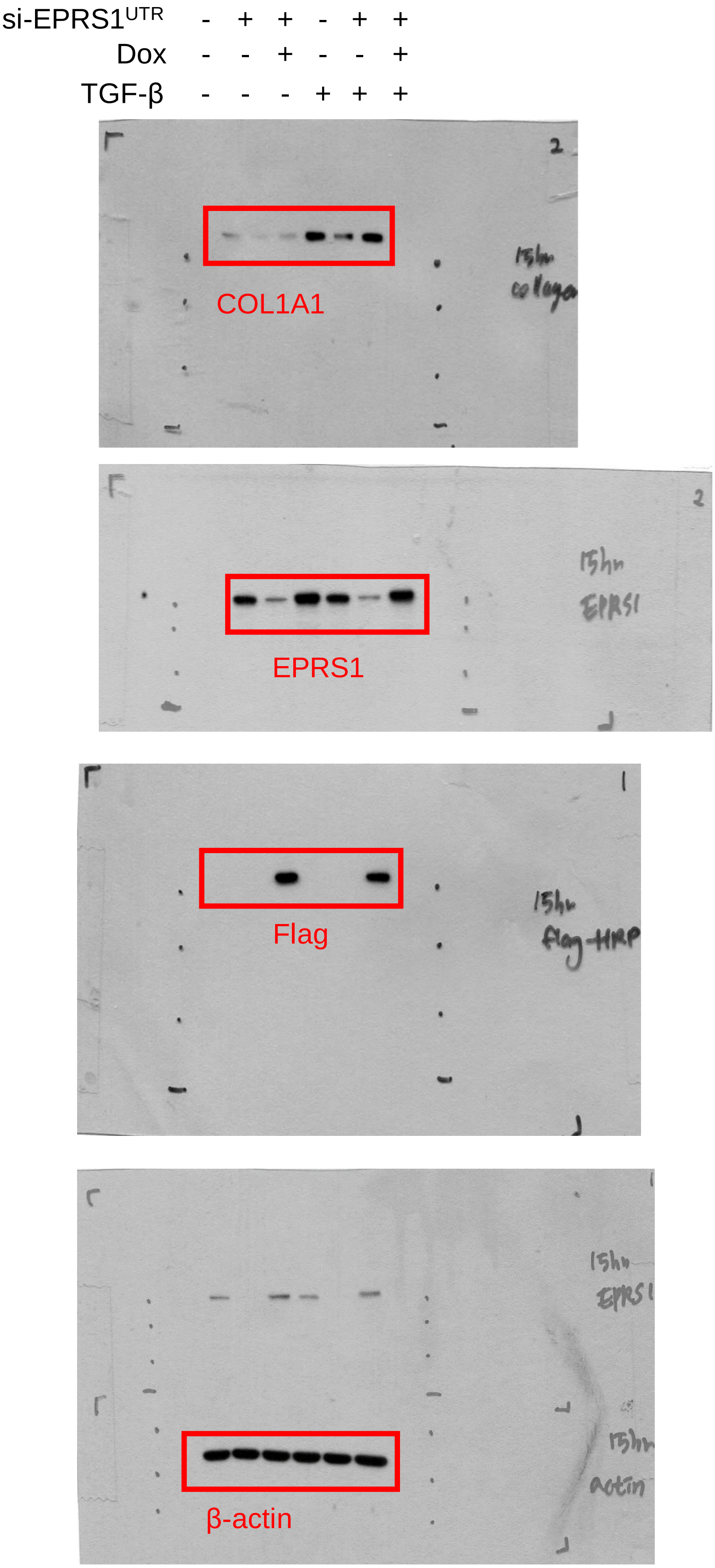

Supplement: Supplementary file 5 — Source Data for Figure 1 [file EMMM-15-e16940-s001.zip › Figure_EV1_Source_Data.zip!/EMM-2022-16940-Figure_EV1A_Source_Data.pdf]
