# Supplementary material for: Control of fibrosis with enhanced safety via asymmetric inhibition of prolyl‐tRNA synthetase 1
Source: EMBO Mol Med. 2023 May 22;15(7):e16940. doi: 10.15252/emmm.202216940 (PMC10331583; doi:10.15252/emmm.202216940)
Supplement: Supplementary file 5 — Source Data for Figure 1 [file EMMM-15-e16940-s001.zip › Figure_EV1_Source_Data.zip!/EMM-2022-16940-Figure_EV1C_Source_Data.pdf]

Figure EV1C

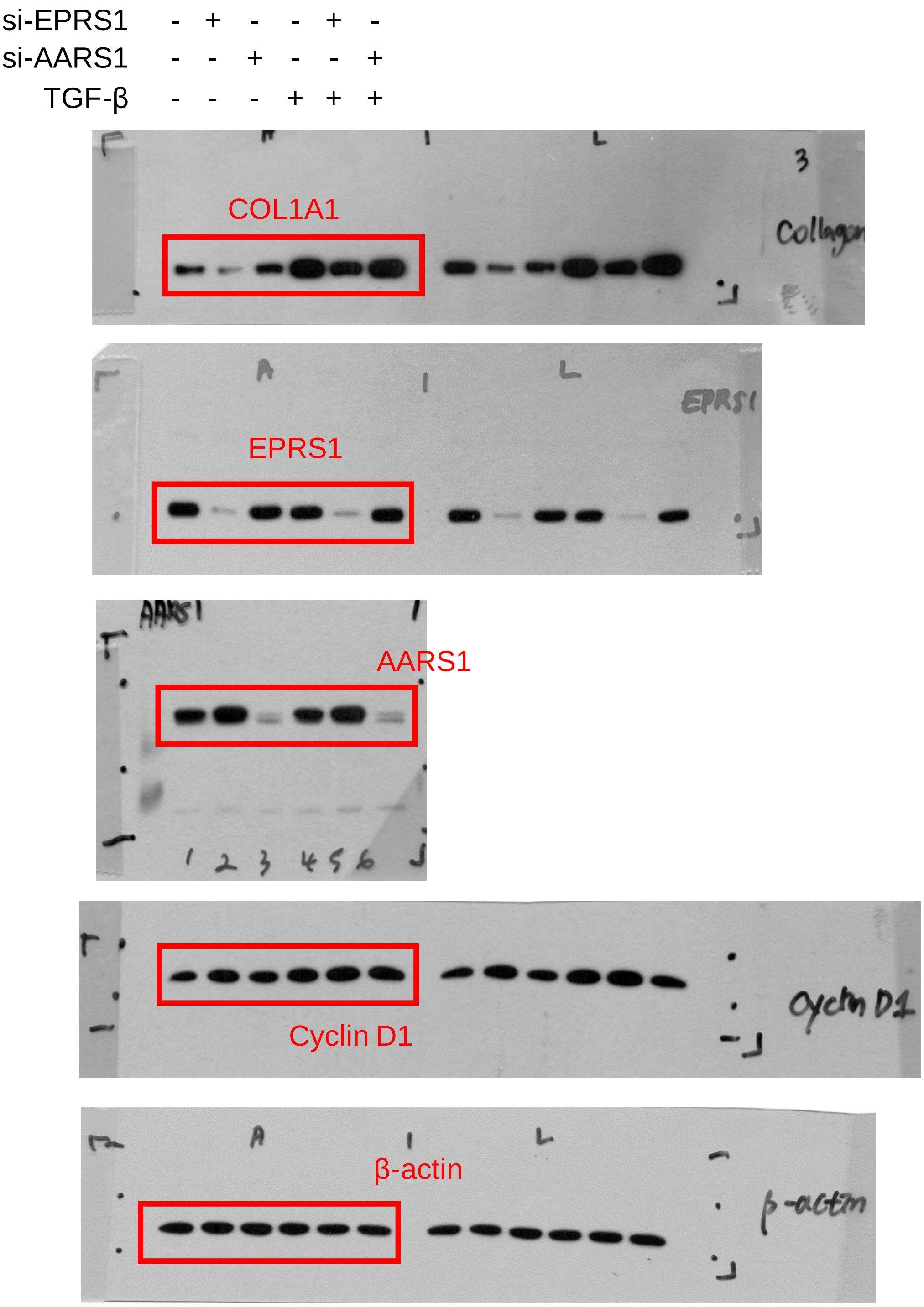
si-EPRS1 - + - - + -
si-AARS1 - - + - - +
TGF-β - - - + + +
COL1A1
Collagen
EPRS1
EPRS1
AARS1
AARS1
1 2 3 4 5 6
Cyclin D1
Cyclin D1
β-actin
β-actin
